# A network comparison on efficacy and safety profiling of PD-1/PD-L1 inhibitors in first-line treatment of advanced non-small cell lung cancer

**DOI:** 10.3389/fphar.2024.1516735

**Published:** 2025-01-06

**Authors:** Jie Fu, Yi-Dan Yan, Xu Wan, Xiao-Fan Sun, Xiu-Mei Ma, Ying-Jie Su

**Affiliations:** ^1^ Department of Pharmacy, Punan Branch of Renji Hospital, Shanghai Jiao Tong University School of Medicine, Shanghai, China; ^2^ Department of Pharmacy, Renji Hospital, Shanghai Jiao Tong University School of Medicine, Shanghai, China; ^3^ Department of Internal Medicine, Punan Branch of Renji Hospital, Shanghai Jiao Tong University School of Medicine, Shanghai, China; ^4^ Department of Internal Medicine, Renji Hospital, Shanghai Jiao Tong University School of Medicine, Shanghai, China; ^5^ Department of Radiation Oncology, Renji Hospital, School of Medicine, Shanghai Jiao Tong University, Shanghai, China

**Keywords:** programmed death-ligand 1, non-small cell lung cancer, immune checkpoint inhibitors, PD-1/PD-L1 inhibitors, network meta-analysis

## Abstract

**Objective:**

PD-1/PD-L1 inhibitors are novel immunotherapeutic agents that have been approved for first-line treatment in advanced non-small cell lung cancer (NSCLC). This study aims to evaluate the efficacy and safety of PD-1/PD-L1 inhibitors, which have completed phase 3 clinical trials, as a first-line treatment in patients with advanced NSCLC.

**Materials and methods:**

A systematic search of PubMed, Embase and the Cochrane Library was performed to extract eligible literature up to October 2023. Findings included overall survival (OS), objective response rate (ORR), progression-free survival (PFS), and grade ≥3 treatment-related adverse events (TRAEs). Furthermore, subgroup analyses were conducted based on PD-L1 expression levels and histological type.

**Results:**

We analyzed 29 studies including 18,885 patients. In analyses of all patients, penpulimab plus chemotherapy led the way for OS (HR 0.55, 95% CI: 0.40–0.75) and PFS (HR 0.43, 95% CI: 0.27–0.67). Regarding OS, for patients with PD-L1 expression ≥50%, 1%–49% and <1%, camrelizumab + chemotherapy (HR 0.48, 95% CI: 0.21–1.11), cemiplimab + chemotherapy (HR 0.50, 95% CI: 0.32–0.79) and nivolumab + ipilimumab (HR 0.64, 95% CI: 0.51–0.81) were considered optimal treatments. Compared with chemotherapy, monotherapy with nivolumab, cemiplimab, pembrolizumab, atezolizumab and durvalumab had lower odds of TRAE grade ≥3.

**Conclusion:**

In all patients, penpulimab plus chemotherapy was the most effective therapy, but treatment preferences varied by PD-L1 expression, histology type and associated outcomes. Safety at the individual patient level must be a high priority in the decision-making process. Further validation is warranted.

## 1 Introduction

Lung cancer has almost 2.48 million new cases and over 1.82 million deaths worldwide and ranks first in global cancer-related mortality (Ding et al., 2022; Bray et al., 2024). Accounting for about 85% of all cases of lung cancers (Siegel et al., 2021), non-small cell lung cancer (NSCLC) is the most common type of lung cancer. Advanced NSCLC is the leading contributor to cancer-related mortality worldwide (Rodak et al., 2021; Bray et al., 2024). For patients with advanced NSCLC that does not exhibit targetable mutations, the established first-line treatment protocol has been the administration of platinum-based chemotherapy. However, immune checkpoint inhibitors (ICIs), including anti-programmed cell death 1 (PD-1), programmed death ligand 1 (PD-L1) and cytotoxic T-lymphocyte-associated protein 4 (CTLA-4), have shown immense potential to further improve the prognosis of NSCLC patients (Chen et al., 2020), both as single agents and in combination therapies.

The use of immunotherapy, including immune checkpoint inhibitors, is now demonstrating considerable potential in the treatment of lung cancer, representing a significant advancement in the field of personalised medicine (Rao, 2024). PD-1/PD-L1 inhibitors have been rapidly approved and recognized by Food and Drug Administration (FDA) due to their excellent clinical outcomes, significantly prolonged survival, and relatively low incidence of side effects (Da et al., 2022; Ding et al., 2023; Srivastava et al., 2024). In 2015, 2015 and 2016, respectively, the FDA approved nivolumab, pembrolizumab and atezolizumab for the treatment of advanced NSCLC (Santabarbara et al., 2016). A growing number of PD-1/PDL-1 inhibitors, including camrelizumab, sintilimab, sugemalimab and so on, have been launched or are in clinical studies. These results of clinical trials have significantly changed the routine management of advanced or metastatic NSCLC. To date, PD-1/PD-L1 inhibitors with or without chemotherapy has become the first-line treatment strategy for NSCLC without driver mutations (Takada et al., 2022).

With the clinical trials of novel PD-1/PD-L1 inhibitors and the results of updated long-term follow-up studies bring new possibilities for advanced NSCLC. Despite the multitude of large clinical trials that have been conducted, determining the optimal treatment regimen in practice remains challenging due to an absence of direct comparisons between these trials and substantial variability, particularly with regard to the diverse PD-L1 expression, monotherapies versus combinations of therapies, histological types, and endpoints measured. The aim of this network of randomized controlled trails is to evaluate the safety and efficacy of PD-1/PD-L1 inhibitors in first-line treatments for advanced NSCLC.

## 2 Methods

### 2.1 Data sources and surveys

The NMA has been registered on the PROSPERO database under the identifier CRD42021252956. A systematic search of the PubMed, Embase and Cochrane Library databases was conducted up to 1 October 2023. The search was limited to English-language sources. The search terms are listed in Supplementary Table 1. The article underwent an independent screening process conducted by two co-authors (J. F., Y. S.), with a third author involved in resolving disagreements.

### 2.2 Eligibility criteria

Eligibility criteria for published trials were: (Ⅰ) phase Ⅲ RCTs in patients with previously untreated advanced or metastatic NSCLC; (Ⅱ) PD-1/PD-L1 inhibitors as a single agent or in combination with other drugs as an intervention arm; and (Ⅲ) detailed PFS or OS data. If both articles cover the same trial and the subsequent article updates key data such as overall survival, both articles should combine the most recent and accurate data for each outcome.

### 2.3 Study outcomes, data extraction, and quality assessment

The treatment effects of the patients, including PFS, OS and ORR, were assessed in our study. PFS and OS were evaluated using hazard ratios (HRs) and their 95% confidence intervals (CIs) as measures, which were derived from a direct extraction of the studies. The secondary outcome of interest was any treatment-related adverse events (TRAEs). We foused on treatment related severe AEs, defined as grade 3 or higher. Two investigators independently applied a predesigned information sheet to extract data independently, including name of trial, year of publication, first author, patient characteristics, clinical characteristics, intervention type and treatment outcomes including PFS, OS, ORR, and TRAEs. The Cochrane risk of bias tool (Cumpston et al., 2019) for randomized controlled trials was used to assess the quality and risk of bias of the involved trials. Each domain was rated as low, high or unclear. Disagreements were resolved by a third reviewer.

### 2.4 Statistical analysis

A network meta-analysis was conducted using the frequentist weighted least squares approach in our study. Depending on the heterogeneity among studies, either a fixed-effect or a random-effects model was employed. The I^2^ test was used to assess statistical heterogeneity, with a result exceeding 75% indicating significant heterogeneity. Logarithmic conversion was used to calculate the OR and HR. Chemotherapy served as a standard reference in the model due to its prevalent use in trials targeting wild-type EGFR/ALK NSCLC patients. The results were performed using R software (version 4.1.3, Command: netmeta, Package: netmeta). Statistical significance was defined as a p-value < 0.05. We ranked PD-1/PD-L1 inhibitors and chemotherapy using the P-score (0–1), derived from network estimates’ point estimates and standard errors, reflecting the mean extent of certainty that one intervention’s superiority over others.

## 3 Results

### 3.1 Study selection and characteristics

**FIGURE 1:**
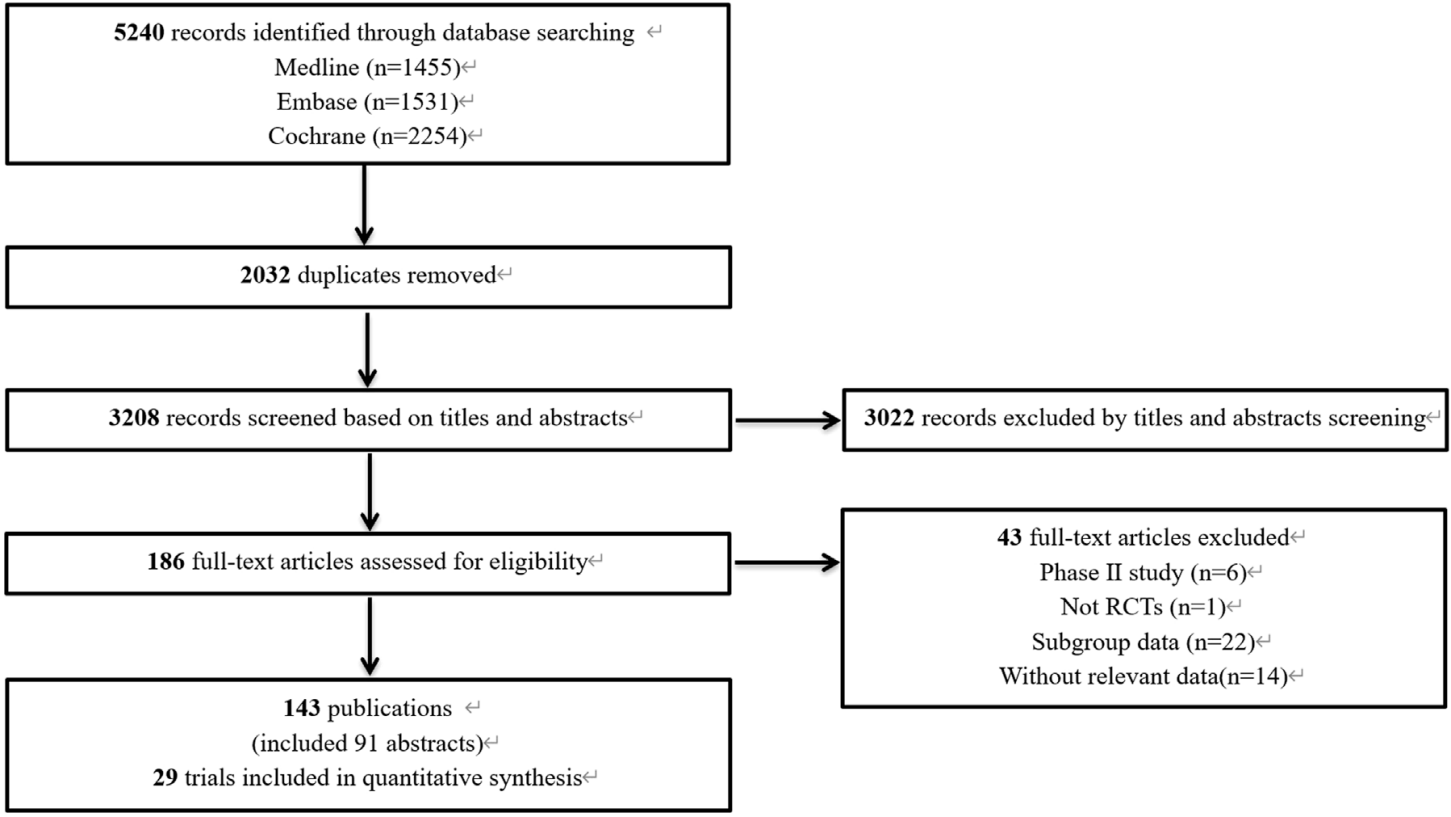
Flowchart of the selection algorithm and screening process.

**FIGURE 2:**
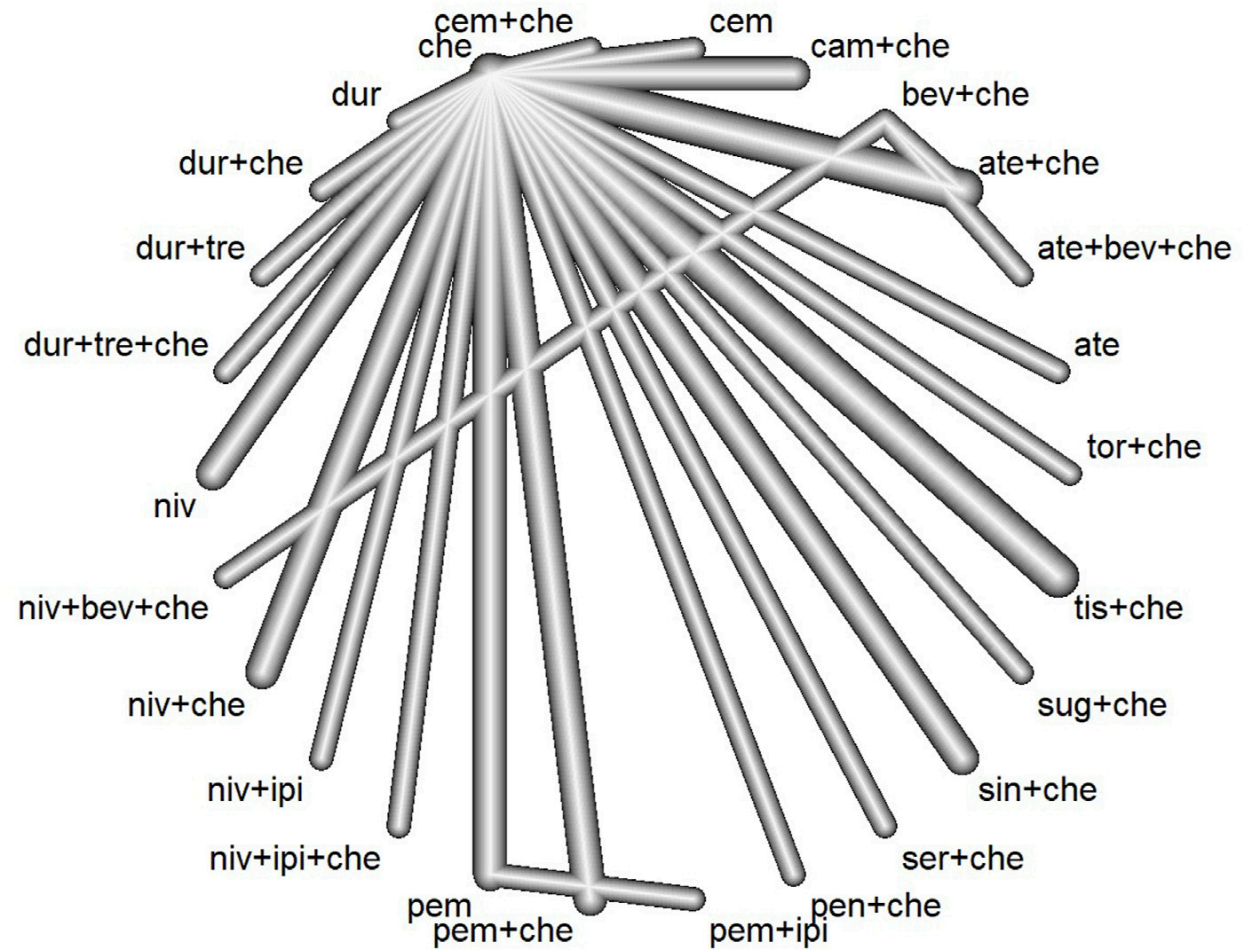
Network plot for the primary endpoint of overall survival (ate, atezolizumab; bev, bevacizumab; che, chemotherapy; cam, camrelizumab; cem, cemiplimab; dur, durvalumab; tre, tremelimumab; niv, nivolumab; ipi, ipilimumab; pem, pembrolizumab; pen, penpulimab; ser, serplulimab; sin:sintilimab; sug, sugemalimab; tis, tislelizumab; tor, toripalimab).

The preliminary database search yielded 5,240 articles, of which 1,455 were from PubMed, 1,531 from Embase, and 2,254 from the Cochrane Library. After the removal of 2,032 duplicate records, 3,208 articles underwent screening. The results of the preliminary search and screening process are illustrated in Figure 1. Ultimately, 29 RCTs that involved 18,885 patients and 27 treatment regimens fulfilled the inclusion criteria. The main characteristics are listed in Table 1. The included trials were updated prior to submission of the manuscript. Experimental arms in 7 trials studied consisted of PD-1/PD-L1 monotherapies [CheckMate 026 (Carbone et al., 2017), CheckMate 227-part 1 (Brahmer et al., 2023), EMPOWER-Lung 1 (Ozguroglu et al., 2023), IMpower110 (Jassem et al., 2021), KEYNOTE-024 (Reck et al., 2021), KEYNOTE-042 (de Castro et al., 2023), MYSTIC(Rizvi et al., 2020)]. Experimental arms in 19 trials studied 2 classes of drugs combination regimens, PD-1/PD-L1 inhibitors in combination with chemotherapy or a CTLA-4 antibody [AK105-302 (Zhong et al., 2024), ASTRUM-004 (Zhou et al., 2024), CameL (Zhou et al., 2021a), CameL-sq (Ren et al., 2022), CheckMate 227-part 1 (Brahmer et al., 2023), CheckMate 227-part 2 (Borghaei et al., 2023), CHOICE-01 (Wang et al., 2023), EMPOWER-Lung 3 (Makharadze et al., 2023), GEMSTONE-302 (Zhou et al., 2023), IMpower130 (West et al., 2019), IMpower131 (Jotte et al., 2020), IMpower132 (Nishio et al., 2020), IMpower150 (Socinski et al., 2021), KEYNOTE-189 (Garassino et al., 2023), KEYNOTE-407 (Novello et al., 2023), KEYNOTE-598 (Boyer et al., 2021), MYSTIC, ORIENT-11 (Zhang et al., 2022), ORIENT-12 (Zhou et al., 2021b), POSEIDON(Johnson et al., 2023), RATIONALE 304 (Lu et al., 2021), RATIONALE 307 (Wang et al., 2021)]. There was also a trial evaluating PD-1 inhibitors in combination with CTLA-4 antibody and chemotherapy [CheckMate 9LA (Carbone et al., 2024), POSEIDON]. Network diagram for the included studies of OS is shown in Figure 2. Of these, 22 trials presented data regarding survival in patients with PD-L1 high expression (≥50%) and 13 trials did so in patients with PD-L1 low expression (1%–49%). Overall, 29 trials were considered to have low risk of bias. Of the included RCTs, 16 open-label trials were rated at high risk of bias because they did not meet the criteria for performance bias. Ten trials (AK105-302, ASTRUM-004, CameL-sq, CHOICE-01, EMPOWER-Lung 3, GEMSTONE-302, KEYNOTE-189, KEYNOTE-407, ORIENT-11, ORIENT-12) were assessed as having a low risk of bias across six domains. Supplementary Table 2 shows the details of the quality assessment.

**TABLE 1:** Summary characteristics of the included studies.

Year	Trial	Population	Design	Type	Experimental group	Control group	Patient numbers	Endpoints	EGFR/ALK mutation	PD-L1 staining antibody clone	PD-L1 threshold values
2024	AK105-302	Stage IIIB–IV sq-NSCLC	Phase 3 double-blind	Article	penpulimab + chemotherapy	chemotherapy	175 vs. 175	PFS, OS, ORR, DOR, safety	—	SAB-028	TPS: 1%, 1%–49%, 50%
2024	ASTRUM-004	Stage IIIB–IV sq-NSCLC	Phase 3 double-blind	Article	serplulimab + chemotherapy	chemotherapy	358 vs. 179	PFS, OS, ORR, DOR, safety	—	22C3	TPS: 1%, 1%–49%, 50%
2021	CameL	Stage IIIB–IV nsq-NSCLC	Phase 3 open-label	Article	camrelizumab + chemotherapy	chemotherapy	205 vs. 207	PFS, OS, ORR, DOR, safety	—	22C3	TPS: 1%, 1%–49%, 50%
2022	CameL-sq	Stage IIIB–IV sq-NSCLC	Phase 3 double-blind	Article	camrelizumab + chemotherapy	chemotherapy	193 vs. 196	PFS, OS, ORR, DOR, safety	—	E1L3N	TPS: 1%, 1%–49%, 50%
2017	CheckMate 026	Stage IV/recurrent NSCLC	Phase 3 open-label	Article	nivolumab	chemotherapy	271 vs. 270	PFS, OS, ORR, safety	—	28-8	TPS: ≥1%,≥5%, ≥50%
2021	CheckMate 227-part 1	Stage IV/recurrent NSCLC	Phase 3 open-label	Article	PD-L1 ≥ 1%: nivolumab + ipilimumab nivolumab PD-L1 ˂ 1%: nivolumab + ipilimumab nivolumab + chemotherapy	chemotherapy	PD-L1 ≥ 1%: 396 vs. 396 vs. 397 PD-L1 ˂ 1%: 187 vs. 177 vs. 186	PFS, OS, ORR, DOR, safety	—	28-8	TPS: 1%, 1%–49%, 50%
2023	CheckMate 227-part 2	Stage IV/recurrent NSCLC	Phase 3 open-label	Article	nivolumab + chemotherapy	chemotherapy	377 vs. 378	PFS, OS, ORR, DOR, safety	—	28-8	TPS: 1%, 50%
2024	CheckMate 9LA	Stage IV/recurrent NSCLC	Phase 3 open-label	Article	nivolumab + ipilimumab + chemotherapy	chemotherapy	361 vs. 358	PFS, OS, ORR, DOR, safety	—	28-8	TPS: 1%, 1%–49%, 50%
2021	CHOICE-01	Stage IIIB–IV NSCLC	Phase 3 double-blind	Article	toripalimab + chemotherapy	chemotherapy	309 vs. 156	PFS, OS, ORR, DOR, safety	—	JS311	TC: 1%, 1%–49%, 50%
2023	EMPOWER-Lung 1	Stage IIIB–IV NSCLC	Phase 3 open-label	Article	cemiplima	chemotherapy	356 vs. 354	PFS, OS, ORR, DOR, safety	—	22C3	TPS: ≥50%
2023	EMPOWER-Lung 3	Stage IIIB–IV NSCLC	Phase 3 double-blind	Article	cemiplimab + chemotherapy	chemotherapy	312 vs. 154	PFS, OS, ORR, DOR, safe	—	SP263	TC: 1%, 1%–49%, 50%
2023	GEMSTONE-302	Metastatic NSCLC	Phase 3 double-blind	Article	sugemalimab + chemotherapy	chemotherapy	320 vs. 159	PFS, OS, ORR, DOR, safety	—	SP263	TPC: 1%, 1%–49%, 50%
2021	IMpower110	Stage IV NSCLC	Phase 3 open-label	Article	atezolizumab	chemotherapy	277 vs. 277	OS, PFS, ORR, DOR, safety	—	SP142,SP263,22C3	TC: 1%, 5%, 50%; IC: 1%, 5%, 10%
2019	IMpower130	Stage IV nsq-NSCLC	Phase 3 open-label	Article	atezolizumab + chemotherapy	chemotherapy	451 vs. 228	PFS, OS, ORR, DOR, safety	—	SP142	TC: 1%, 1%–49%, 50%; IC: 1%, 5%, 10%
2020	IMpower131	Stage IV sq-NSCLC	Phase 3 open-label	Article	atezolizumab + carboplatin + paclitaxel (A + CP) atezolizumab + carboplatin + nab-paclitaxel (A + CnP)	carboplatin + nab-paclitaxel (CnP)	338 vs. 343 vs. 340	PFS, OS, ORR, DOR, safety	—	SP142	TC: 1%, 5%, 50%; IC: 1%, 5%, 10%
2020	IMpower132	Stage IV nsq-NSCLC	Phase 3 open-label	Article	atezolizumab + chemotherapy	chemotherapy	292 vs. 286	PFS, OS, ORR, DOR, safety	—	SP142	TC: 1%, 5%, 50%; IC: 1%, 5%, 10%
2021	IMpower150	Stage IV/recurrent metastatic nsq-NSCLC	Phase 3 open-label	Article	atezolizumab + chemotherapy atezolizumab + bevacizumab + chemotherapy	bevacizumab + chemotherapy	356 vs. 348 vs. 336	PFS, OS, ORR, DOR, safety	—	SP142, SP263	TC: 1%, 5%, 50%; IC: 1%, 5%, 10%
2021	KEYNOTE-024	Stage IIIB–IV NSCLC	Phase 3 open-label	Article	pembrolizumab	chemotherapy	154 vs. 151	PFS, OS, ORR, DOR, safety	—	22C3	TPS: ≥50%
2023	KEYNOTE-042	Locally advanced/metastatic NSCLC	Phase 3 open-label	Article	pembrolizumab	chemotherapy	637 vs. 637	PFS, OS, ORR, DOR, safety	—	22C3	TPS: 1%–49%, 50%
2023	KEYNOTE-189	Metastatic nsq-NSCLC	Phase 3 double-blind	Article	pembrolizumab + chemotherapy	chemotherapy	410 vs. 206	PFS, OS, ORR, DOR, safety	—	22C3	TPS: 1%, 1%–49%, 50%
2023	KEYNOTE-407	Stage IV sq-NSCLC	Phase 3 double-blind	Article	pembrolizumab + chemotherapy	chemotherapy	278 vs. 281	PFS, OS, ORR, DOR, safety	—	22C3	TPS: 1%, 1%–49%, 50%
2021	KEYNOTE-598	Stage IV NSCLC	Phase 3 double-blind	Article	pembrolizumab + ipilimumab	pembrolizumab	284 vs. 284	PFS, OS, ORR, DOR, safety	—	22C3	TPS: ≥50%
2020	MYSTIC	Stage IV NSCLC	Phase 3 open-label	Article	durvalumab durvalumab + tremelimumab	chemotherapy	374 vs. 372 vs. 372	PFS, OS, ORR, DOR, safety	—	SP263	TC: 1%, 25%, 50%
2023	ONO-4538-52/TASUKI-52	Stage IIIB–IV/recurrent nsq-NSCLC	Phase 3 double-blind	Article	nivolumab + bevacizumab + chemotherapy	bevacizumab + chemotherapy	275 vs. 275	PFS, OS, ORR, DOR, safety	—	28-8	TPS: 1%, 1%–49%, 50%
2022	ORIENT-11	Stage IIIB–IV nsq-NSCLC	Phase 3 double-blind	Article	sintilimab + chemotherapy	chemotherapy	266 vs. 131	PFS, OS, ORR, DOR, safety	—	22C3	TPS: 1%, 1%–49%, 50%
2021	ORIENT-12	Stage IIIB–IV sq-NSCLC	Phase 3 double-blind	Article	sintilimab + chemotherapy	chemotherapy	179 vs. 178	PFS, OS, ORR, DOR, safety	—	22C3	TPS: 1%, 1%–49%, 50%
2023	POSEIDON	Metastatic NSCLC	Phase 3 open label	Article	durvalumab + tremelimumab + chemotherapy durvalumab + chemotherapy	chemotherapy	338 vs. 338 vs. 337	PFS, OS, ORR, DOR, safety	—	SP263	TC: 1%, 50%
2022	RATIONALE 304	Stage IIIB–IV nsq-NSCLC	Phase 3 open-label	Article	tislelizumab + chemotherapy	chemotherapy	223 vs. 111	PFS, OS, ORR, DOR, safety	—	SP263	TC: 1%, 1%–49%, 50%
2021	RATIONALE 307	Stage IIIB–IV sq-NSCLC	Phase 3 open-label	Article	tislelizumab + paclitaxel + carboplatin tislelizumab + nab-paclitaxel + carboplatin	paclitaxel + carboplatin	120 vs. 119 vs. 121	PFS, OS, ORR, DOR, safety	—	SP263	TC: 1%, 1%–49%, 50%

PFS, progression-free survival; OS, overall survival; ORR, objective response rate; DOR, duration of response; sq-NSCLC, squamous non-small cell lung cancer; nsq-NSCLC, non-squamous non-small cell lung cancer; NSCLC, non-small cell lung cancer; TPS, tumor proportion score; TC, tumour cells; IC, immune cells.

### 3.2 Treatment outcomes

#### 3.2.1 Overall survival

**FIGURE 3:**
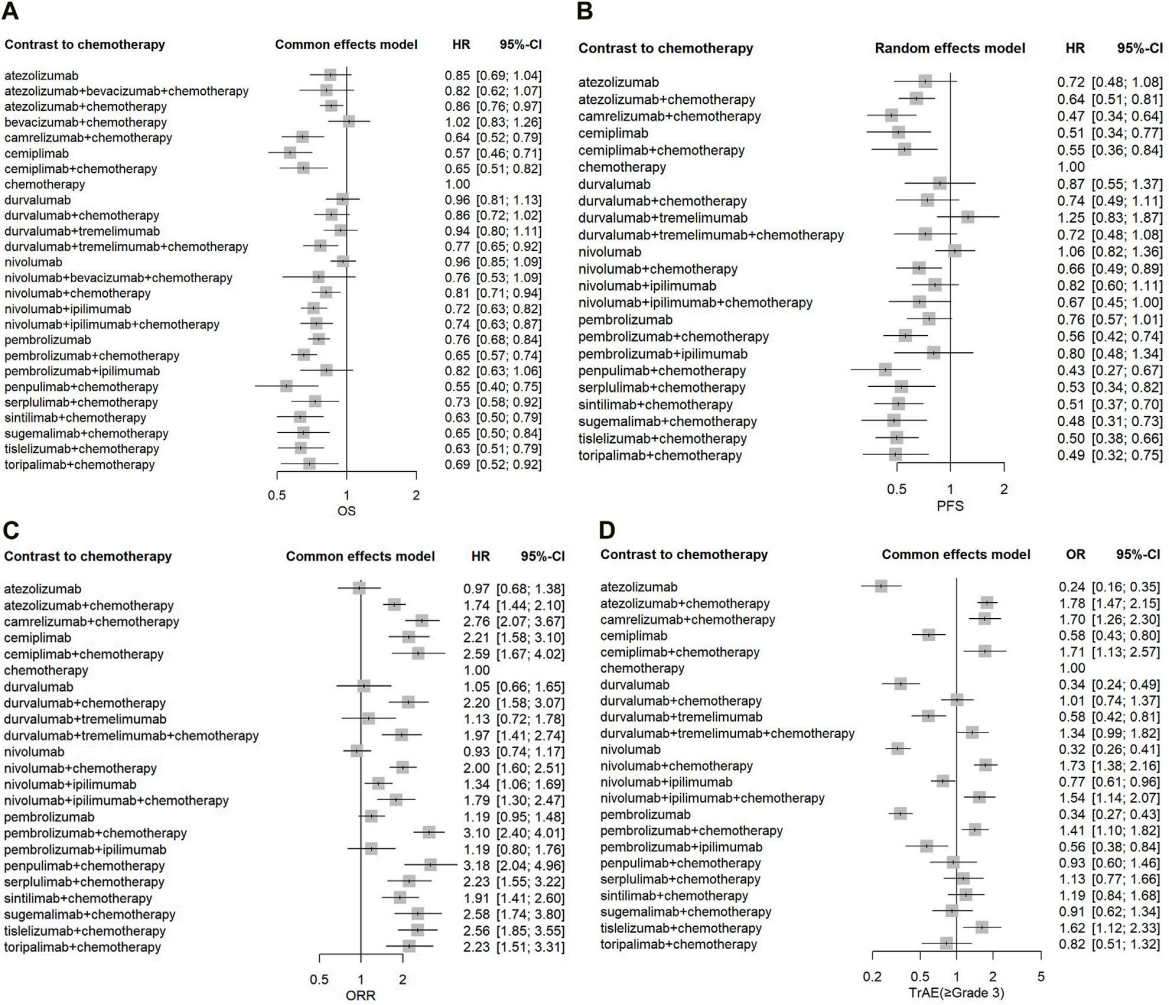
Forest plots for endpoints in main model. **(A)** Hazard ratio for overall survival. **(B)** Hazard ratio for progression-free survival. **(C)** Odds ratio for objective response rate. **(D)** Odds ratio for treatment related adverse events (≥Grade 3). HR, hazard ratio; Cl, confidence interval.

The hazard ratios of OS were assessed for 29 studies with a total of 18,191 patients in our network meta-analysis. In the analysis model, the HR for OS varied between 0.55 and 1.02, with a median value of 0.76. Low inconsistency between Q statistic and heterogeneity test at each level (I^2^ = 0%; total p = 0.4538; within designs, p = 0.4538) (Supplementary Figure S1). Compared with chemotherapy, the OS benefit was greatest with pen + che (HR = 0.55, 95%CI: 0.40–0.75), followed by cem (HR = 0.57, 95%CI: 0.41–0.71), and sin + che (HR = 0.63, 95%CI = 0.50–0.79) (Figure 3A). Atezolizumab (HR = 0.85, 95%CI: 0.69–1.04), ate + bev + che (HR = 0.82, 95%CI: 0.62–1.07), durvalumab (HR = 0.96, 95%CI: 0.81–1.13), dur + che (HR = 0.86, 95%CI: 0.72–1.02), dur + tre (HR = 0.94, 95%CI: 0.80–1.11), nivolumab (HR = 0.96, 95%CI: 0.85–1.09), niv + bev + che (HR = 0.76, 95%CI: 0.53–1.09), and pem + ipi (HR = 0.82, 95%CI: 0.63–1.06) did not show statistically significant differences compared with chemotherapy. Treatment regimens with PD-1/PD-L1 inhibitors in combination with chemotherapy or CTLA-4 antibody demonstrated a more pronounced trend towards improved OS compared to chemotherapy. With the exception of pembrolizumab and cemiplimab, there were no significant differences between PD-1/PD-L1 inhibitors monotherapy and chemotherap. The results of the pairwise comparisons of Network Meta-analyses for OS are shown in Supplementary Table 3.

#### 3.2.2 Progression-free survival

The hazard ratios of PFS were assessed for 27 studies with a total of 17,295 patients. In the network meta-analysis, the HR for PFS varied between 0.43 and 1.25, with a median value of 0.66. Moderated inconsistency between Q statistic and heterogeneity test at each level (I^2^ = 76.4%; total p < 0.0001; within designs, p < 0.0001; between designs, P = 0.5816). Pen + che regimen (HR = 0.43, 95% CI: 0.27–0.67) provided the greatest benefit in terms of PFS, followed by the cam + che (HR = 0.47, 95%CI: 0.34–0.64), and the sug + che (HR = 0.48, 95%CI: 0.31–0.73) (Figure 3B). Most two-drug combination regimens or three-drug combination regimens have better PFS outcomes compared to chemotherapy regimens. In the monotherapy, there were no significant differences between PD-1/PD-L1 drugs with chemotherapy, except for cemiplimab. The results of the pairwise comparisons of Network Meta-analyses for PFS are shown in Supplementary Table 4.

#### 3.2.3 Objective response rate

By indirect comparison, we analyzed differences in ORR between trials using anti-PD1 or anti-PD-L1 drugs (Figure 3C). The Odds ratio of ORR were evaluated in 27 studies with 23 treatment arms (Supplementary Figure S3). The pem + che (OR = 3.10, 95% CI: 2.40–4.01) regimen showed a significantly higher OR of ORR than the chemotherapy regimen alone, followed by the pen + che (OR = 3.18; 95% CI: 2.04–4.96) and the cam + che (OR = 2.76, 95%CI: 2.07–3.67).

#### 3.2.4 Safety

Safety was considered in regard to grade ≥ 3 treatment related adverse events. Regarding PD-1/PD-L1 inhibitors, the analysis of safety was based on 27 trials. The monotherapy such as atezolizumab (OR = 0.24; 95% CI: 0.16–0.35), nivolumab (OR = 0.32; 95% CI: 0.26–0.41), durvalumab (OR = 0.34; 95% CI: 0.24–0.49), pembrolizumab (OR = 0.34; 95% CI: 0.27–0.43) and cemiplimab (OR = 0.58; 95% CI: 0.43–0.80) were less associated with grade ≥3 TRAEs compared with chemotherapy. Nonetheless, some PD-1/PD-L1 inhibitors in combination with chemotherapy such as ate + che (OR = 1.78; 95% CI: 1.47–2.15), niv + che (OR = 1.73; 95% CI: 1.39–2.17), cam + che (OR = 1.70; 95% CI: 1.26–2.30), cem + che (OR = 1.71; 95% CI: 1.13–2.57), tis + che (OR = 1.62; 95% CI: 1.12–2.33), pem + che (OR = 1.41; 95% CI: 1.10–1.82) and more frequently associated with grade ≥3 TRAEs compared with chemotherapy. For the rate of grade 3–5 AE, dur + che (OR = 1.01; 95% CI: 0.74–1.37), dur + tre + che (OR = 1.34; 95% CI: 0.99–1.82), pen + che (OR = 0.93; 95% CI: 0.60–1.46), ser + che (OR = 1.13; 95% CI: 0.77–1.46), sin + che (OR = 1.19; 95% CI: 0.84–1.68), sug + che (OR = 0.91; 95% CI: 0.62–1.34) and tor + che (OR = 0.82; 95% CI: 0.52–1.31) were not significantly different compared to chemotherapy.

#### 3.2.5 Histology type

As currently the most readily available outcome in the pathological diagnosis of lung cancer, a total of 28 trials reported on the histological types, with 13 studies featuring a mixed histological profile, 8 studies exclusively focusing on non-squamous NSCLC, and 7 studies solely examining squamous NSCLC.

##### 3.2.5.1 Non-squamous NSCLC

Most treatment arms in the study showed a superior OS compared to chemotherapy in a direct analysis of OS in patients with non-squamous NSCLC (Supplementary Figure 4). Remarkably, tor + che (HR = 0.48; 95% CI: 0.32–0.72), pem + che (HR = 0.60; 95% CI: 0.50–0.72), and atezolizumab monotherapy (HR = 0.62; 95% CI: 0.40–0.96) demonstrated the more pronounced OS advantage in terms of improving OS (Table 2). In indirect comparative analyses, tor + che showed a superior OS benefit compared to ate + bev + che, ate + che, dur + che, niv + che, niv + ipi, niv + ipi + che, pem + ipi, pembrolizumab and nivolumab (Supplementary Table 7). In the direct comparative analysis of PFS in patients with nonsquamous NSCLC (Supplementary Figure 5), all experimental regimens except nivolumab had significantly better PFS outcomes than chemotherapy, with sin + che providing the greatest PFS benefit (HR = 0.48; 95% CI, 0.36–0.64). Nivolumab had worse PFS compared to other regimens in an indirect comparative analysis (Supplementary Table 8).

**TABLE 2:** Rankings based on overall survival and progression-free survival.

		1st	2nd	3rd	4th	5th
Total population	OS	pen + che	cem	sin + che	tis + che	cam + che
	PFS	pen + che	cam + che	sug + che	tis + che	tor + che
Nsq-NSCLC	OS	tor + che	pem + che	ate	cem + che	cem
	PFS	sin + che	pem + che	cem + che	pem	sug + che
Sq-NSCLC	OS	cem	cam + che	pen + che	sug + che	tis + che
	PFS	sug + che	cam + che	pem	pem + ipi	pen + che
PD-L1 ≥ 50% cohort	OS	cam + che	cem	cem + che	sug + che	ate + bev + che
	PFS	cam + che	sin + che	pem + che	tis + che	sug + che
PD-L1 1%–49% cohort	OS	cem + che	cam + che	pem + che	niv + ipi + che	tor + che
	PFS	cam + che	cem + che	tis + che	sug + che	tor + che
PD-L1 < 1% cohort	OS	niv + ipi	cam + che	niv + ipi + che	sug + che	pem + che
	PFS	ser + che	tor + che	cam + che	sug + che	sin + che

OS, overall survival; PFS, progression-free survival; pen, penpulimab; cem, cemiplimab; sin, sintilimab; tis, tislelizumab; cam, camrelizumab; sug, sugemalimab; tor, toripalimab; pem, pembrolizumab; ate, atezolizumab; sug, sugemalimab; tis, tislelizumab; ipi, ipilimumab; niv, nivolumab; ser, serplulimab; bev, bevacizumab; che, chemotherapy.

##### 3.2.5.2 Squamous NSCLC

20 trials evaluating 21 treatment options were included in the subgroup analysis of patients with squamous NSCLC. Direct comparisons demonstrated there were 14 experimental treatment arms, including cemiplimab (HR = 0.48; 95%CI: 0.30–0.77), cam + che (HR = 0.55; 95%CI: 0.40–0.75), pen + che (HR = 0.55; 95%CI: 0.40–0.75), sug + che (HR = 0.56; 95%CI: 0.38–0.82), tis + che (HR = 0.58; 95%CI: 0.41–0.81), sin + che (HR = 0.57; 95%CI: 0.35–0.91), cem + che (HR = 0.61; 95%CI: 0.42–0.88), niv + ipi (HR = 0.63; 95%CI: 0.50–0.80) niv + ipi + che (HR = 0.63; 95%CI: 0.47–0.85), pem + che (HR = 0.71; 95%CI: 0.59–0.85), ser + che (HR = 0.73; 95%CI: 0.58–0.92), pembrolizumab (HR = 0.76; 95%CI: 0.63–0.91), niv + che (HR = 0.76; 95%CI: 0.58–0.99), and nivolumab (HR = 0.79; 95%CI: 0.62–0.99), exhibited superior outcomes in terms of OS compared to chemotherapy (Supplementary Figure 6). Based on indirect comparisons, ate + che had an increased risk of death compared to cam + che, pen + che, cemiplimab, cem + che, sug + che, tis + che, niv + ipi, and niv + ipi + che (Supplementary Table 9). Based on the direct comparisons for PFS, PD-1/PD-L1 inhibitors regimens, except nivolumab (HR = 0.83; 95%CI: 0.54–1.27) and dur + tre + che (HR = 0.77; 95% CI: 0.58–1.02), had significantly better PFS outcomes than chemotherapy (Supplementary Figure 7). However, based on indirect comparative analyses, sug + che was most likely to show a statistically significant PFS benefit compared to cem + che, sin + che, niv + ipi + che, pem + che, dur + che, dur + tre + che, ser + che, ate + che and nivolumab (Supplementary Table 10).

#### 3.2.6 PD-L1 expression cohorts

In unifying the grouping criteria for PD-L1 expression levels, most studies tend to use the tumour proportion score (TPS) as the basis for delineation, whereas some immunohistochemical diagnostic techniques take into account PD-L1 expression in tumour cells (TCs) and tumour-infiltrating immune cells (ICs). To achieve a more consistent grouping system, we set the following rules: “TPS ≥ 50%” and “TC ≥ 50% or IC ≥ 10%” were analyzed as PD-L1 ≥ 50%; “TPS < 1%” and “TC < 1% or IC < 1%” as PD-L1 < 1%; and “1 ≤ TPS ≤ 49%” and “1% ≤ TC ≤ 49% or 1% ≤ IC < 10%” as 1% ≤ PD-L1 ≤ 49% (Tsao et al., 2018; Herbst et al., 2020).

##### 3.2.6.1 PD-L1 ≥50%

The OS for the cohort with PD-L1 expression ≥50% was derived from 22 trials evaluating 27 experimental treatment regimens. Results from the NMA show that all PD-1/PD-L1 inhibitors regimens, except atezolizumab, ate + bev + che, cam + che, cem + che, durvalumab, dur + tre, niv + che, niv + bev + che and tor + che were significantly better OS than chemotherapy (Supplementary Figure 8). Cam + che exhibited the most prominent OS advantage over chemotherapy, with a hazard ratio of 0.48 (95% CI: 0.21–1.11), and had the highest likelihood of achieving a superior ranking among the treatment options (Table 2). The PFS analysis for patients exhibiting a PD-L1 expression ≥50% was derived from comprehensive data encompassing 21 clinical trials, which assessed a diverse range of 17 distinct treatment modalities. Direct comparisons indicate that all PD-1/PD-L1 inhibitor regimens demonstrated superior PFS outcomes compared to chemotherapy (Supplementary Figure 9). A direct comparison revealed that cam + che was most likely to show a statistically significant PFS benefit compared to niv + ipi, pem + ipi, pembrolizumab, atezolizumab and nivolumab (Supplementary Table 12).

##### 3.2.6.2 1% ≤ PD-L1 ≤ 49%

The OS for the cohort with PD-L1 1%–49% was based on 13 trials with 11 treatment regimens. Direct comparisons demonstrated that among PD-1/PD-L1 inhibitor regimens, only cem + che (HR = 0.50; 95%CI: 0.32–0.79), cam + che (HR = 0.52; 95%CI: 0.27–1.00), pem + che (HR = 0.63; 95%CI: 0.50–0.79) and niv + ipi + che (HR = 0.70; 95%CI: 0.52–0.94) exhibited superior efficacy compared to chemotherapy alone. Compared with chemotherapy, no statistically significant difference in survival among atezolizumab monotherapy, ate + che, niv + ipi, sug + che, pembrolizumab monotherapy and tor + che (Supplementary Figure 10). Indirect estimations indicated that no statistically discernible difference in survival outcomes among cem + che, cam + che, pem + che, niv + ipi + che, sug + che and tor + che (Supplementary Table 13). Cem + che was identified as the most effective treatment, as evidenced by its P-score of 0.8938. The preliminary findings of the subgroup analysis of PFS for patients with PD-L1 1%–49% are based on the results of 17 clinical trials which evaluated 12 different treatment options. Results from direct comparisons shows all experimental treatment arms had statistically better PFS outcomes compared chemotherapy (Supplementary Figure 11). Upon examination of indirect comparisons, no significant differences in PFS among cem + che, cam + che, tis + che, tor + che, sug + che, pem + che and sin + che (Supplementary Table 14). Cam + che ranked as the best treatment (P-score = 0.8540).

##### 3.2.6.3 PD-L1 <1%

The subgroup analysis of OS for low PD-L1 expression is based on 17 trials evaluating 18 treatment options (Supplementary Figure 12). According to the results of direct comparisons, there were 7 experimental treatment arms, including ate + che, cam + che, niv + che, niv + ipi, niv + ipi + che, sug + che and pem + che, had better OS outcomes than chemotherapy. Compared to chemotherapy, niv + ipi had the highest probability of being deemed the optimal treatment option, with a hazard ratio of 0.64 (95% CI: 0.51–0.81), while cam + che ranked second (HR = 0.62; 95%CI: 0.41–0.91). In summary, the statistically significant difference among PD-1/PD-L1 inhibitors in indirect comparisons was the higher mortality risk associated with durvalumab and dur + che, when compared to pem + che, niv + ipi, niv + ipi + che treatments. (Supplementary Table 15). In a subgroup analysis of PFS for patients with PD-L1 expression <1%, we combined data from 17 studies and evaluated 13 different treatment options in depth. Direct comparisons between showed that PD-1/PD-L1 inhibitors regimens, except cem + che (HR = 0.73; 95%CI: 0.50–1.07), had significantly better PFS outcomes than chemotherapy (Supplementary Figure 13). A pairwise comparison revealed that ser + che showed a statistically significant PFS benefit compared to niv + ipi, niv + che and ate + che (Supplementary Table S16).

## 4 Discussion

PD-1/PD-L1 inhibitors have been widely recognised as the standard treatment of patients with NSCL without driver mutations in the first-line setting (Riely et al., 2024). Therefore, we conducted this network meta-analysis to analyse the efficacy and safety of different PD-1 and PD-L1 inhibitors.

The PD-1/PD-L1 in combination with chemotherapy or a CTLA-4 antibody might show a greater advantage in improving OS than PD-1/PD-L1 monotherapy. Pen + che ranked first in OS, followed by cemiplimab and sin + che. Most two-drug combination regimens or three-drug combination regimens have better PFS outcomes. Pen + che regimen had the greatest benefits in PFS, followed by cam + che, and sug + che. In contrast with the therapeutic efficacy, the majority of two-drug or three-drug combination regimens have been found to increase the risk of ≥3 TRAE. In this context, monotherapy demonstrates its distinctive advantages, with cemiplimab monotherapy, in particular, exhibiting a relatively low profile of adverse reactions, thereby constituting a prudent option for patients with poor physical conditions. Given the significant increase in immune–related AE in ICI ± chemotherap, the benefit of combination therapies should be weighed against the considerably higher risk of adverse events.

Our NMA provides a preferred ranking probability for each treatment to determine which treatment option ranks best among all of our options and in specific subgroups. Pen + che, cam + che, tis + che and sin + che were more effective than other regimens for both PFS and OS in the NMA of all patients. Different results were seen when subgroup analyses of treatment regimens were performed for patients grouped by PD-L1 expression level. In the subgroup of patients with PD-L1 ≥ 50%, cam + che had the highest probability of being the best treatment regimen for OS in the first-line setting. Our findings are not quite the same as those of He et al. (He et al., 2022), which found cemiplimab to be the best first-line therapy in cohorts with PD-L1 ≥ 50%, although they have included CameL (cam + che versus che). Given that our study not only covered CameL but also additionally included CameL-sq (cam + che versus che), and in particular that the latter may have a greater impact on the results of the NMA of PD-L1 expression ≥50%, our final conclusions differ from those of the previous study by He et al. Importantly, our results include more recent data and more trials than the previous NMA, including CheckMate 9LA (niv + ipi + che versus chemotherapy), EMPOWER-Lung 1 (cemiplimab versus chemotherapy), EMPOWER-Lung 3 (cem + che versus che) and IMpower150 (ate + bev + che versus bev + che). However, we did not find any statistically significant differences in OS. Based on these previous findings, we believe that cam + che may become the first-line treatment of choice in patients with PD-L1 ≥ 50%. However, this should be interpreted with caution until final OS results from other studies are published. And because cemiplimab, cem + che, sug + che and pem + che show comparable efficacies and have being studied with more patients, these agents can still be used as first-line therapy for this patient group. Given the significant increase in AEs with ICI/chemotherapy, the benefits of combination therapy should be weighed against the significantly increased risk of AEs.

In the cohort of patients with intermediate PD-L1 expression (1%–49%), the cem + che combination outperformed all other ICIs in terms of OS and PFS, making it the therapy of choice in this cohort. Fewer studies have been conducted for PD-L1 tumor proportion score 1%–49%. Our results differ from those of Fukuda et al. (2021) and Fukuda et al. (2022) whose study of PD-L1 expression 1%–49% in squamous and non-squamous NSCLC showed that pem + che yielded the best OS results, followed by niv + ipi + che. However, their network meta-study did not include a series of recently published studies such as EMPOWER-Lung 3 and their study population included patients with early-stage NSCLC.

Choosing immunotherapeutic agents for patients with advanced lung cancer with PD-L1 < 1% has been challenging, and our results suggest that the niv + ipi had the highest likelihood of benefit in terms of OS. While mature results in OS have yet to emerge for the SER + CHE regimen due to time constraints, its notable benefit in PFS has offered new optimism for this patient population and also suggests considerable potential for future developments. Given the lack of a statistically significant difference between niv + ipi, pem + che, and niv + ipi + che in terms of OS in the pairwise comparison, as well as considering the time-to-market of the drugs, their wide clinical utility, and patient economic factors, it is our recommendation that pem + che be considered the preferred first-line regimen for patients with PD-L1 < 1%. Peng et al.‘s (Peng et al., 2021) network meta-analysis found pem + che more effective for EGFR/ALK wild-type patients with PD-L1 < 1%, aligning with our findings for low PD-L1 expression. The NCCN guidelines do not recommend the use of PD1/PD-L1 inhibitors in patients with low PD-L1 expression, but the results of the ASTRUM-004, CameL, CameL-sq and GEMSTONE-302 might offer new hope for this population.

Squamous NSCLCs is a much more complex form of disease, most often seen in older men, strongly associated with smoking, had an extremely high rate of genetic mutations, but is less sensitive to radiotherapy and chemotherapy (Socinski et al., 2018). In comparison, nonsquamous NSCLC is less complex, often caused by single driver mutations, and has a better response to chemotherapy. We re-evaluated efficacy according to histologic type, comparing PD-1/PD-L1 inhibitors in squamous and non-squamous cohorts. In patients with non-small cell lung cancer (NSCLC), specific regimens, including cemiplimab, cem + che, sin + che, tis + che and pem + che have demonstrated efficacy in both squamous and non-squamous patient populations. However, regimens such as tor + che or ate + che have been observed to be more effective in non-squamous patients. Our NMA results demonstrated that cemiplimab had the highest likelihood of benefit in terms of OS in squamous NSCLC, whereas in non-squamous NSCLC, tor + che had the highest likelihood of benefit in terms in OS.

This network meta-analysis provides a broader review of advanced NSCLC treatment, not only comparing the efficacy and safety of different treatment regimens in advanced NSCLC (Wang et al., 2022), but also providing in-depth analyses based on the most recent data (e.g. results from long-term follow-up studies such as CHOICE-01, EMPOWER Lung-3 and Keynote-189). In the absence of directly comparable clinical trials, this study provides clinicians with a valuable decision-making tool, especially when choosing between several potentially effective treatment options. In addition, we performed a subgroup analysis based on PD-L1 tumor proportion score and histological type of NSCLC to further assess the robustness of the results.

Finally, there are some limitations to this study. (1) Only phase 3 clinical trials were included in our study, but efficacy data from some of the studies have been updated based on prior published conference abstracts. (2) Despite the existence of numerous commercial PD-L1 assays, there is considerable variation in their analytical performance. A previous review (Doroshow et al., 2021) demonstrated that 28-8, 22C3 and SP263 assays have comparable performance with respect to tumor cell staining based on TPS and regardless of intensity, whereas the SP142 assay had lower sensitivity. In clinical applications, the first three methods mentioned above focus on tumor cell PD-L1 expression, while SP142 evaluates both tumor cells and tumor-infiltrating immune cells. The IMpower study suggests that there may be misclassification of patients based on tumor cell PD-L1 expression alone. (3) In terms of efficacy, we assess OS, PFS, and ORR, but when recommending preferred therapies, we focus more on OS outcomes. Some studies have pre-empted the publication of interim PFS data (e.g., RATIONALE 304 or RATIONALE 307) to give a new perspective on the NSCLC patients using PD-1/PD-L1 inhibitors, and their immature OS data may have some impact on the results of our network meta-analysis. (4) In addition, our study may have underestimated the benefit of the intervention regimen because some clinical trials often allowed patients to cross-over as their disease progressed.

## 5 Conclusion

Overall, pen + che, cemiplimab, sin + che, tis + che and cam + che were the most effective treatments for all the patients. The main treatments varied when patients were grouped according to different characteristics. In terms of OS, we believe that the preferred treatment regimens for patients with PD-L1 ≥ 50%, PD-L1 1%–49% and PD-L1 < 1% are cam + che, cam + che and niv + ipi, respectively. A subgroup analyses by tumour histology, we found that tor + che performed best in non-squamous cancers, while cemiplimab alone was the treatment of choice in squamous cancers. It can be observed that the recently introduced therapeutic agents appear to demonstrate enhanced efficacy. However, the outcomes of additional direct comparisons are more worthy of expectation.

## Data Availability

The original contributions presented in the study are included in the article/Supplementary Material, further inquiries can be directed to the corresponding authors.
