# Supplementary material for: A network comparison on efficacy and safety profiling of PD-1/PD-L1 inhibitors in first-line treatment of advanced non-small cell lung cancer
Source: Front Pharmacol. 2025 Jan 6;15:1516735. doi: 10.3389/fphar.2024.1516735 (PMC11743166; doi:10.3389/fphar.2024.1516735)
Supplement: Supplementary file 1 [file DataSheet1.docx]

Supplementary Material

# Supplementary Figures and Tables

## Supplementary Figures

**
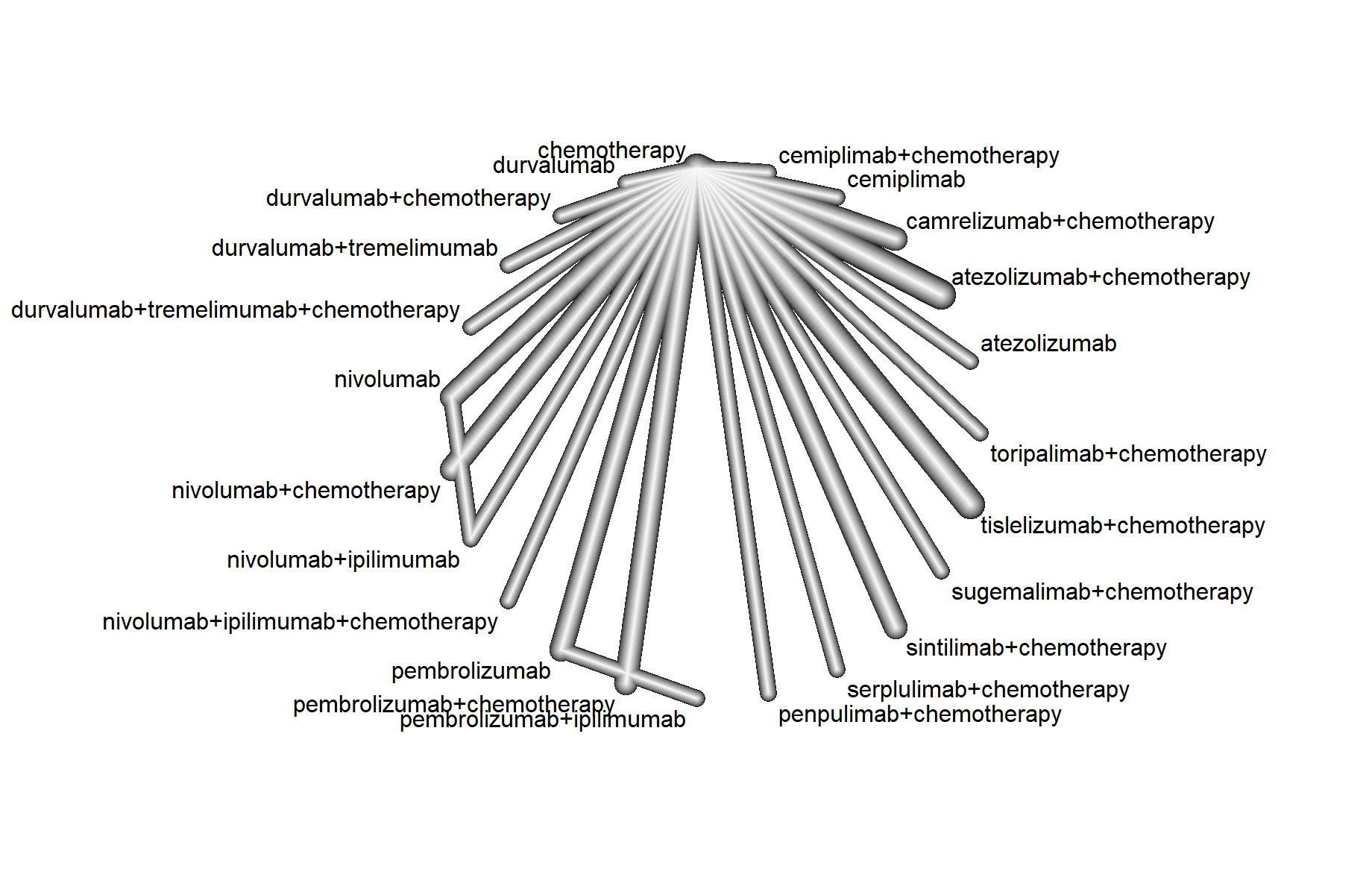
**

**Supplementary Figure 1.** Network diagram for the HR of progression-free survival. Separate model, Whole network level (I2 = 76.4%, Total; P< 0.0001, Within designs; P < 0.0001, Between designs; P =0.5816).

**
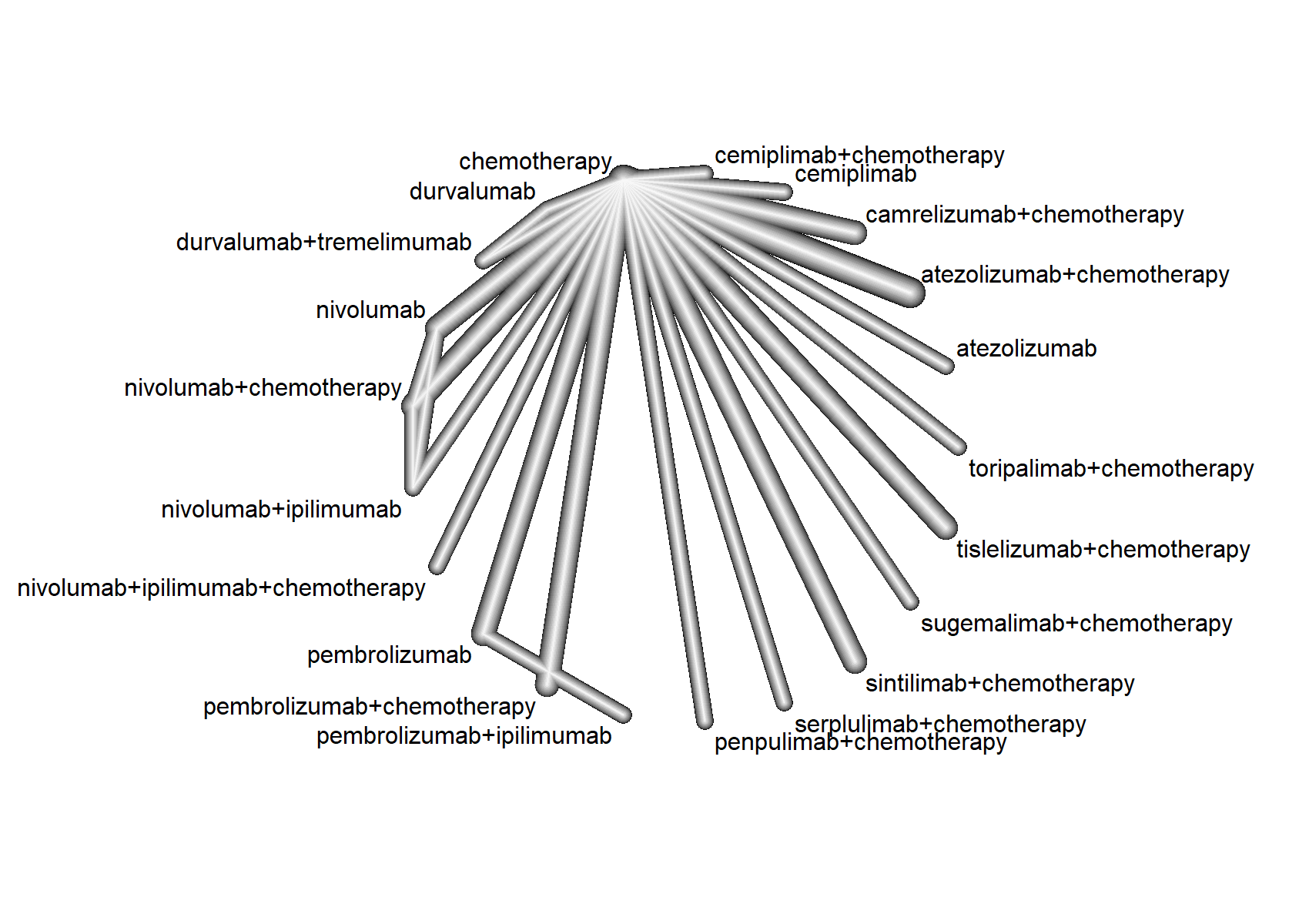
**

**Supplementary Figure 2.** Network diagram for the OR of objective response rate. Separate model, Whole network level (I2 = 54.3%, Total; P=0.02, Within designs; P =0.0577, Between designs; P=0.0492).

**
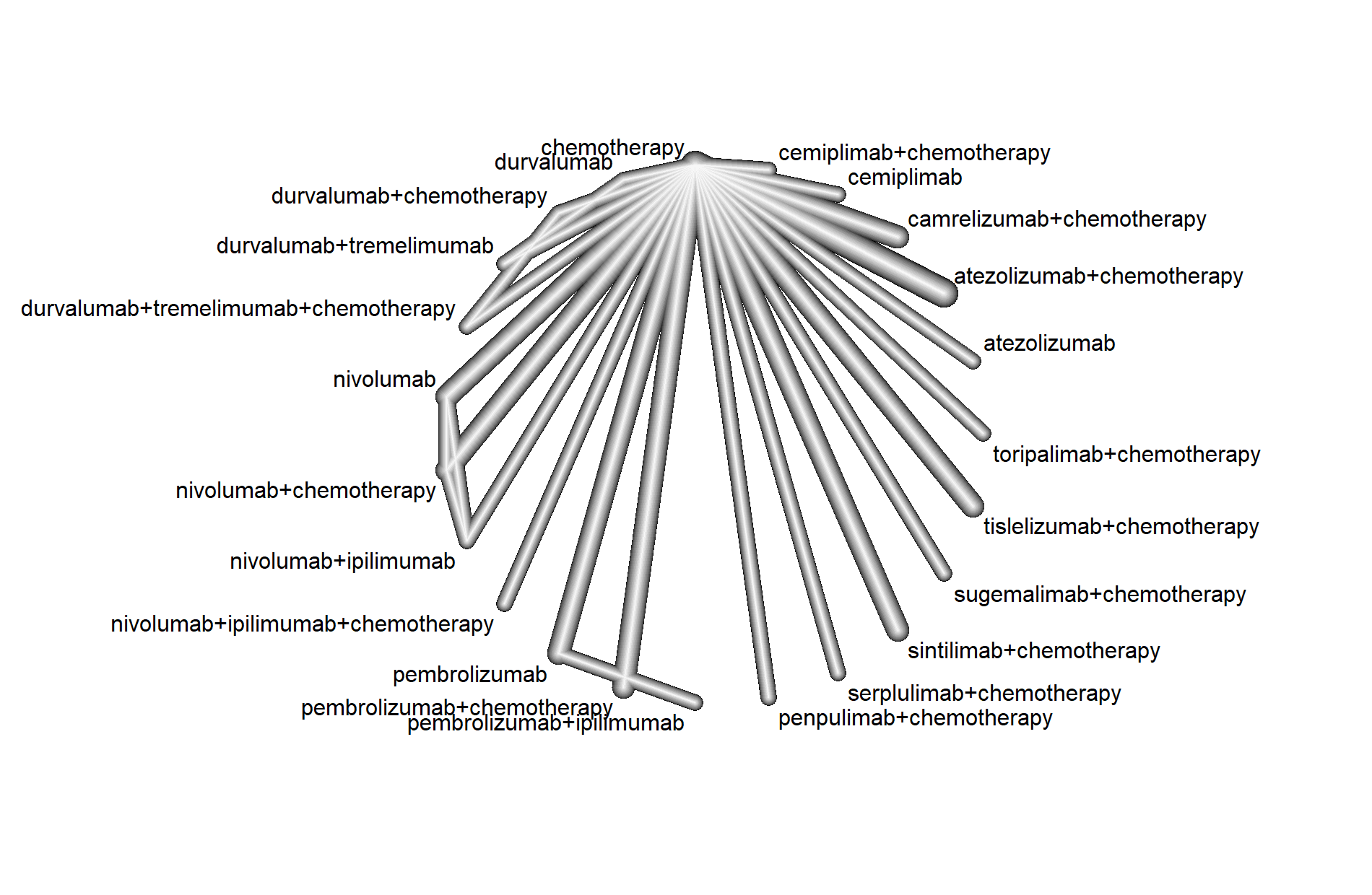
**

**Supplementary Figure 3.** Network diagram for the HR of treatment related adverse events (≥Grade 3). Separate model, Whole network level (I2 = 54.1%, Total; P=0.0204, Within designs; P =0.2232, Between designs; P=0.0061).

**
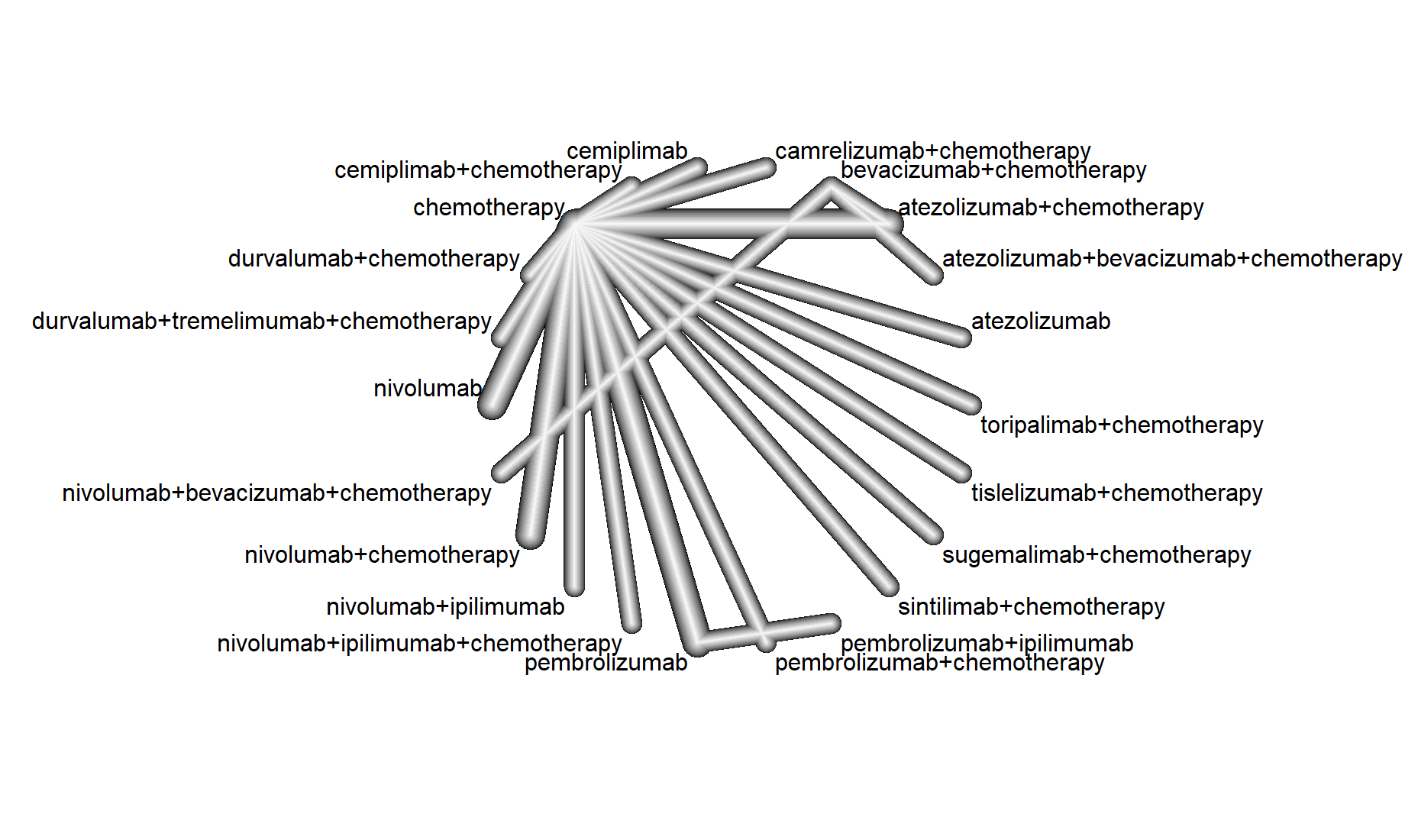
**

(A)

**
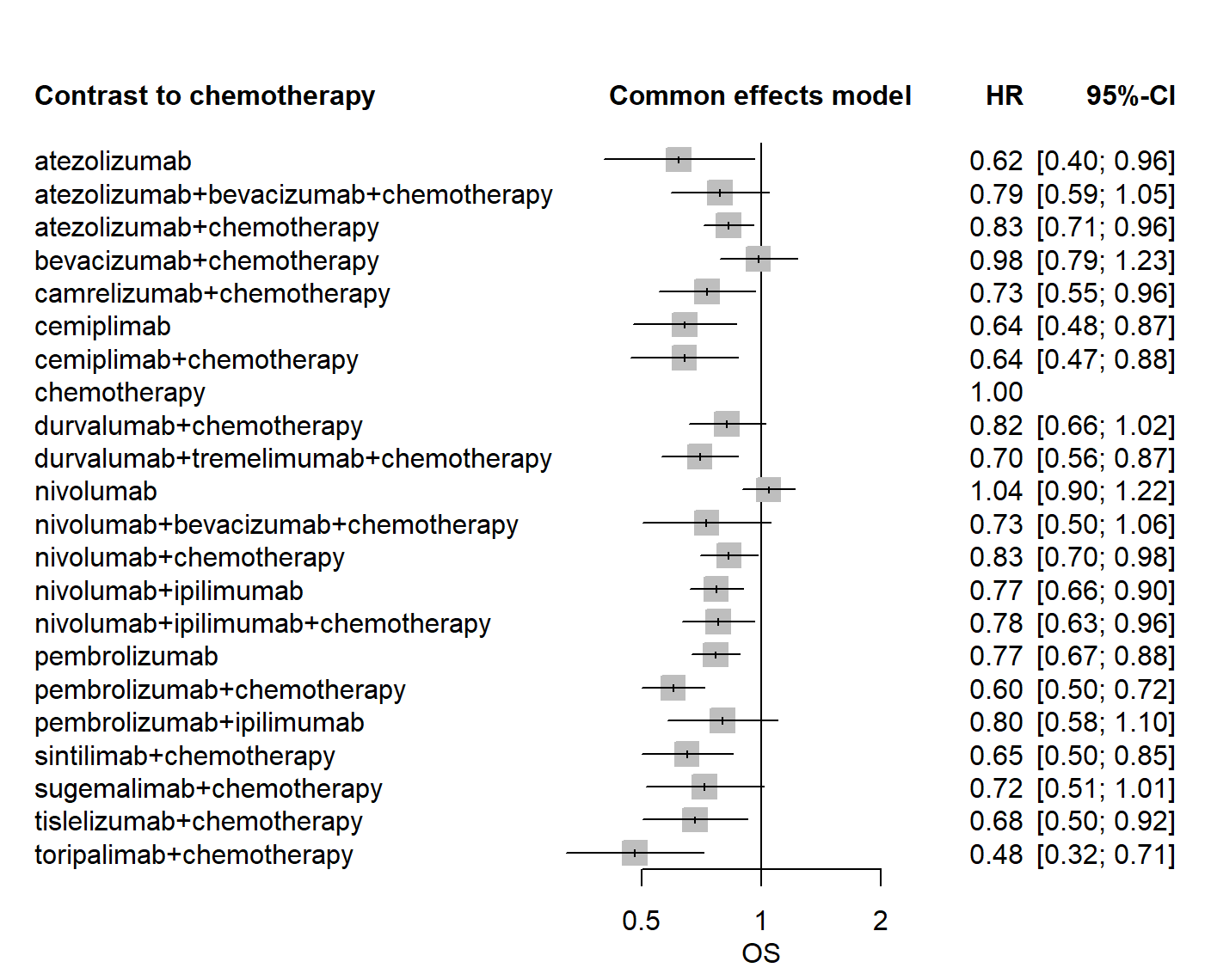
**

(B)

**Supplementary Figure 4.** Network diagram(A) and Forest plots(B) for the HR of overall survival for non-squamous NSCLC patients. Separate model, Whole network level (I2= 13.5%, Total; P= 0.3278, Within designs; P = 0.3278, Between designs; P=0)

**
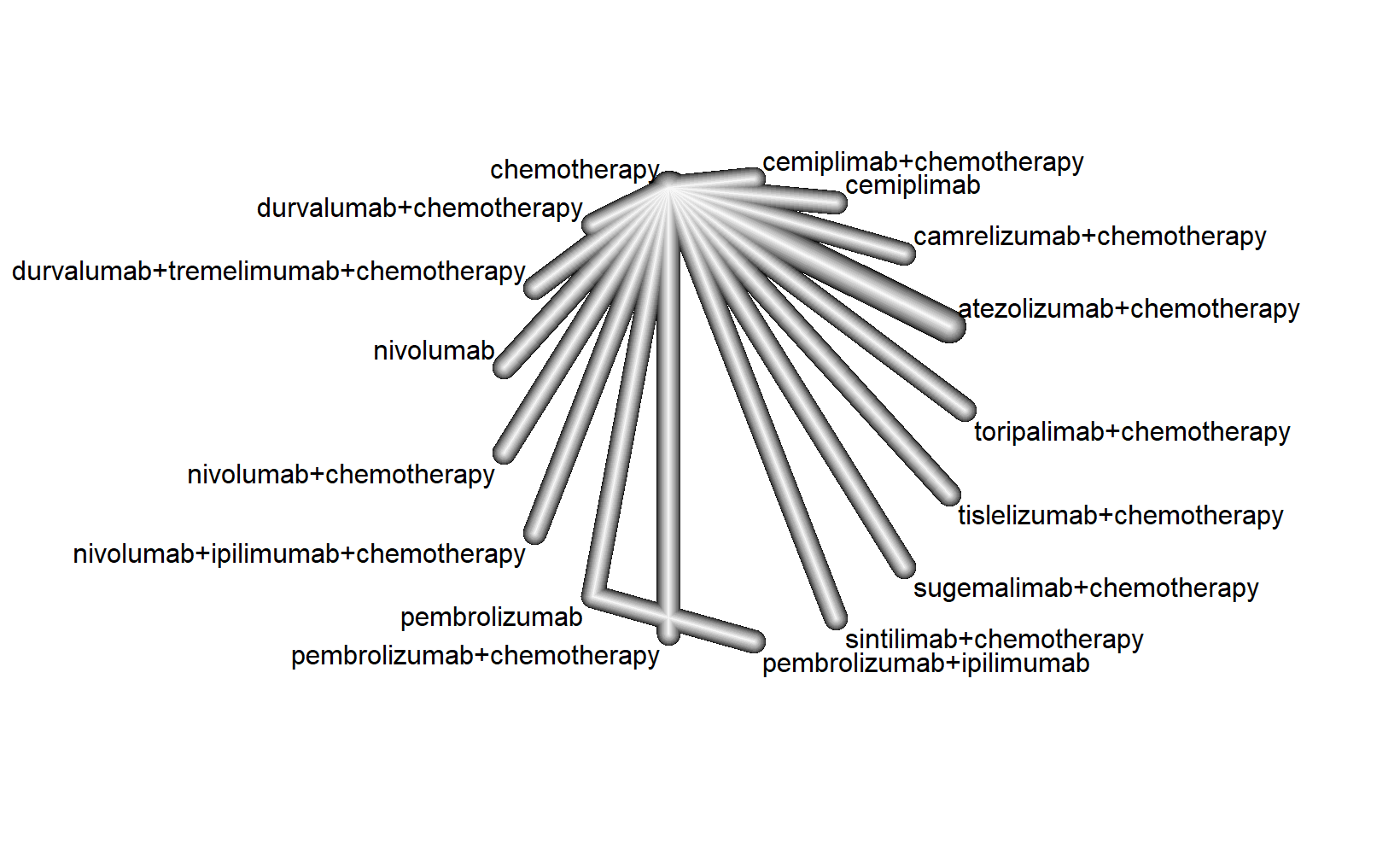
**

(A)

**
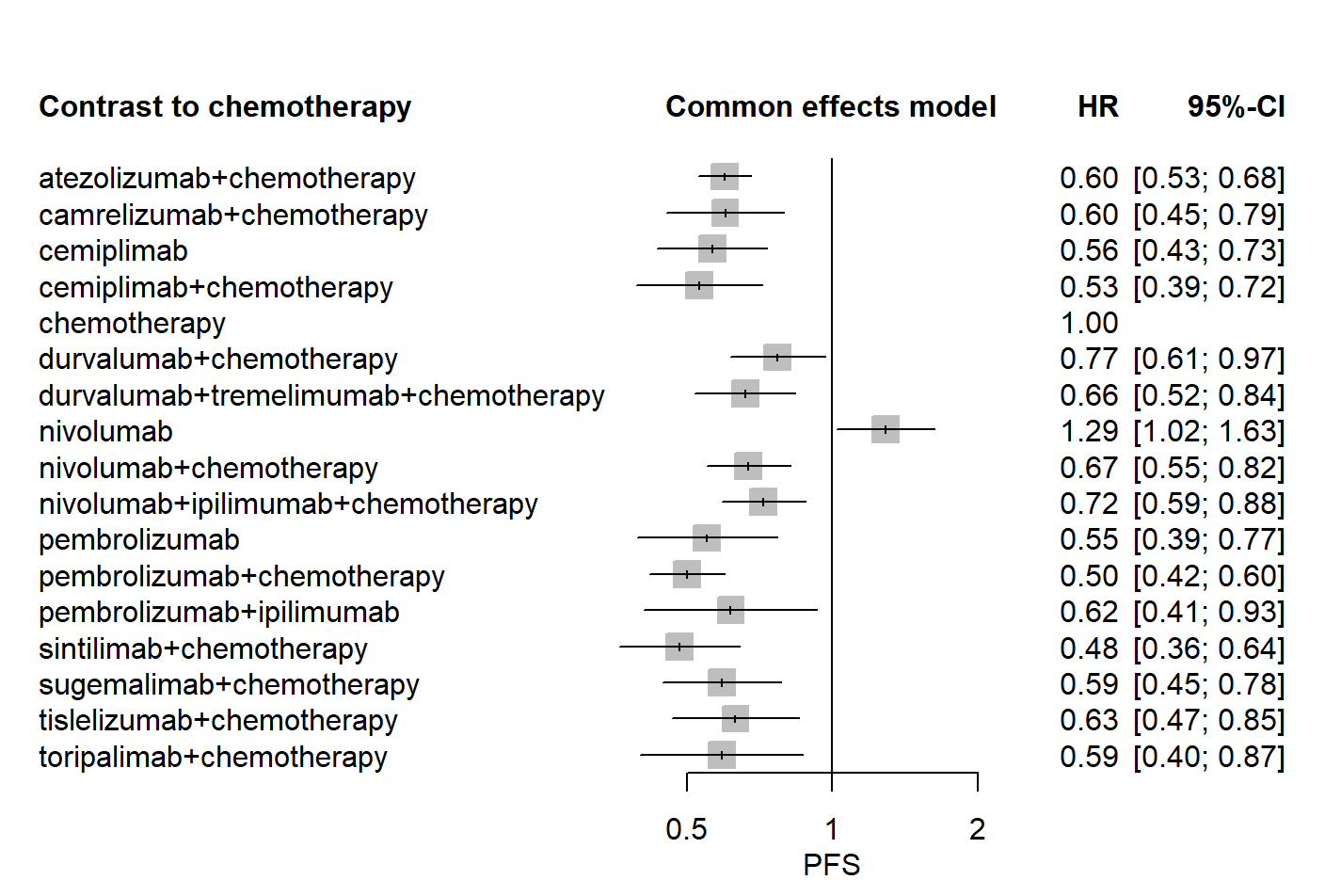
**

(B)

**Supplementary Figure 5.** Network diagram(A) and Forest plots(B) for the HR of progression-free survival for non-squamous NSCLC patients. Separate model, Whole network level (I2= 8.2%, Total; P= 0.2967, Within designs; P = 0.2967, Between designs; P=0)


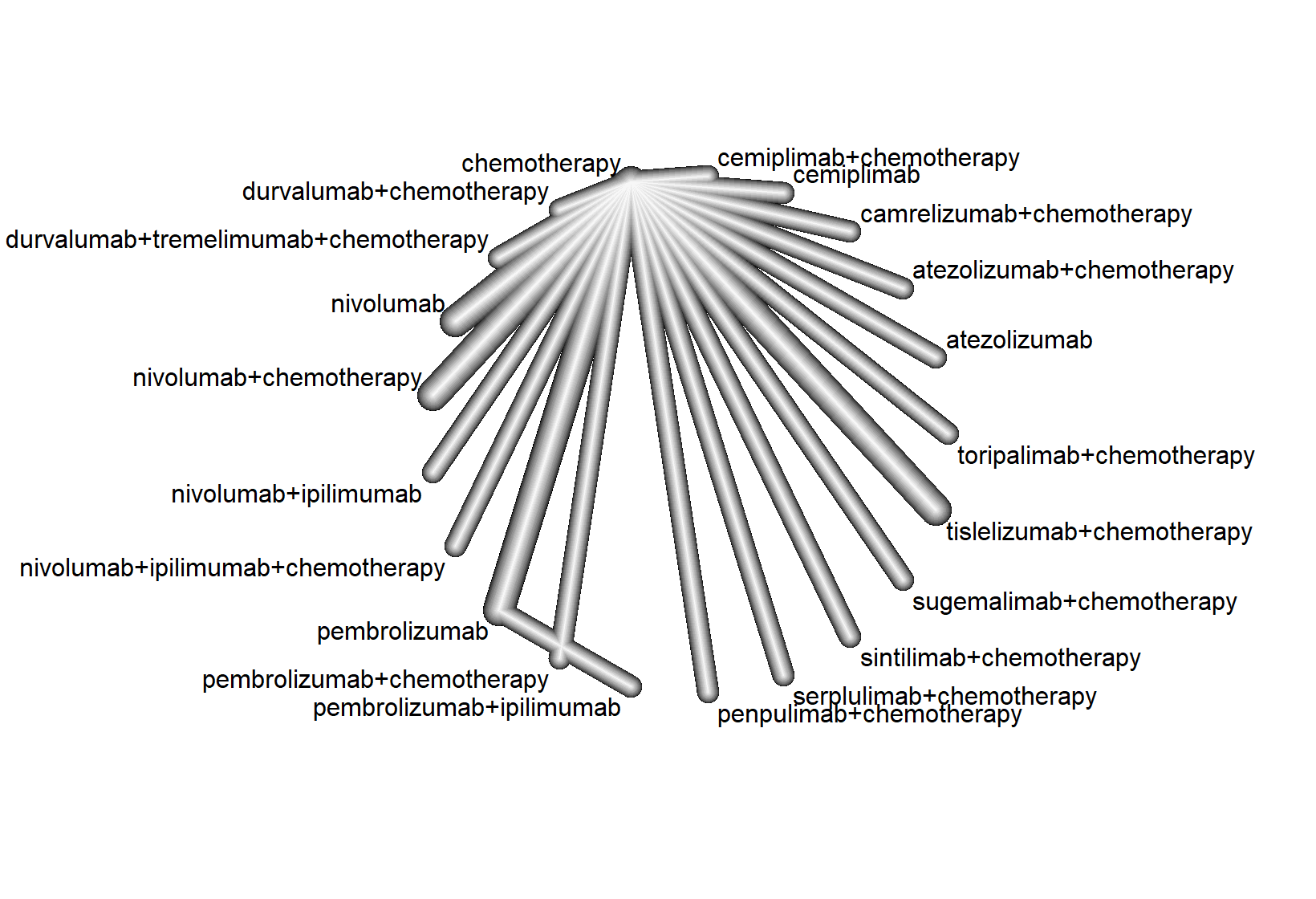


(A)

**
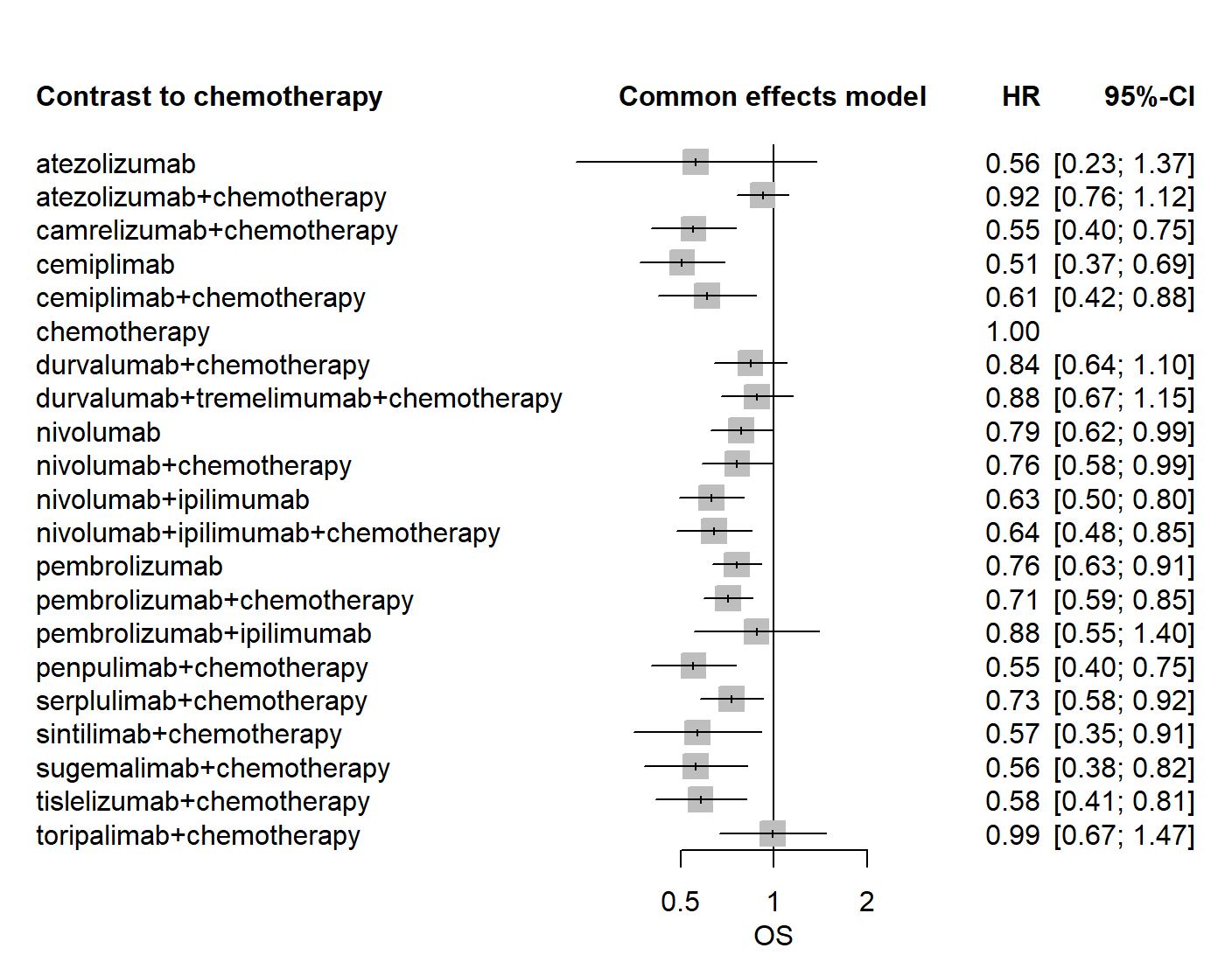
**

(B)

**Supplementary Figure 6.** Network diagram(A) and Forest plots(B) for the HR of overall survival for squamous NSCLC patients. Separate model, Whole network level (I2= 0%, Total; P= 0.8555, Within designs; P = 0.8555, Between designs; P=0)

**
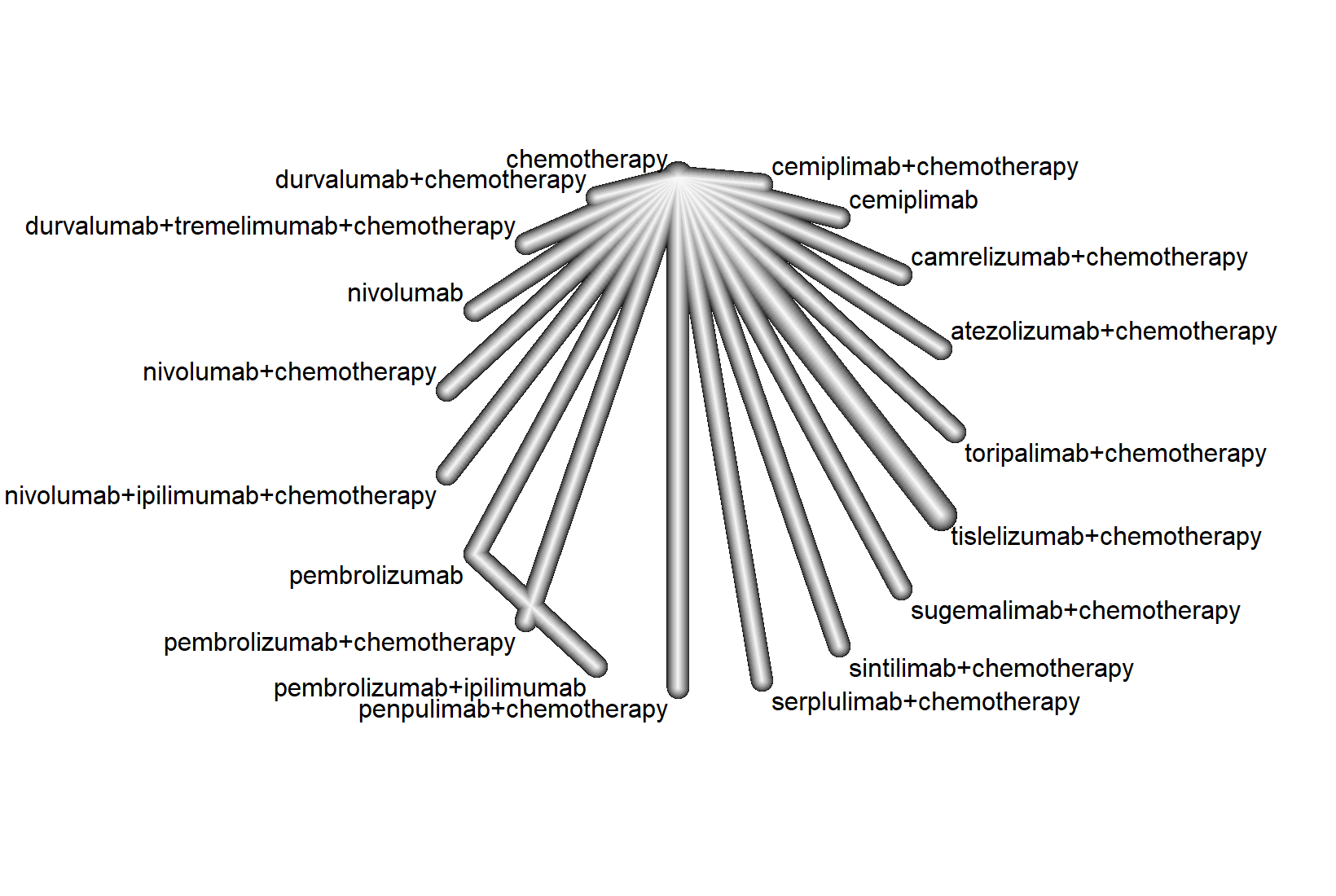
**

(A)

**
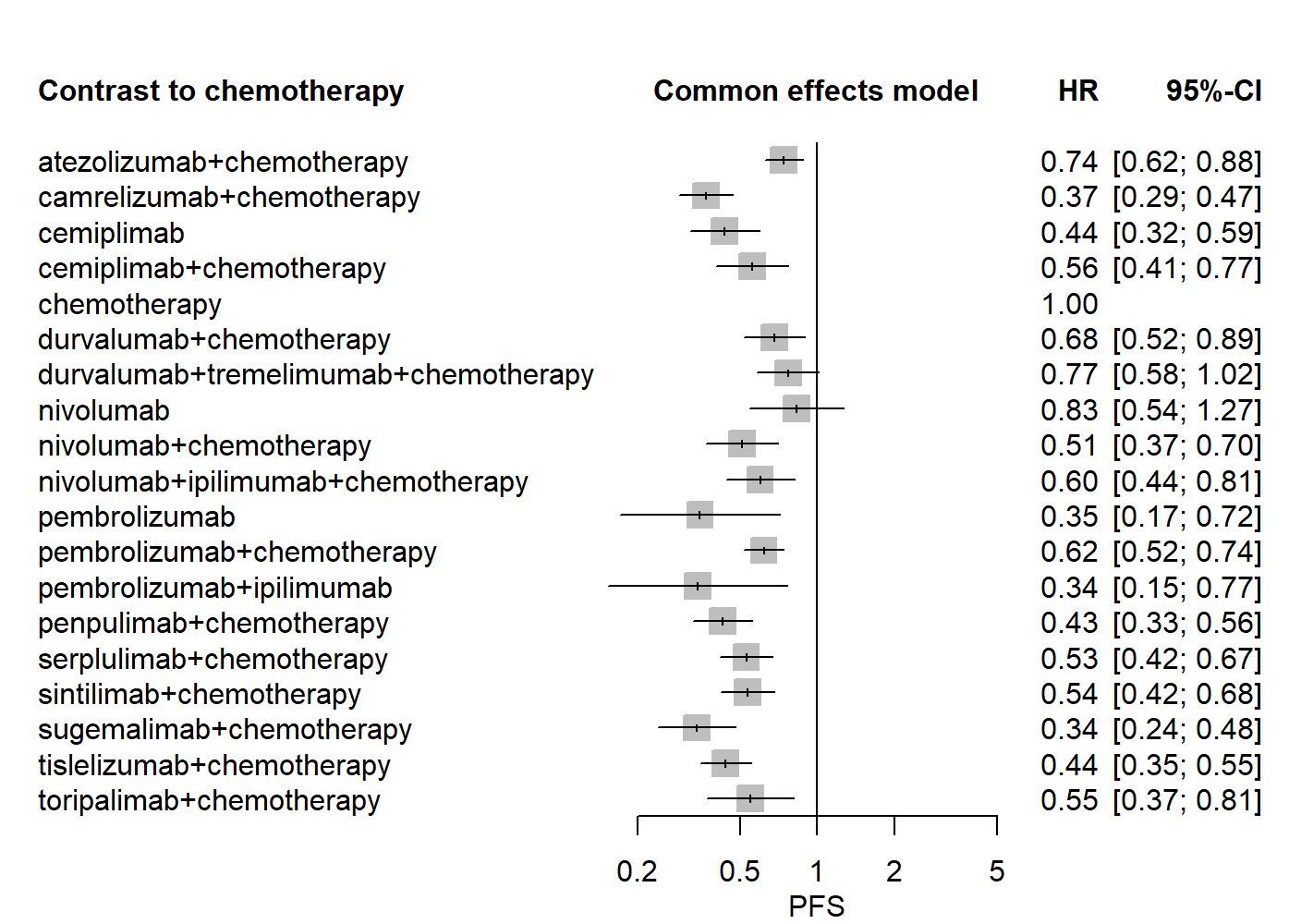
**

(B)

**Supplementary Figure 7.** Network diagram(A) and Forest plots(B) for the HR of progression-free survival for squamous NSCLC patients. Separate model, Whole network level (I2= 0%, Total; P= 0.8453, Within designs; P = 0.8453, Between designs; P=0)

**
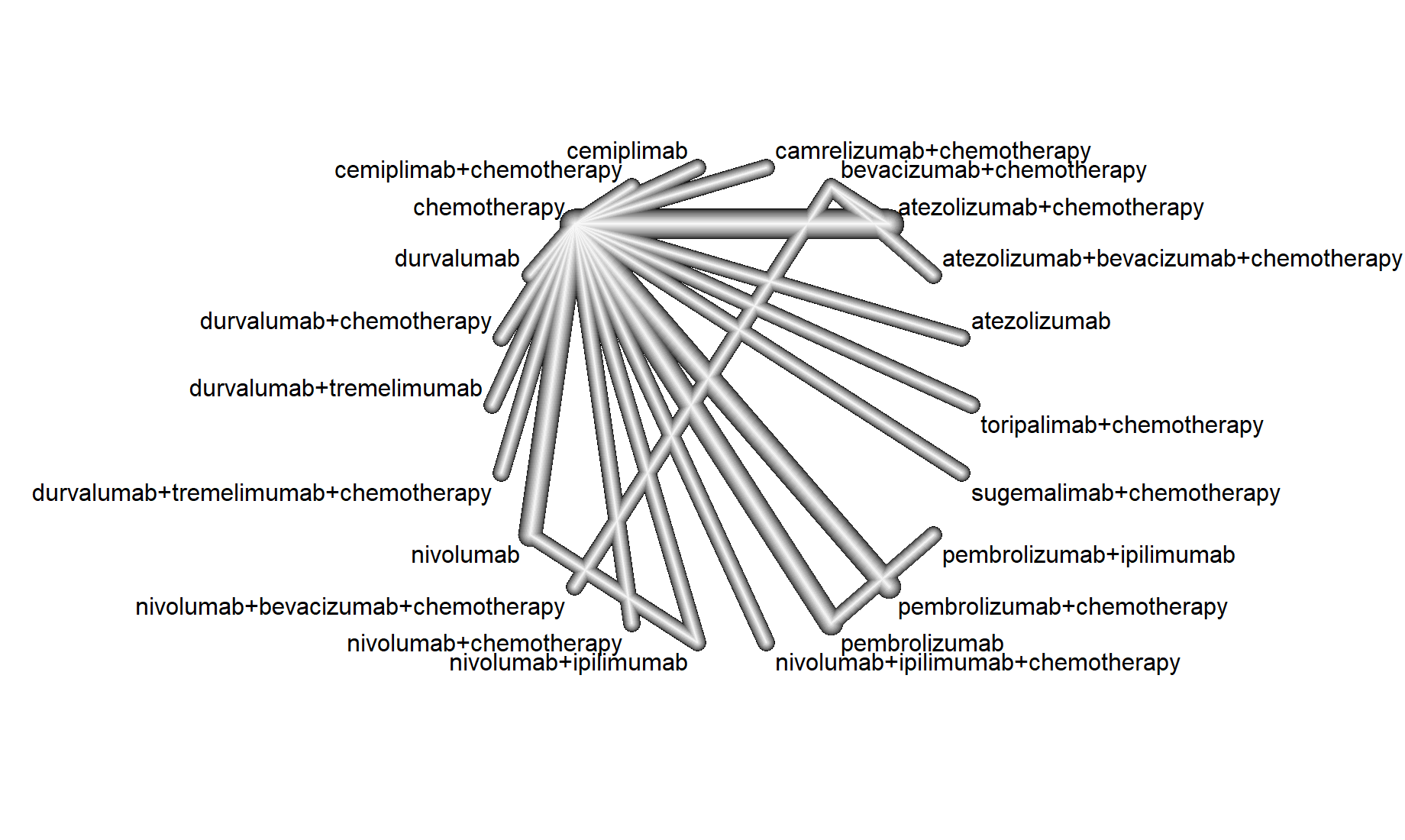
**

(A)

**
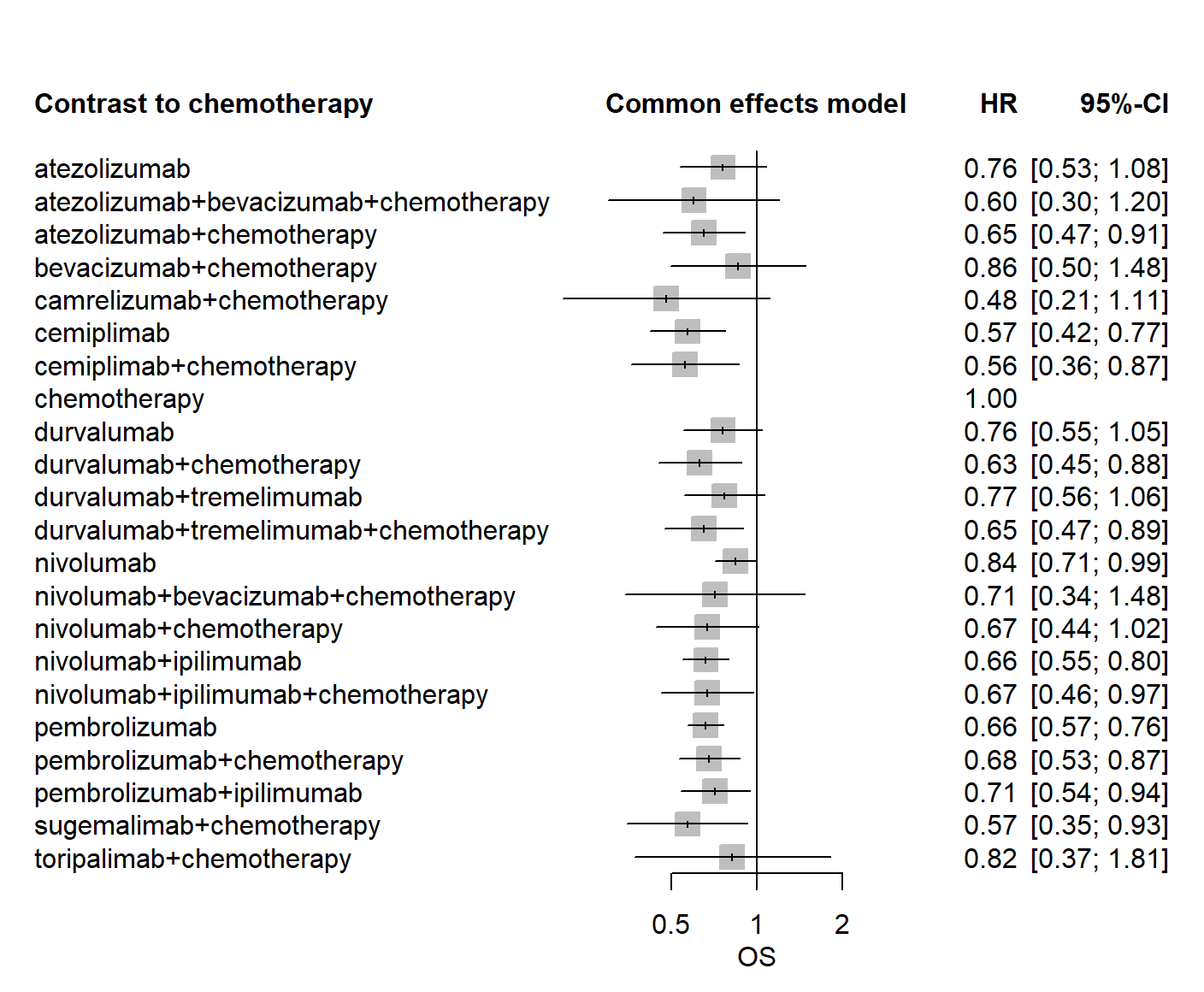
**

(B)

**Supplementary Figure 8.** Network diagram(A) and Forest plots(B) for the HR of overall survival for the PD-L1 ≥50%. Separate model, Whole network level (I2=0%, Total; P= 0.7115, Within designs; P = 0.5707, Between designs; P=0.9704).

**
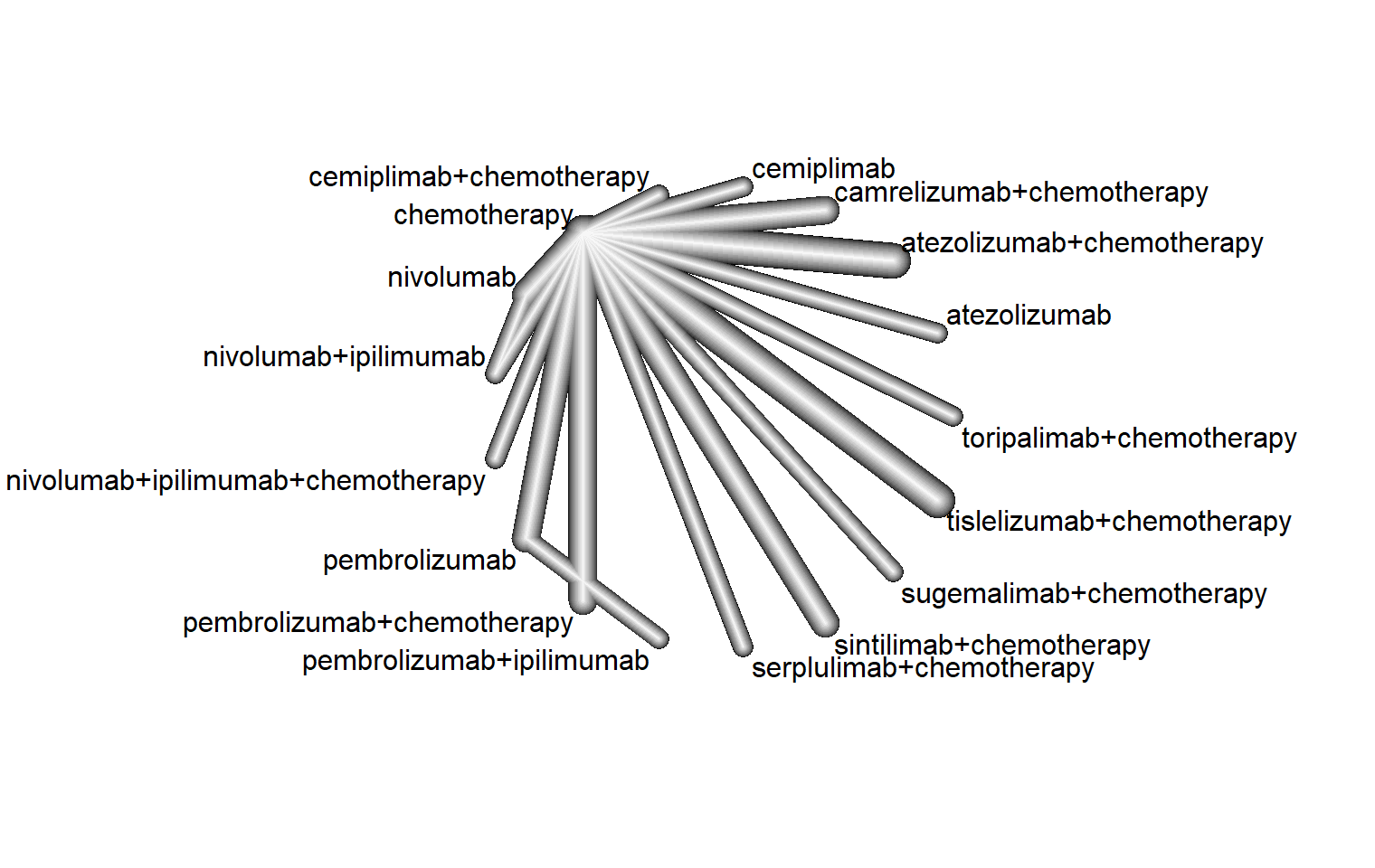
**

(A)

**
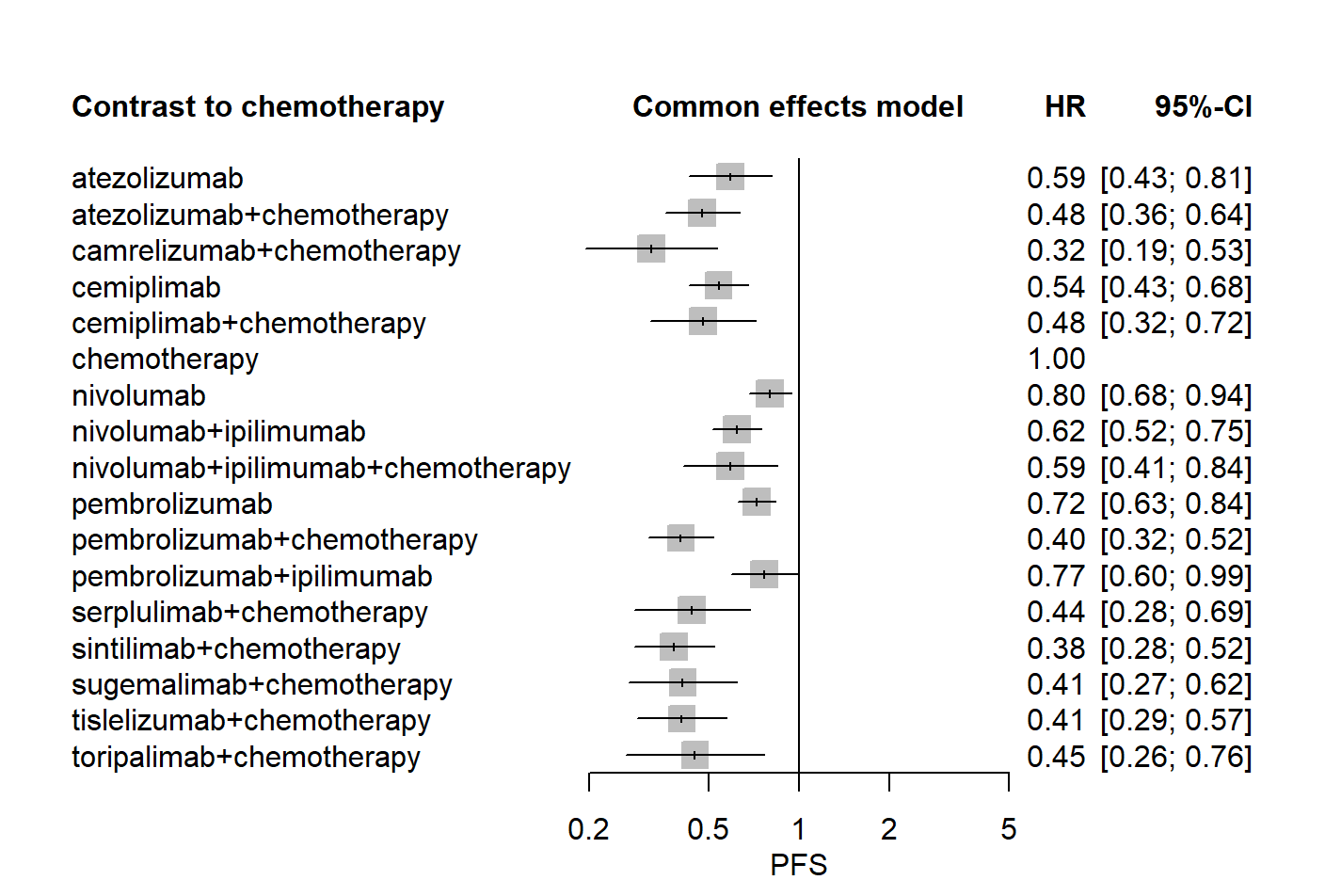
**

(B)

**Supplementary Figure 9.** Network diagram(A) and Forest plots(B) for the HR of progression-free survival for the PD-L1 ≥50%. Separate model, Whole network level (I2= 51.4%, Total; P= 0.0242, Within designs; P = 0.0157, Between designs; P= 0.6527)

**
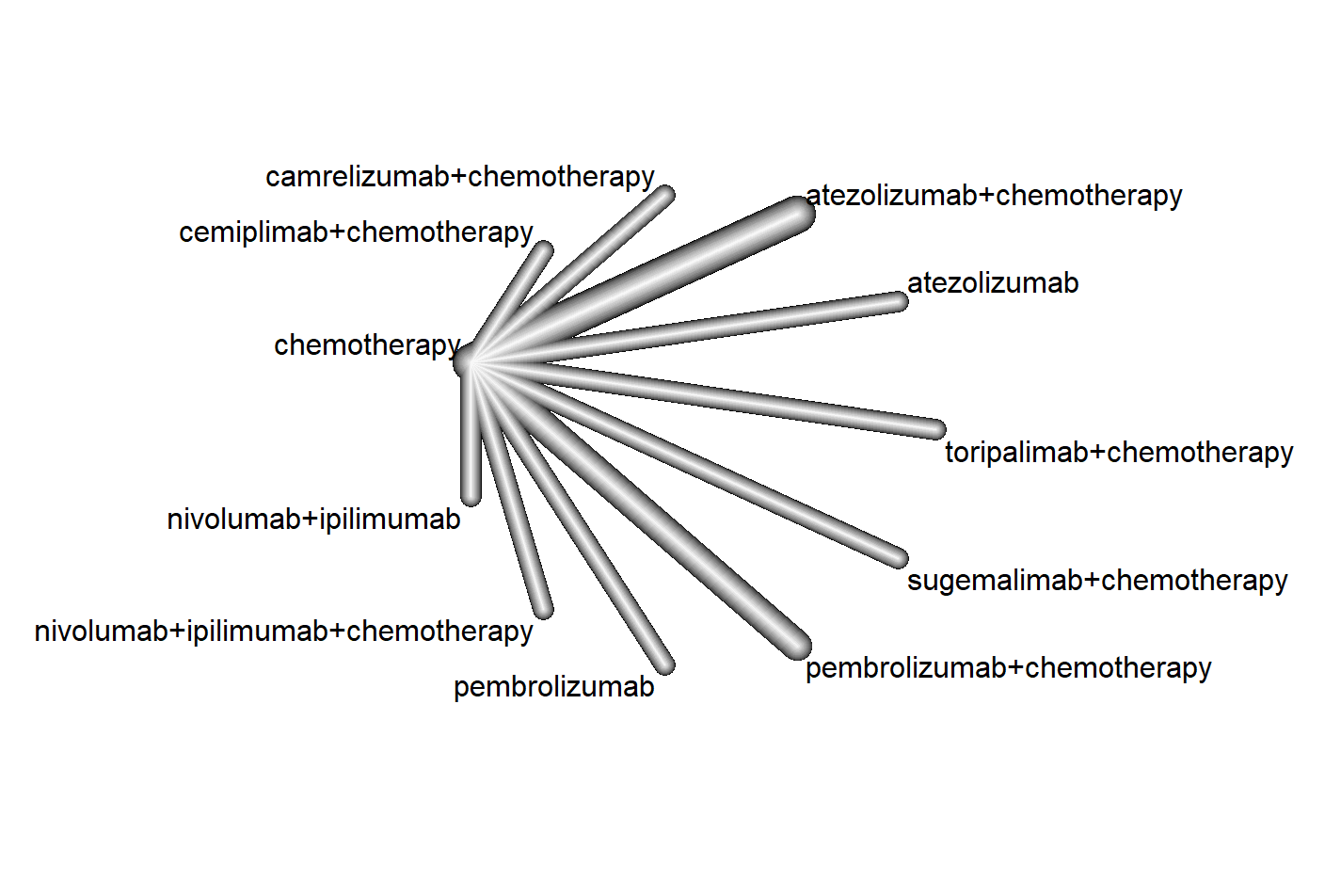
**

(A)

**
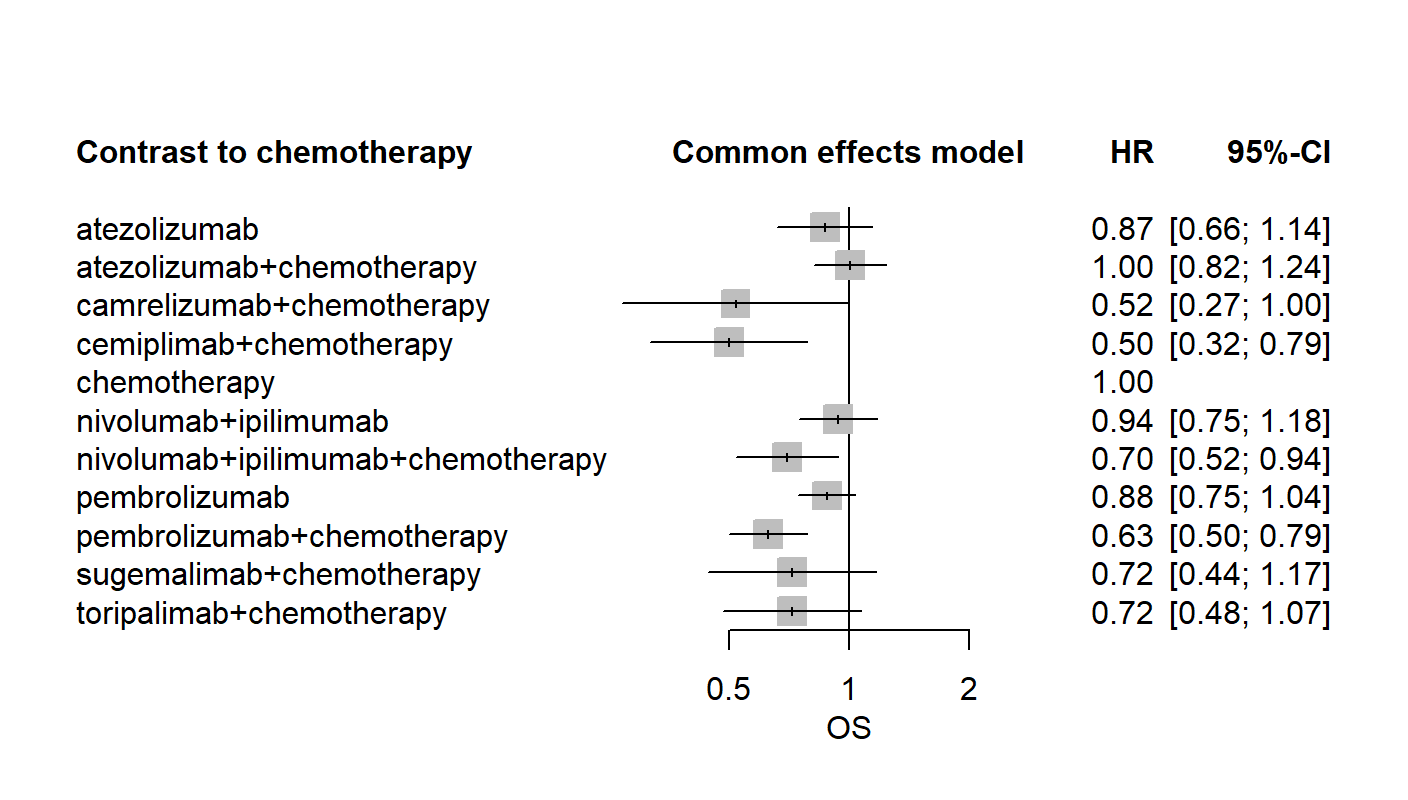
**

(B)

**Supplementary Figure 10.** Network diagram(A) and Forest plots(B) for the HR of overall survival for the PD-L1 1-49%. Separate model, Whole network level (I^2^= 15.9%, Total; P= 0.3119, Within designs; P = 0.3119, Between designs; P=0)

**
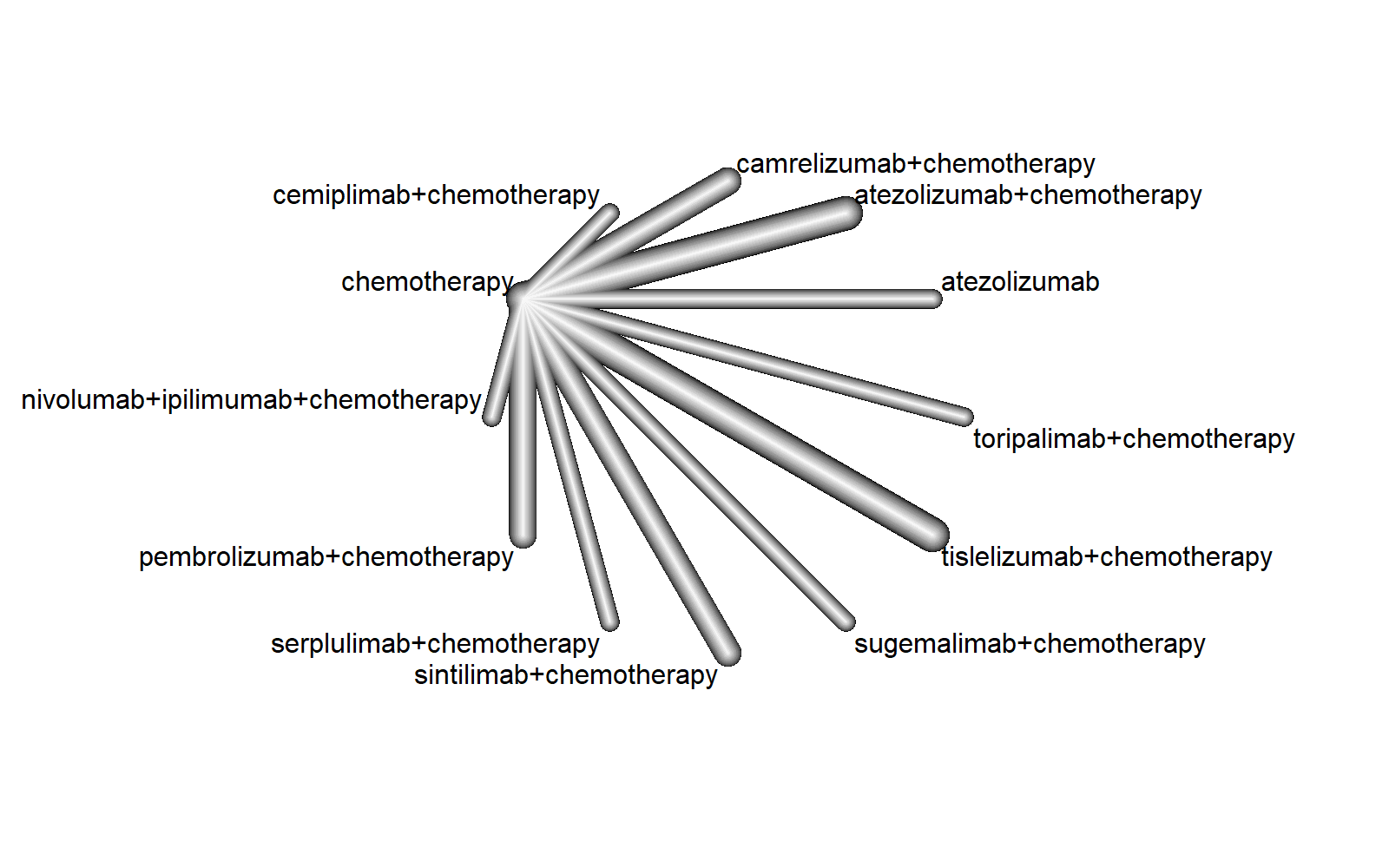
**

(A)

**
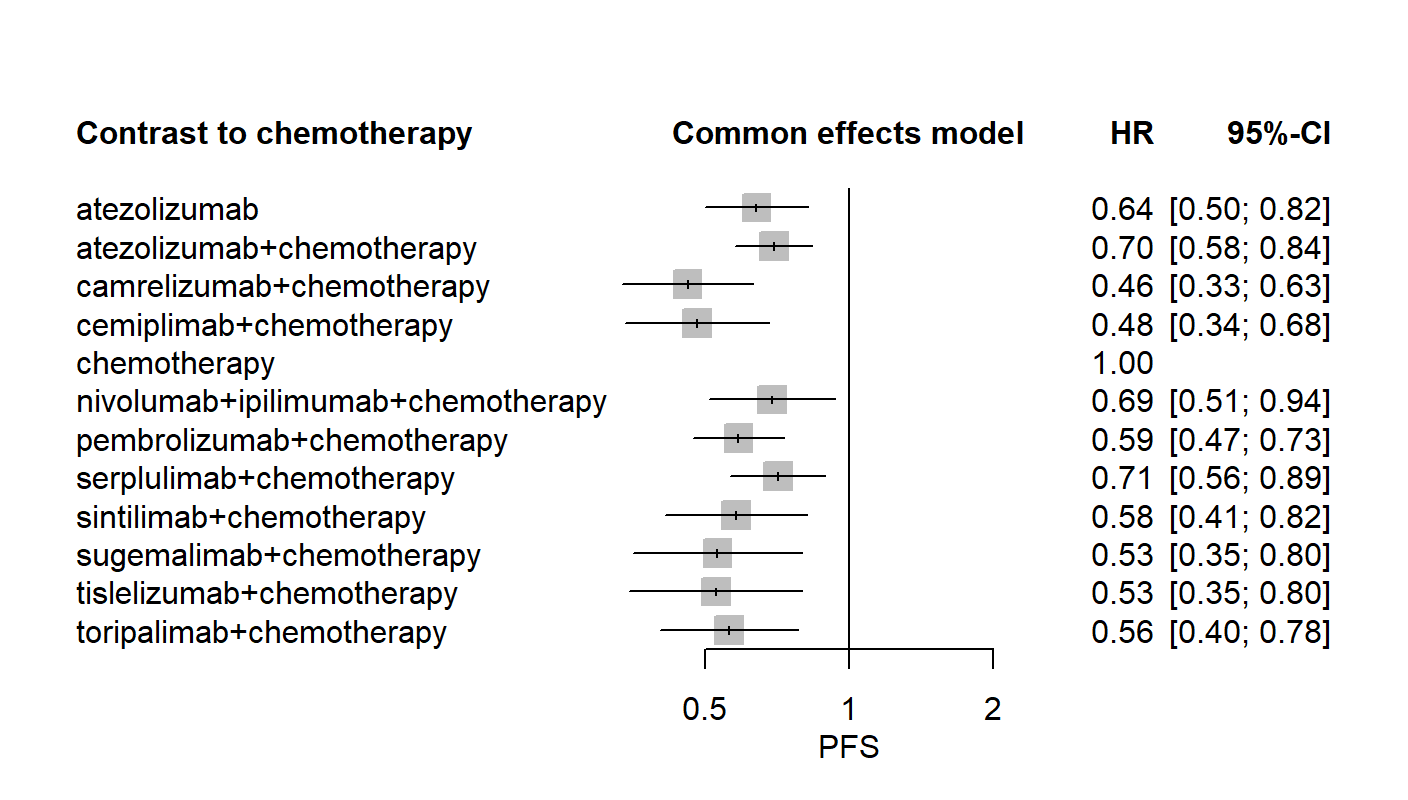
**

(B)

**Supplementary Figure 11.** Network diagram(A) and Forest plots(B) for the HR of progression-free survival for the PD-L1 1-49%. Separate model, Whole network level (I2= 37.8%, Total; P= 0.1279, Within designs; P = 0.1279, Between designs; P=0)

**
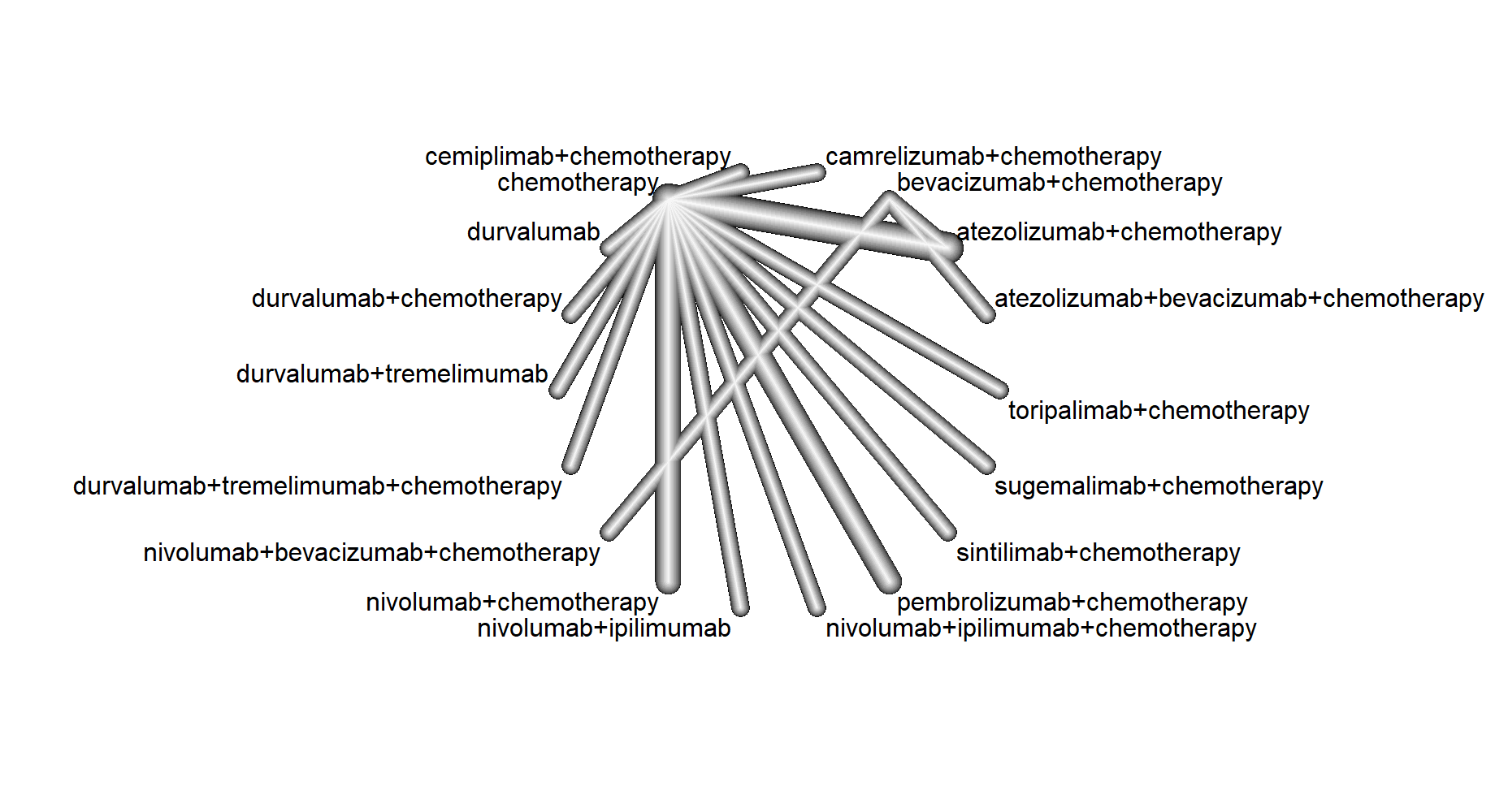
**

(A)

**
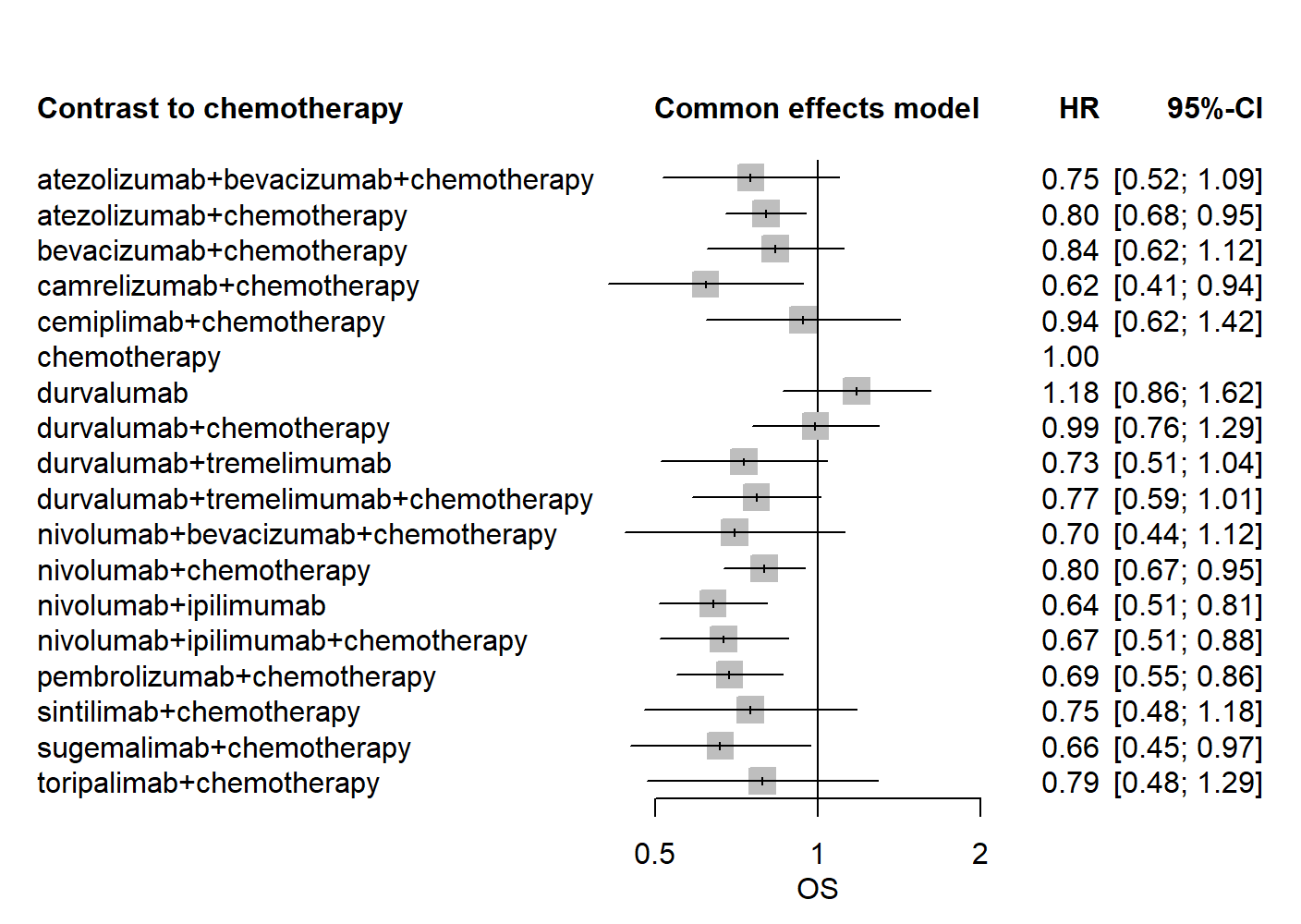
**

(B)

**Supplementary Figure 12.** Network diagram(A) and Forest plots(B) for the HR of overall survival for the PD-L1＜1%. Separate model, Whole network level (I2= 13.5%, Total; P= 0.3279, Within designs; P= 0.3279, Between designs; P=0)

**
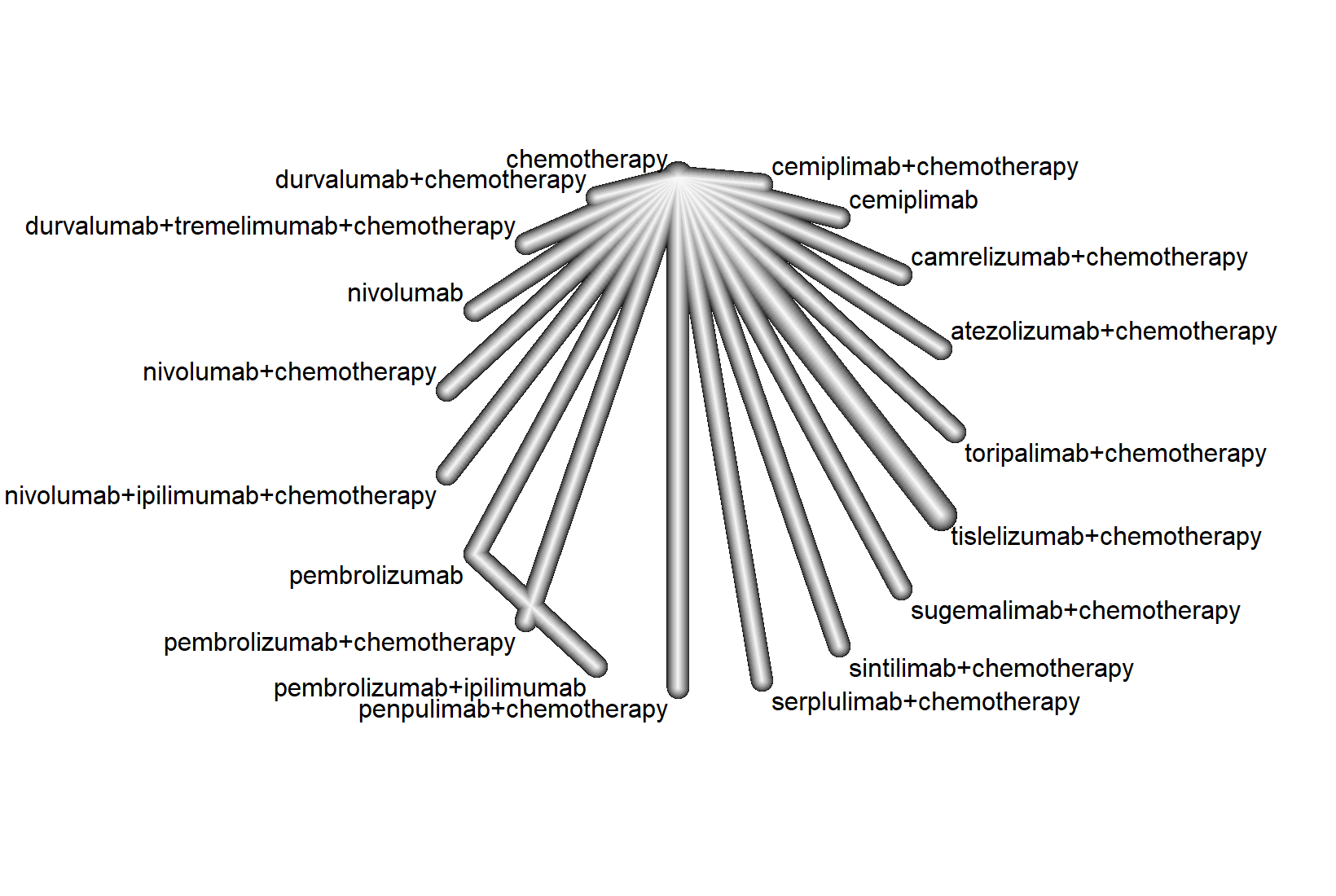
**

(A)

**
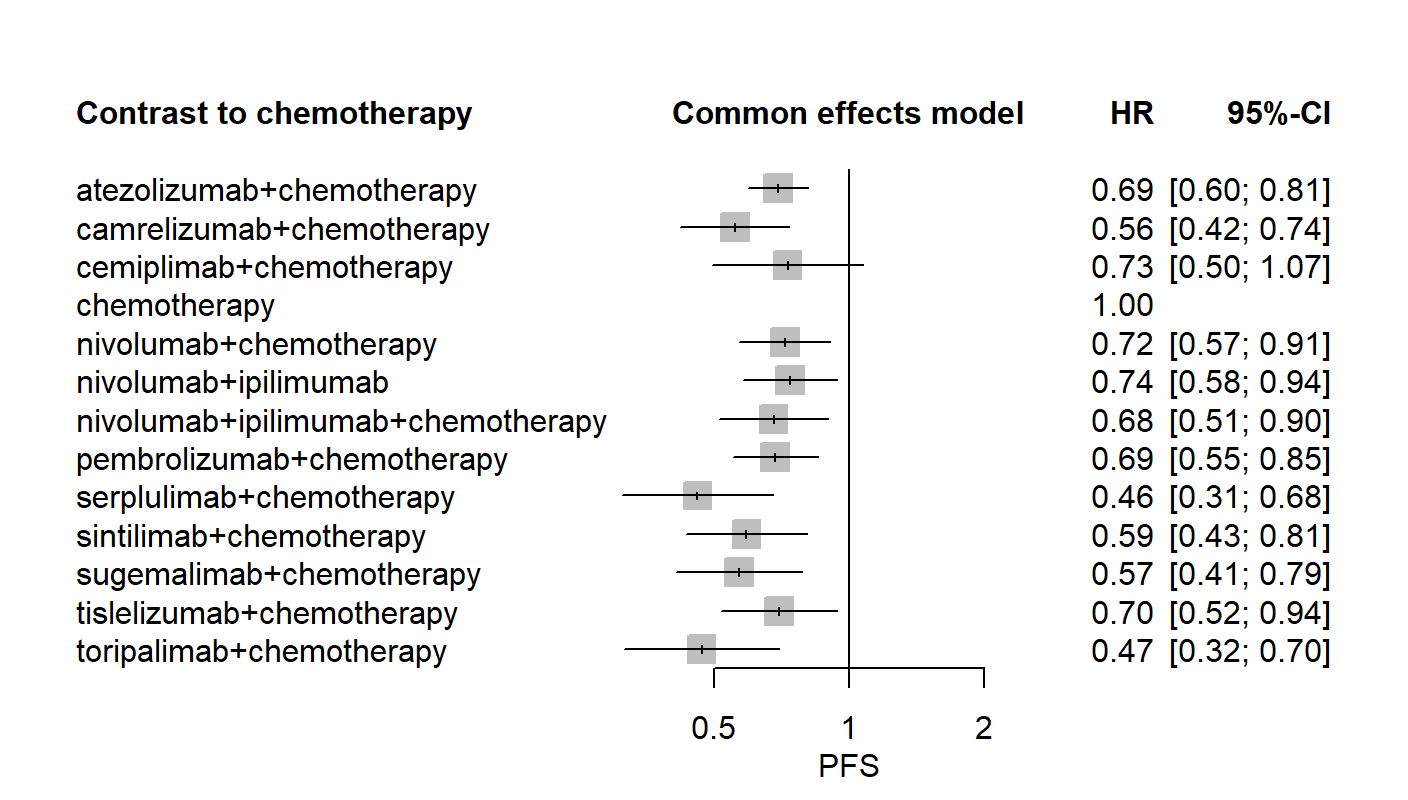
**

(B)

**Supplementary Figure 13.** Network diagram(A) and Forest plots(B) for the HR of progression-free survival for the PD-L1＜1%. Separate model, Whole network level (I2= 22.8%, Total; P= 0.2549, Within designs; P = 0.2549, Between designs; P=0)

## Supplementary Tables

**Supplementary Table 1.** Search strategy

| **Search strategy for PubMed, EMBASE, and Cochrane Library databases** | | |
| --- | --- | --- |
| Patient | #1 | (((("lung"[Title/Abstract]) AND ((("Non Small Cell"[Title/Abstract]) OR "Non-Small-Cell"[Title/Abstract]) OR "Non-Small Cell"[Title/Abstract]))) OR Carcinoma, Non-Small-Cell Lung[MeSH Terms]) OR "NSCLC"[Title/Abstract] |
| Intervention | #2 | ((((((((("Ipilimumab"[MeSH Terms]) OR "Yervoy"[Title/Abstract]) OR "MDX010"[Title/Abstract]) OR "MDX-010"[Title/Abstract])) OR (((("durvalumab" [Supplementary Concept]) OR "Imfinzi"[Title/Abstract]) OR "MEDI4736"[Title/Abstract]) OR "MEDI-4736"[Title/Abstract])) OR ((((("atezolizumab"[Supplementary Concept]) OR "Tecentriq"[Title/Abstract]) OR "MPDL3280A"[Title/Abstract]) OR "RG7446"[Title/Abstract]) OR "RG-7446"[Title/Abstract])) OR ((((("MDX-1106"[Title/Abstract]) OR "ONO-4538"[Title/Abstract]) OR "BMS-936558"[Title/Abstract]) OR "nivolumab"[MeSH Terms]) OR "Opdivo"[Title/Abstract])) OR (((("Keytruda"[Title/Abstract]) OR "lambrolizumab"[Title/Abstract]) OR "MK-3475"[Title/Abstract]) OR "pembrolizumab" [Supplementary Concept])) OR (((((((((((("anti-PDL1"[Title/Abstract]) OR "anti-PD1"[Title/Abstract]) OR "PD-1"[Title/Abstract]) OR "PD1"[Title/Abstract]) OR "PD 1"[Title/Abstract]) OR "PD-L1"[Title/Abstract]) OR "PD L1"[Title/Abstract]) OR "PDL1"[Title/Abstract]) OR "Programmed Death 1"[Title/Abstract]) OR "Programmed Cell Death 1 Receptor"[Title/Abstract]) OR "Programmed Death-Ligand 1"[Title/Abstract]) OR "programmed cell death 1 ligand 1 protein"[Title/Abstract]) |
| Study type | #3 | ((clinical trials as topic[MeSH Terms]) OR "trial"[Title/Abstract]) OR "study"[Title/Abstract] |
| Language | #4 | English [language] |
| Search strategy | #1 AND #2 AND #3 AND #4 | |

**Supplementary Table 2.** Quality assessment of RCTs

| Study | Random sequence generation | Allocation concealment | Blinding of participants and personnel | Blinding of outcome assessment | Incomplete outcome data | Selective reporting |
| --- | --- | --- | --- | --- | --- | --- |
| AK105-302 | Low | Low | Low | Low | Low | Low |
| ASTRUM-004 | Low | Low | Low | Low | Low | Low |
| CameL | Low | Low | High | Low | Low | Low |
| CameL-sq | Low | Low | Low | Low | Low | Low |
| CheckMate 026 | Unclear | Unclear | High | Low | Low | Low |
| CheckMate 227-part 1 | Unclear | Unclear | High | Low | Low | Low |
| CheckMate 227-part 2 | Unclear | Unclear | High | Low | Low | Low |
| CheckMate 9LA | Low | Low | High | Low | Low | Low |
| CHOICE-01 | Low | Low | Low | Low | Low | Low |
| EMPOWER-Lung 1 | Low | Low | High | Low | Low | Low |
| EMPOWER-Lung 3 | Low | Low | Low | Low | Low | Low |
| GEMSTONE-302 | Low | Low | Low | Low | Low | Low |
| IMpower110 | Unclear | Unclear | High | Low | Low | Low |
| IMpower130 | Low | Low | High | Low | Low | Low |
| IMpower131 | Unclear | Unclear | High | Low | Low | Low |
| IMpower132 | Unclear | Unclear | High | Low | Low | Low |
| IMpower150 | Unclear | Unclear | High | Low | Low | Low |
| KEYNOTE-024 | Unclear | Unclear | High | Low | Low | Low |
| KEYNOTE-042 | Low | Unclear | High | Low | Low | Low |
| KEYNOTE-189 | Low | Low | Low | Low | Low | Low |
| KEYNOTE-407 | Low | Low | Low | Low | Low | Low |
| KEYNOTE-598 | Unclear | Unclear | Low | Low | Low | Low |
| MYSTIC | Low | Low | High | Low | Low | Low |
| ONO-4538-52/TASUKI-52 | Unclear | Unclear | Low | Low | Low | Low |
| ORIENT-11 | Low | Low | Low | Low | Low | Low |
| ORIENT-12 | Low | Low | Low | Low | Low | Low |
| POSEIDON | Unclear | Unclear | Low | Low | Low | Low |
| RATIONALE 304 | Unclear | Unclear | High | Low | Low | Low |
| RATIONALE 307 | Low | Low | High | Low | Low | Low |

**Supplementary Table 3.** Pairwise comparisons from the Network Meta-analysis on OS. Each cell of the efficacy contains the pooled HR and 95% confidence intervals for OS; significant results are in bold.

| ate | . | . | . | . | . | . |  | . | . | . | . | . | . | . | . | . | . | . | . | . | . | . | . | . | . |
| --- | --- | --- | --- | --- | --- | --- | --- | --- | --- | --- | --- | --- | --- | --- | --- | --- | --- | --- | --- | --- | --- | --- | --- | --- | --- |
| 1.04 (0.74; 1.46) | ate+bev+che | . |  | . | . | . |  | . | . | . | . | . | . | . | . | . | . | . | . | . | . | . | . | . | . |
| 0.99 (0.78; 1.25) | 0.95 (0.75; 1.22) | ate+che |  | . | . | . |  | . | . | . | . | . | . | . | . | . | . | . | . | . | . | . | . | . | . |
| 0.83 (0.62; 1.11) | **0.80 (0.67; 0.95)** | **0.84 (0.71; 1.00)** | bev+che | . | . | . |  | . | . | . | . | . |  | . | . | . | . | . | . | . | . | . | . | . | . |
| 1.32 (0.98; 1.77) | 1.27 (0.90; 1.79) | **1.33 (1.05; 1.69)** | **1.59 (1.18; 2.13)** | cam+che | . | . |  | . | . | . | . | . | . | . | . | . | . | . | . | . | . | . | . | . | . |
| **1.49 (1.11; 2.01)** | **1.44 (1.01; 2.03)** | **1.51 (1.18; 1.93)** | **1.79 (1.33; 2.42)** | 1.13 (0.84; 1.53) | cem | . |  | . | . | . | . | . | . | . | . | . | . | . | . | . | . | . | . | . | . |
| 1.31 (0.96; 1.79) | 1.26 (0.88; 1.80) | **1.32 (1.01; 1.72)** | **1.57 (1.15; 2.16)** | 0.99 (0.72; 1.36) | 0.88 (0.64; 1.21) | cem+che |  | . | . | . | . | . | . | . | . | . | . | . | . | . | . | . | . | . | . |
| 0.85 (0.69; 1.04) | 0.82 (0.62; 1.07) | **0.86 (0.76; 0.97)** | 1.02 (0.83; 1.26) | **0.64 (0.52; 0.79)** | **0.57 (0.46; 0.71)** | **0.65 (0.51; 0.82)** | che |  |  |  |  |  |  |  |  |  |  |  |  |  |  |  |  |  |  |
| 0.89 (0.68; 1.15) | 0.85 (0.62; 1.17) | 0.89 (0.73; 1.10) | 1.07 (0.82; 1.39) | **0.67 (0.51; 0.88)** | **0.59 (0.45; 0.78)** | **0.68 (0.51; 0.90)** | 1.04 (0.88; 1.23) | dur | . | . | . | . | . | . | . | . | . | . | . | . | . | . | . | . | . |
| 0.99 (0.76; 1.29) | 0.95 (0.69; 1.31) | 1.00 (0.81; 1.23) | 1.19 (0.91; 1.56) | **0.75 (0.57; 0.98)** | **0.66 (0.50; 0.88)** | 0.76 (0.56; 1.01) | 1.16 (0.98; 1.38) | 1.12 (0.88; 1.42) | dur+che | . | . | . | . | . | . | . | . | . | . | . | . | . | . | . | . |
| 0.90 (0.69; 1.18) | 0.87 (0.63; 1.20) | 0.91 (0.75; 1.12) | 1.09 (0.83; 1.42) | **0.69 (0.53; 0.89)** | **0.61 (0.46; 0.80)** | **0.69 (0.52; 0.92)** | 1.06 (0.90; 1.26) | 1.02 (0.81; 1.29) | 0.91 (0.72; 1.16) | dur+tre | . | . | . | . | . | . | . | . | . | . | . | . | . | . | . |
| 1.10 (0.84; 1.44) | 1.06 (0.77; 1.47) | 1.12 (0.91; 1.38) | **1.33 (1.01; 1.74)** | 0.84 (0.64; 1.10) | **0.74 (0.56; 0.98)** | 0.84 (0.63; 1.13) | **1.30 (1.09; 1.55)** | 1.25 (0.98; 1.59) | 1.12 (0.87; 1.43) | 1.22 (0.96; 1.55) | dur+tre+che | . | . | . | . | . | . | . | . | . | . | . | . | . | . |
| 0.88 (0.69; 1.12) | 0.85 (0.63; 1.14) | 0.89 (0.75; 1.06) | 1.06 (0.83; 1.35) | **0.67 (0.52; 0.85)** | **0.59 (0.46; 0.76)** | **0.67 (0.52; 0.88)** | 1.04 (0.92; 1.18) | 1.00 (0.81; 1.23) | 0.89 (0.72; 1.11) | 0.98 (0.79; 1.20) | **0.80 (0.65; 0.99)** | niv | . | . | . | . | . | . | . | . | . | . | . | . | . |
| 1.12 (0.74; 1.70) | 1.08 (0.77; 1.53) | 1.14 (0.81; 1.60) | **1.35 (1.00; 1.82)** | 0.85 (0.56; 1.29) | 0.75 (0.49; 1.15) | 0.86 (0.56; 1.32) | 1.32 (0.92; 1.90) | 1.27 (0.85; 1.89) | 1.14 (0.76; 1.70) | 1.24 (0.83; 1.85) | 1.02 (0.68; 1.52) | 1.27 (0.87; 1.87) | niv+bev+che | . | . | . | . | . | . | . | . | . | . | . | . |
| 1.04 (0.81; 1.34) | 1.01 (0.74; 1.37) | 1.06 (0.88; 1.27) | 1.26 (0.98; 1.62) | 0.79 (0.61; 1.02) | **0.70 (0.54; 0.91)** | 0.80 (0.61; 1.05) | **1.23 (1.06; 1.42)** | 1.18 (0.95; 1.47) | 1.06 (0.84; 1.32) | 1.15 (0.93; 1.44) | 0.95 (0.76; 1.18) | 1.18 (0.98; 1.43) | 0.93 (0.63; 1.37) | niv+che | . | . | . | . | . | . | . | . | . | . | . |
| 1.18 (0.93; 1.51) | 1.14 (0.84; 1.54) | **1.19 (1.00; 1.42)** | **1.42 (1.11; 1.82)** | 0.90 (0.70; 1.15) | 0.79 (0.61; 1.02) | 0.90 (0.69; 1.18) | **1.39 (1.22; 1.58)** | **1.33 (1.08; 1.65)** | 1.19 (0.96; 1.49) | **1.31 (1.06; 1.61)** | 1.07 (0.86; 1.33) | **1.34 (1.12; 1.61)** | 1.05 (0.72; 1.54) | 1.13 (0.93; 1.37) | niv+ipi | . | . | . | . | . | . | . | . | . | . |
| 1.15 (0.88; 1.49) | 1.11 (0.81; 1.52) | 1.16 (0.95; 1.42) | **1.38 (1.06; 1.80)** | 0.87 (0.67; 1.13) | 0.77 (0.59; 1.01) | 0.88 (0.66; 1.17) | **1.35 (1.15; 1.59)** | **1.30 (1.03; 1.64)** | 1.16 (0.92; 1.47) | **1.27 (1.01; 1.60)** | 1.04 (0.82; 1.32) | **1.30 (1.06; 1.60)** | 1.02 (0.69; 1.52) | 1.10 (0.89; 1.36) | 0.97 (0.79; 1.20) | niv+ipi+che | . | . | . | . | . | . | . | . | . |
| 1.12 (0.89; 1.42) | 1.08 (0.81; 1.45) | 1.13 (0.97; 1.33) | **1.35 (1.07; 1.71)** | 0.85 (0.67; 1.08) | **0.75 (0.59; 0.96)** | 0.86 (0.66; 1.11) | **1.32 (1.18; 1.47)** | **1.27 (1.04; 1.55)** | 1.14 (0.92; 1.39) | **1.24 (1.02; 1.51)** | 1.02 (0.83; 1.25) | **1.27 (1.08; 1.50)** | 1.00 (0.68; 1.46) | 1.07 (0.90; 1.29) | 0.95 (0.80; 1.13) | 0.98 (0.80; 1.19) | pem | . |  | . | . | . | . | . | . |
| **1.30 (1.02; 1.66)** | 1.25 (0.93; 1.69) | **1.32 (1.11; 1.57)** | **1.57 (1.23; 2.00)** | 0.99 (0.77; 1.26) | 0.87 (0.68; 1.12) | 1.00 (0.76; 1.31) | **1.53 (1.35; 1.74)** | **1.47 (1.19; 1.82)** | **1.32 (1.06; 1.64)** | **1.44 (1.17; 1.78)** | 1.18 (0.95; 1.46) | **1.48 (1.23; 1.77)** | 1.16 (0.79; 1.70) | **1.25 (1.03; 1.51)** | 1.10 (0.92; 1.33) | 1.13 (0.92; 1.39) | 1.16 (0.98; 1.37) | pem+che | . | . | . | . | . | . | . |
| 1.04 (0.74; 1.45) | 1.00 (0.69; 1.46) | 1.05 (0.79; 1.40) | 1.25 (0.89; 1.75) | 0.79 (0.56; 1.10) | **0.70 (0.50; 0.98)** | 0.79 (0.56; 1.13) | 1.22 (0.94; 1.59) | 1.17 (0.86; 1.60) | 1.05 (0.77; 1.44) | 1.15 (0.84; 1.57) | 0.94 (0.69; 1.29) | 1.18 (0.88; 1.58) | 0.93 (0.59; 1.45) | 1.00 (0.74; 1.34) | 0.88 (0.66; 1.18) | 0.90 (0.66; 1.23) | 0.93 (0.73; 1.18) | 0.80 (0.60; 1.07) | pem+ipi | . | . | . | . | . | . |
| **1.55 (1.06; 2.25)** | 1.49 (0.98; 2.25) | **1.56 (1.12; 2.18)** | **1.86 (1.28; 2.71)** | 1.17 (0.80; 1.71) | 1.04 (0.71; 1.52) | 1.18 (0.80; 1.75) | **1.82 (1.33; 2.49)** | **1.75 (1.22; 2.49)** | **1.56 (1.09; 2.24)** | **1.71 (1.20; 2.44)** | 1.40 (0.98; 2.00) | **1.75 (1.25; 2.46)** | 1.38 (0.85; 2.22) | **1.48 (1.05; 2.09)** | 1.31 (0.93; 1.84) | 1.35 (0.94; 1.92) | 1.38 (0.99; 1.92) | 1.19 (0.84; 1.67) | 1.49 (0.99; 2.24) | pen+che | . | . | . | . | . |
| 1.16 (0.85; 1.59) | 1.12 (0.78; 1.61) | 1.18 (0.90; 1.53) | **1.40 (1.02; 1.92)** | 0.88 (0.64; 1.21) | 0.78 (0.57; 1.08) | 0.89 (0.64; 1.24) | **1.37 (1.08; 1.73)** | 1.32 (0.99; 1.76) | 1.18 (0.88; 1.58) | 1.29 (0.97; 1.72) | 1.05 (0.79; 1.41) | **1.32 (1.01; 1.72)** | 1.04 (0.67; 1.60) | 1.12 (0.85; 1.47) | 0.99 (0.75; 1.29) | 1.01 (0.76; 1.35) | 1.04 (0.80; 1.35) | 0.89 (0.68; 1.17) | 1.12 (0.79; 1.59) | 0.75 (0.51; 1.12) | ser+che | . | . | . | . |
| 1.35 (0.99; 1.84) | 1.30 (0.91; 1.86) | **1.37 (1.05; 1.77)** | **1.63 (1.19; 2.22)** | 1.02 (0.75; 1.40) | 0.91 (0.66; 1.24) | 1.03 (0.74; 1.44) | **1.59 (1.26; 2.00)** | **1.53 (1.15; 2.03)** | **1.37 (1.02; 1.83)** | **1.49 (1.12; 1.99)** | 1.22 (0.92; 1.63) | **1.53 (1.18; 1.99)** | 1.20 (0.78; 1.85) | 1.29 (0.99; 1.70) | 1.14 (0.88; 1.49) | 1.18 (0.89; 1.56) | 1.20 (0.93; 1.55) | 1.04 (0.80; 1.35) | 1.30 (0.92; 1.84) | 0.87 (0.59; 1.29) | 1.16 (0.83; 1.61) | sin+che | . | . | . |
| 1.31 (0.94; 1.82) | 1.26 (0.87; 1.83) | 1.32 (0.99; 1.76) | **1.57 (1.13; 2.19)** | 0.99 (0.71; 1.38) | 0.88 (0.63; 1.23) | 1.00 (0.70; 1.42) | **1.54 (1.19; 1.99)** | **1.48 (1.09; 2.01)** | 1.32 (0.97; 1.81) | **1.45 (1.06; 1.97)** | 1.18 (0.87; 1.62) | **1.48 (1.11; 1.98)** | 1.16 (0.75; 1.82) | 1.25 (0.93; 1.68) | 1.11 (0.83; 1.48) | 1.14 (0.84; 1.55) | 1.17 (0.88; 1.54) | 1.00 (0.75; 1.34) | 1.26 (0.87; 1.82) | 0.85 (0.56; 1.27) | 1.12 (0.79; 1.59) | 0.97 (0.68; 1.37) | sug+che | . | . |
| 1.34 (0.99; 1.82) | 1.29 (0.91; 1.84) | **1.36 (1.05; 1.75)** | **1.62 (1.19; 2.19)** | 1.02 (0.75; 1.38) | 0.90 (0.66; 1.23) | 1.03 (0.74; 1.42) | **1.58 (1.26; 1.98)** | **1.52 (1.15; 2.01)** | **1.36 (1.02; 1.81)** | **1.48 (1.12; 1.96)** | 1.22 (0.91; 1.62) | **1.52 (1.18; 1.97)** | 1.20 (0.78; 1.83) | 1.29 (0.98; 1.68) | 1.14 (0.88; 1.48) | 1.17 (0.89; 1.54) | 1.20 (0.93; 1.54) | 1.03 (0.79; 1.34) | 1.29 (0.91; 1.83) | 0.87 (0.59; 1.28) | 1.15 (0.83; 1.60) | 0.99 (0.72; 1.37) | 1.03 (0.73; 1.45) | tis+che | . |
| 1.23 (0.87; 1.75) | 1.19 (0.80; 1.76) | 1.25 (0.91; 1.69) | **1.48 (1.04; 2.11)** | 0.93 (0.66; 1.33) | 0.83 (0.58; 1.18) | 0.94 (0.65; 1.37) | **1.45 (1.09; 1.93)** | **1.39 (1.00; 1.94)** | 1.25 (0.89; 1.74) | 1.36 (0.98; 1.89) | 1.12 (0.80; 1.56) | **1.40 (1.02; 1.91)** | 1.10 (0.69; 1.74) | 1.18 (0.86; 1.62) | 1.04 (0.76; 1.43) | 1.07 (0.77; 1.49) | 1.10 (0.81; 1.49) | 0.95 (0.69; 1.29) | 1.19 (0.80; 1.75) | 0.80 (0.52; 1.22) | 1.06 (0.73; 1.53) | 0.91 (0.63; 1.32) | 0.94 (0.64; 1.39) | 0.92 (0.64; 1.32) | tor+che |

**Supplementary Table 4.** Pairwise comparisons from the Network Meta-analysis on PFS. Each cell of the efficacy contains the pooled HR and 95% confidence intervals for PFS; significant results are in bold.

| ate | . | . | . | . |  | . | . | . | . | . | . | . | . | . | . | . | . | . | . | . | . | . |
| --- | --- | --- | --- | --- | --- | --- | --- | --- | --- | --- | --- | --- | --- | --- | --- | --- | --- | --- | --- | --- | --- | --- |
| 1.12 (0.70; 1.79) | ate+che | . | . | . |  | . | . | . | . | . | . | . | . | . | . | . | . | . | . | . | . | . |
| 1.55 (0.92; 2.59) | 1.38 (0.93; 2.05) | cam+che | . | . |  | . | . | . | . | . | . | . | . | . | . | . | . | . | . | . | . | . |
| 1.41 (0.79; 2.52) | 1.26 (0.78; 2.03) | 0.91 (0.54; 1.54) | cem | . |  | . | . | . | . | . | . | . | . | . | . | . | . | . | . | . | . | . |
| 1.31 (0.73; 2.36) | 1.17 (0.72; 1.90) | 0.85 (0.50; 1.44) | 0.93 (0.51; 1.68) | cem+che |  | . | . | . | . | . | . | . | . | . | . | . | . | . | . | . | . | . |
| 0.72 (0.48; 1.08) | **0.64 (0.51; 0.81)** | **0.47 (0.34; 0.64)** | **0.51 (0.34; 0.77)** | **0.55 (0.36; 0.84)** | che |  |  |  |  |  |  |  |  |  |  |  |  |  |  |  |  |  |
| 0.83 (0.45; 1.52) | 0.74 (0.44; 1.23) | **0.53 (0.31; 0.93)** | 0.59 (0.32; 1.08) | 0.63 (0.34; 1.18) | 1.15 (0.73; 1.81) | dur | . | . | . | . | . | . | . | . | . | . | . | . | . | . | . | . |
| 0.97 (0.55; 1.73) | 0.87 (0.54; 1.39) | 0.63 (0.38; 1.05) | 0.69 (0.39; 1.23) | 0.74 (0.41; 1.34) | 1.35 (0.90; 2.03) | 1.18 (0.64; 2.17) | dur+che | . | . | . | . | . | . | . | . | . | . | . | . | . | . | . |
| 0.58 (0.32; 1.02) | **0.51 (0.32; 0.82)** | **0.37 (0.22; 0.62)** | **0.41 (0.23; 0.73)** | **0.44 (0.24; 0.79)** | 0.80 (0.53; 1.20) | 0.70 (0.38; 1.28) | 0.59 (0.33; 1.05) | dur+tre | . | . | . | . | . | . | . | . | . | . | . | . | . | . |
| 1.00 (0.56; 1.78) | 0.89 (0.56; 1.43) | 0.65 (0.39; 1.08) | 0.71 (0.40; 1.27) | 0.76 (0.42; 1.38) | 1.39 (0.92; 2.09) | 1.21 (0.66; 2.23) | 1.03 (0.58; 1.83) | 1.74 (0.98; 3.08) | dur+tre+che | . | . | . | . | . | . | . | . | . | . | . | . | . |
| 0.68 (0.42; 1.10) | **0.61 (0.43; 0.86)** | **0.44 (0.29; 0.66)** | **0.48 (0.30; 0.79)** | **0.52 (0.32; 0.86)** | 0.95 (0.73; 1.22) | 0.82 (0.49; 1.39) | 0.70 (0.43; 1.13) | 1.18 (0.73; 1.91) | 0.68 (0.42; 1.10) | niv | . |  | . | . | . | . | . | . | . | . | . | . |
| 1.08 (0.66; 1.79) | 0.97 (0.66; 1.41) | 0.70 (0.45; 1.08) | 0.77 (0.46; 1.28) | 0.83 (0.49; 1.39) | **1.51 (1.12; 2.02)** | 1.31 (0.76; 2.25) | 1.11 (0.67; 1.84) | **1.88 (1.14; 3.10)** | 1.08 (0.66; 1.79) | **1.59 (1.08; 2.35)** | niv+che | . | . | . | . | . | . | . | . | . | . | . |
| 0.88 (0.53; 1.47) | 0.79 (0.54; 1.16) | **0.57 (0.37; 0.88)** | 0.62 (0.37; 1.04) | 0.67 (0.40; 1.14) | 1.23 (0.90; 1.66) | 1.07 (0.62; 1.84) | 0.91 (0.55; 1.51) | 1.53 (0.92; 2.54) | 0.88 (0.53; 1.47) | 1.29 (0.95; 1.76) | 0.81 (0.53; 1.24) | niv+ipi | . | . | . | . | . | . | . | . | . | . |
| 1.07 (0.61; 1.91) | 0.96 (0.60; 1.53) | 0.69 (0.42; 1.16) | 0.76 (0.43; 1.36) | 0.82 (0.46; 1.47) | **1.49 (1.00; 2.23)** | 1.30 (0.71; 2.39) | 1.10 (0.62; 1.96) | **1.87 (1.05; 3.30)** | 1.07 (0.61; 1.91) | 1.58 (0.98; 2.54) | 0.99 (0.60; 1.63) | 1.22 (0.73; 2.02) | niv+ipi+che | . | . | . | . | . | . | . | . | . |
| 0.95 (0.58; 1.57) | 0.85 (0.59; 1.23) | **0.62 (0.40; 0.95)** | 0.67 (0.41; 1.12) | 0.73 (0.43; 1.22) | 1.32 (0.99; 1.77) | 1.15 (0.67; 1.97) | 0.98 (0.59; 1.61) | **1.65 (1.00; 2.72)** | 0.95 (0.58; 1.57) | 1.40 (0.95; 2.06) | 0.88 (0.58; 1.33) | 1.08 (0.71; 1.64) | 0.89 (0.54; 1.46) | pem | . |  | . | . | . | . | . | . |
| 1.29 (0.79; 2.13) | 1.15 (0.80; 1.67) | 0.84 (0.54; 1.28) | 0.92 (0.55; 1.51) | 0.99 (0.59; 1.65) | **1.80 (1.35; 2.39)** | 1.56 (0.91; 2.68) | 1.33 (0.81; 2.19) | **2.24 (1.37; 3.69)** | 1.29 (0.79; 2.13) | **1.90 (1.29; 2.79)** | 1.19 (0.79; 1.80) | 1.47 (0.96; 2.23) | 1.20 (0.73; 1.97) | 1.36 (0.90; 2.04) | pem+che | . | . | . | . | . | . | . |
| 0.90 (0.47; 1.72) | 0.80 (0.46; 1.41) | 0.58 (0.32; 1.06) | 0.64 (0.33; 1.23) | 0.69 (0.35; 1.33) | 1.25 (0.75; 2.08) | 1.08 (0.55; 2.15) | 0.92 (0.48; 1.77) | 1.56 (0.81; 2.99) | 0.90 (0.47; 1.72) | 1.32 (0.74; 2.33) | 0.83 (0.46; 1.49) | 1.02 (0.56; 1.84) | 0.84 (0.44; 1.60) | 0.94 (0.62; 1.43) | 0.69 (0.39; 1.25) | pem+ipi | . | . | . | . | . | . |
| 1.67 (0.91; 3.07) | 1.50 (0.90; 2.48) | 1.08 (0.62; 1.88) | 1.19 (0.64; 2.19) | 1.28 (0.69; 2.38) | **2.33 (1.48; 3.65)** | **2.02 (1.07; 3.84)** | 1.72 (0.94; 3.16) | **2.91 (1.59; 5.33)** | 1.67 (0.91; 3.07) | **2.46 (1.46; 4.12)** | 1.55 (0.90; 2.65) | **1.90 (1.10; 3.27)** | 1.56 (0.85; 2.85) | **1.76 (1.03; 3.01)** | 1.30 (0.76; 2.21) | 1.86 (0.94; 3.68) | pen+che | . | . | . | . | . |
| 1.36 (0.75; 2.46) | 1.21 (0.74; 1.98) | 0.88 (0.51; 1.50) | 0.96 (0.53; 1.75) | 1.04 (0.57; 1.90) | **1.89 (1.22; 2.91)** | 1.64 (0.88; 3.08) | 1.40 (0.77; 2.53) | **2.36 (1.30; 4.27)** | 1.36 (0.75; 2.46) | **1.99 (1.20; 3.30)** | 1.25 (0.74; 2.12) | 1.54 (0.91; 2.62) | 1.26 (0.70; 2.28) | 1.43 (0.85; 2.41) | 1.05 (0.62; 1.77) | 1.51 (0.77; 2.96) | 0.81 (0.43; 1.52) | ser+che | . | . | . | . |
| 1.41 (0.84; 2.37) | 1.26 (0.85; 1.87) | 0.91 (0.58; 1.43) | 1.00 (0.59; 1.69) | 1.08 (0.63; 1.83) | **1.96 (1.43; 2.70)** | 1.71 (0.98; 2.97) | 1.45 (0.87; 2.43) | **2.45 (1.47; 4.10)** | 1.41 (0.84; 2.37) | **2.07 (1.38; 3.11)** | 1.30 (0.84; 2.01) | **1.60 (1.03; 2.49)** | 1.31 (0.79; 2.20) | 1.48 (0.96; 2.28) | 1.09 (0.71; 1.68) | 1.57 (0.86; 2.87) | 0.84 (0.49; 1.46) | 1.04 (0.61; 1.78) | sin+che | . | . | . |
| 1.50 (0.83; 2.70) | 1.34 (0.83; 2.17) | 0.97 (0.57; 1.65) | 1.06 (0.59; 1.92) | 1.15 (0.63; 2.09) | **2.08 (1.36; 3.18)** | 1.81 (0.97; 3.38) | 1.54 (0.86; 2.77) | **2.60 (1.45; 4.68)** | 1.50 (0.83; 2.70) | **2.20 (1.34; 3.61)** | 1.38 (0.83; 2.32) | **1.70 (1.01; 2.87)** | 1.40 (0.78; 2.51) | 1.58 (0.94; 2.64) | 1.16 (0.70; 1.94) | 1.67 (0.86; 3.24) | 0.90 (0.48; 1.66) | 1.10 (0.60; 2.02) | 1.06 (0.63; 1.80) | sug+che | . | . |
| 1.45 (0.88; 2.37) | 1.29 (0.90; 1.86) | 0.93 (0.61; 1.42) | 1.02 (0.62; 1.69) | 1.10 (0.66; 1.83) | **2.01 (1.52; 2.65)** | **1.75 (1.02; 2.98)** | 1.49 (0.91; 2.43) | **2.51 (1.54; 4.10)** | 1.45 (0.88; 2.37) | **2.12 (1.45; 3.09)** | 1.33 (0.89; 2.00) | **1.64 (1.08; 2.48)** | 1.34 (0.82; 2.20) | **1.52 (1.01; 2.27)** | 1.12 (0.75; 1.67) | 1.61 (0.90; 2.88) | 0.86 (0.51; 1.47) | 1.06 (0.64; 1.78) | 1.02 (0.67; 1.56) | 0.96 (0.58; 1.60) | tis+che | . |
| 1.47 (0.81; 2.65) | 1.31 (0.81; 2.14) | 0.95 (0.56; 1.62) | 1.04 (0.57; 1.89) | 1.12 (0.61; 2.05) | **2.04 (1.33; 3.13)** | 1.78 (0.95; 3.32) | 1.51 (0.84; 2.73) | **2.55 (1.42; 4.60)** | 1.47 (0.81; 2.65) | **2.15 (1.31; 3.55)** | 1.36 (0.81; 2.28) | 1.67 (0.99; 2.82) | 1.37 (0.76; 2.46) | 1.54 (0.92; 2.59) | 1.14 (0.68; 1.90) | 1.64 (0.84; 3.19) | 0.88 (0.47; 1.63) | 1.08 (0.59; 1.99) | 1.04 (0.61; 1.77) | 0.98 (0.54; 1.79) | 1.02 (0.61; 1.69) | tor+che |

**Supplementary Table 5.** Pairwise comparisons from the Network Meta-analysis on ORR. Each cell of the efficacy contains the pooled HR and 95% confidence intervals for ORR; significant results are in bold.

| ate | . | . | . | . |  | . | . | . | . | . | . | . | . | . | . | . | . | . | . | . |
| --- | --- | --- | --- | --- | --- | --- | --- | --- | --- | --- | --- | --- | --- | --- | --- | --- | --- | --- | --- | --- |
| 0.55 (0.30;1.02) | ate+che | . | . | . |  | . | . | . | . | . | . | . | . | . | . | . | . | . | . | . |
| **0.35 (0.18;0.68)** | 0.63 (0.38;1.05) | cam+che | . | . |  | . | . | . | . | . | . | . | . | . | . | . | . | . | . | . |
| **0.44 (0.21;0.93)** | 0.79 (0.43;1.45) | 1.25 (0.65;2.42) | cem | . |  | . | . | . | . | . | . | . | . | . | . | . | . | . | . | . |
| **0.37 (0.17;0.83)** | 0.68 (0.35;1.32) | 1.07 (0.52;2.19) | 0.85 (0.39;1.88) | cem+che |  | . | . | . | . | . | . | . | . | . | . | . | . | . | . | . |
| 0.97 (0.57;1.66) | **1.75 (1.30;2.36)** | **2.77 (1.85;4.14)** | **2.21 (1.31;3.74)** | **2.59 (1.43;4.70)** | che |  |  |  |  |  |  |  |  |  |  |  |  |  |  |  |
| 0.93 (0.41;2.08) | 1.68 (0.85;3.30) | **2.64 (1.28;5.48)** | 2.12 (0.95;4.72) | **2.48 (1.06;5.80)** | 0.96 (0.52;1.76) | dur |  | . | . | . | . | . | . | . | . | . | . | . | . | . |
| 0.85 (0.38;1.92) | 1.55 (0.79;3.04) | **2.44 (1.18;5.06)** | 1.96 (0.88;4.35) | 2.29 (0.98;5.35) | 0.88 (0.48;1.62) | 0.92 (0.51;1.69) | dur+tre | . | . | . | . | . | . | . | . | . | . | . | . | . |
| 1.06 (0.55;2.03) | **1.92 (1.20;3.08)** | **3.03 (1.76;5.22)** | **2.43 (1.28;4.59)** | **2.84 (1.42;5.71)** | 1.10 (0.76;1.58) | 1.15 (0.56;2.33) | 1.24 (0.61;2.52) | niv |  |  | . | . | . | . | . | . | . | . | . | . |
| **0.50 (0.26;0.96)** | 0.91 (0.57;1.46) | 1.44 (0.84;2.47) | 1.15 (0.61;2.18) | 1.35 (0.68;2.70) | **0.52 (0.36;0.75)** | 0.54 (0.27;1.10) | 0.59 (0.29;1.19) | **0.48 (0.30;0.74)** | niv+che |  | . | . | . | . | . | . | . | . | . | . |
| 0.72 (0.36;1.44) | 1.31 (0.78;2.22) | **2.07 (1.15;3.73)** | 1.66 (0.84;3.26) | 1.94 (0.93;4.04) | 0.75 (0.49;1.15) | 0.78 (0.37;1.65) | 0.85 (0.40;1.78) | 0.68 (0.43;1.08) | 1.44 (0.89;2.31) | niv+ipi | . | . | . | . | . | . | . | . | . | . |
| 0.54 (0.26;1.13) | 0.98 (0.54;1.77) | 1.54 (0.80;2.96) | 1.23 (0.59;2.57) | 1.45 (0.66;3.17) | **0.56 (0.33;0.93)** | 0.58 (0.26;1.29) | 0.63 (0.29;1.39) | **0.51 (0.27;0.95)** | 1.07 (0.57;2.00) | 0.74 (0.38;1.45) | niv+ipi+che | . | . | . | . | . | . | . | . | . |
| 0.75 (0.39;1.44) | 1.35 (0.84;2.18) | **2.13 (1.23;3.69)** | 1.71 (0.90;3.25) | 2.00 (0.99;4.03) | 0.77 (0.53;1.12) | 0.81 (0.39;1.64) | 0.87 (0.43;1.78) | 0.70 (0.42;1.18) | 1.48 (0.88;2.48) | 1.03 (0.58;1.82) | 1.38 (0.73;2.61) | pem | . |  | . | . | . | . | . | . |
| **0.31 (0.16;0.60)** | 0.56 (0.34;0.91) | 0.88 (0.50;1.54) | 0.71 (0.37;1.35) | 0.83 (0.41;1.68) | **0.32 (0.22;0.47)** | **0.33 (0.16;0.68)** | **0.36 (0.18;0.74)** | **0.29 (0.17;0.49)** | 0.61 (0.36;1.03) | **0.43 (0.24;0.76)** | 0.57 (0.30;1.08) | **0.41 (0.24;0.71)** | pem+che | . | . | . | . | . | . | . |
| 0.75 (0.32;1.72) | 1.35 (0.67;2.74) | **2.13 (1.00;4.54)** | 1.71 (0.75;3.90) | 2.00 (0.83;4.79) | 0.77 (0.41;1.46) | 0.81 (0.33;1.95) | 0.87 (0.36;2.11) | 0.70 (0.34;1.47) | 1.48 (0.71;3.08) | 1.03 (0.48;2.23) | 1.38 (0.61;3.14) | 1.00 (0.59;1.68) | **2.42 (1.15;5.11)** | pem+ipi | . | . | . | . | . | . |
| **0.30 (0.14;0.68)** | 0.55 (0.28;1.08) | 0.87 (0.42;1.79) | 0.70 (0.31;1.54) | 0.82 (0.35;1.89) | **0.31 (0.17;0.57)** | **0.33 (0.14;0.77)** | **0.36 (0.15;0.83)** | **0.29 (0.14;0.58)** | 0.60 (0.30;1.21) | **0.42 (0.20;0.88)** | 0.56 (0.26;1.24) | **0.41 (0.20;0.83)** | 0.99 (0.48;2.01) | **0.41 (0.17;0.98)** | pen+che | . | . | . | . | . |
| **0.43 (0.20;0.93)** | 0.79 (0.42;1.46) | 1.24 (0.63;2.43) | 0.99 (0.47;2.11) | 1.16 (0.52;2.60) | **0.45 (0.26;0.77)** | 0.47 (0.21;1.06) | 0.51 (0.22;1.14) | **0.41 (0.21;0.79)** | 0.86 (0.45;1.65) | 0.60 (0.30;1.20) | 0.80 (0.38;1.69) | 0.58 (0.30;1.12) | 1.41 (0.72;2.73) | 0.58 (0.25;1.35) | 1.42 (0.63;3.19) | ser+che | . | . | . | . |
| **0.50 (0.25;0.99)** | 0.91 (0.55;1.52) | 1.44 (0.80;2.57) | 1.15 (0.59;2.25) | 1.35 (0.65;2.79) | **0.52 (0.34;0.79)** | 0.54 (0.26;1.14) | 0.59 (0.28;1.23) | **0.47 (0.27;0.83)** | 1.00 (0.57;1.73) | 0.69 (0.38;1.27) | 0.93 (0.48;1.81) | 0.67 (0.38;1.18) | 1.63 (0.92;2.88) | 0.67 (0.31;1.45) | 1.65 (0.80;3.43) | 1.16 (0.58;2.31) | sin+che | . | . | . |
| **0.38 (0.17;0.82)** | 0.68 (0.36;1.28) | 1.07 (0.54;2.14) | 0.86 (0.40;1.85) | 1.01 (0.45;2.28) | **0.39 (0.22;0.68)** | **0.41 (0.18;0.93)** | **0.44 (0.19;1.00)** | **0.35 (0.18;0.69)** | 0.75 (0.38;1.45) | 0.52 (0.26;1.05) | 0.70 (0.33;1.49) | **0.50 (0.26;0.99)** | 1.22 (0.62;2.40) | 0.50 (0.22;1.18) | 1.24 (0.54;2.80) | 0.87 (0.40;1.89) | 0.75 (0.37;1.50) | sug+che | . | . |
| **0.38 (0.19;0.75)** | 0.69 (0.41;1.16) | 1.08 (0.60;1.95) | 0.87 (0.44;1.71) | 1.01 (0.49;2.11) | **0.39 (0.25;0.60)** | **0.41 (0.19;0.86)** | **0.44 (0.21;0.93)** | **0.36 (0.20;0.63)** | 0.75 (0.43;1.32) | **0.52 (0.28;0.96)** | 0.70 (0.36;1.37) | **0.51 (0.29;0.90)** | 1.23 (0.69;2.19) | 0.51 (0.23;1.10) | 1.24 (0.59;2.60) | 0.87 (0.44;1.75) | 0.75 (0.41;1.37) | 1.01 (0.50;2.04) | tis+che | . |
| **0.43 (0.20;0.94)** | 0.79 (0.42;1.48) | 1.24 (0.62;2.47) | 0.99 (0.46;2.14) | 1.16 (0.51;2.63) | **0.45 (0.26;0.78)** | 0.47 (0.20;1.07) | 0.51 (0.22;1.16) | **0.41 (0.21;0.80)** | 0.86 (0.44;1.67) | 0.60 (0.29;1.21) | 0.80 (0.38;1.72) | 0.58 (0.30;1.14) | 1.41 (0.71;2.78) | 0.58 (0.25;1.36) | 1.42 (0.63;3.23) | 1.00 (0.46;2.18) | 0.86 (0.43;1.73) | 1.15 (0.52;2.55) | 1.15 (0.56;2.33) | tor+che |

**Supplementary Table 6.** Pairwise comparisons from the Network Meta-analysis on TrAE(Grade ≥3). Each cell of the efficacy contains the pooled HR and 95% confidence intervals for TrAE(Grade ≥3); significant results are in bold.

| ate | . | . | . | . |  | . | . | . | . | . | . | . | . | . | . | . | . | . | . | . | . | . |
| --- | --- | --- | --- | --- | --- | --- | --- | --- | --- | --- | --- | --- | --- | --- | --- | --- | --- | --- | --- | --- | --- | --- |
| **0.13 (0.07; 0.25)** | ate+che | . | . | . |  | . | . | . | . | . | . | . | . | . | . | . | . | . | . | . | . | . |
| **0.14 (0.07; 0.28)** | 1.07 (0.64; 1.79) | cam+che | . | . |  | . | . | . | . | . | . | . | . | . | . | . | . | . | . | . | . | . |
| **0.40 (0.19; 0.87)** | **3.06 (1.68; 5.56)** | **2.85 (1.47; 5.54)** | cem | . |  | . | . | . | . | . | . | . | . | . | . | . | . | . | . | . | . | . |
| **0.14 (0.06; 0.31)** | 1.05 (0.55; 2.01) | 0.98 (0.48; 1.99) | **0.34 (0.16; 0.74)** | cem+che |  | . | . | . | . | . | . | . | . | . | . | . | . | . | . | . | . | . |
| **0.24 (0.13; 0.41)** | **1.79 (1.32; 2.42)** | **1.67 (1.10; 2.53)** | **0.58 (0.35; 0.98)** | 1.71 (0.96; 3.04) | che |  |  |  |  |  |  |  |  |  |  | . |  |  |  |  |  |  |
| 0.69 (0.32; 1.51) | **5.21 (2.79; 9.72)** | **4.86 (2.45; 9.65)** | 1.70 (0.80; 3.61) | **4.98 (2.25;11.00)** | **2.92 (1.69; 5.03)** | dur | . |  | . | . | . | . | . | . | . | . | . | . | . | . | . | . |
| **0.23 (0.11; 0.50)** | 1.77 (0.98; 3.21) | 1.66 (0.86; 3.20) | 0.58 (0.28; 1.20) | 1.69 (0.79; 3.66) | 0.99 (0.60; 1.65) | **0.34 (0.16; 0.72)** | dur+che | . |  | . | . | . | . | . | . | . | . | . | . | . | . | . |
| **0.41 (0.19; 0.87)** | **3.07 (1.68; 5.62)** | **2.87 (1.47; 5.59)** | 1.00 (0.48; 2.10) | **2.93 (1.35; 6.39)** | **1.72 (1.02; 2.90)** | 0.59 (0.34; 1.03) | 1.73 (0.83; 3.59) | dur+tre | . | . | . | . | . | . | . | . | . | . | . | . | . | . |
| **0.18 (0.08; 0.37)** | 1.33 (0.74; 2.40) | 1.24 (0.64; 2.39) | **0.43 (0.21; 0.90)** | 1.27 (0.59; 2.74) | 0.74 (0.45; 1.24) | **0.26 (0.12; 0.54)** | 0.75 (0.45; 1.25) | **0.43 (0.21; 0.90)** | dur+tre+che | . | . | . | . | . | . | . | . | . | . | . | . | . |
| 0.77 (0.40; 1.51) | **5.85 (3.63; 9.42)** | **5.46 (3.13; 9.52)** | **1.91 (1.01; 3.61)** | **5.59 (2.82;11.08)** | **3.27 (2.26; 4.73)** | 1.12 (0.58; 2.17) | **3.30 (1.76; 6.18)** | **1.91 (1.00; 3.61)** | **4.40 (2.35; 8.26)** | niv |  |  | . | . | . | . | . | . | . | . | . | . |
| **0.14 (0.07; 0.27)** | 1.04 (0.65; 1.67) | 0.97 (0.56; 1.69) | **0.34 (0.18; 0.64)** | 1.00 (0.50; 1.96) | **0.58 (0.41; 0.84)** | **0.20 (0.10; 0.38)** | 0.59 (0.31; 1.10) | **0.34 (0.18; 0.64)** | 0.78 (0.42; 1.46) | **0.18 (0.11; 0.28)** | niv+che |  | . | . | . | . | . | . | . | . | . | . |
| **0.33 (0.16; 0.67)** | **2.48 (1.46; 4.20)** | **2.31 (1.27; 4.22)** | 0.81 (0.41; 1.59) | **2.37 (1.15; 4.87)** | 1.39 (0.90; 2.14) | **0.48 (0.24; 0.95)** | 1.40 (0.71; 2.72) | 0.81 (0.41; 1.59) | 1.86 (0.95; 3.64) | **0.42 (0.26; 0.68)** | **2.38 (1.47; 3.83)** | niv+ipi | . | . | . | . | . | . | . | . | . | . |
| **0.15 (0.07; 0.33)** | 1.16 (0.64; 2.10) | 1.08 (0.56; 2.09) | **0.38 (0.18; 0.78)** | 1.11 (0.52; 2.39) | 0.65 (0.39; 1.08) | **0.22 (0.11; 0.47)** | 0.65 (0.32; 1.34) | **0.38 (0.18; 0.78)** | 0.87 (0.43; 1.79) | **0.20 (0.11; 0.37)** | 1.12 (0.60; 2.07) | **0.47 (0.24; 0.91)** | niv+ipi+che | . | . | . | . | . | . | . | . | . |
| 0.67 (0.34; 1.32) | **5.09 (3.13; 8.27)** | **4.75 (2.70; 8.34)** | 1.66 (0.88; 3.16) | **4.86 (2.43; 9.69)** | **2.85 (1.95; 4.16)** | 0.98 (0.50; 1.90) | **2.87 (1.52; 5.41)** | 1.66 (0.87; 3.16) | **3.83 (2.03; 7.23)** | 0.87 (0.51; 1.48) | **4.88 (2.89; 8.23)** | **2.05 (1.15; 3.66)** | **4.38 (2.32; 8.24)** | pem | . |  | . | . | . | . | . | . |
| **0.17 (0.09; 0.33)** | 1.27 (0.78; 2.07) | 1.19 (0.67; 2.09) | **0.42 (0.22; 0.79)** | 1.21 (0.61; 2.42) | 0.71 (0.48; 1.04) | **0.24 (0.13; 0.47)** | 0.72 (0.38; 1.35) | **0.41 (0.22; 0.79)** | 0.96 (0.51; 1.81) | **0.22 (0.13; 0.37)** | 1.22 (0.72; 2.06) | **0.51 (0.29; 0.91)** | 1.09 (0.58; 2.06) | **0.25 (0.15; 0.43)** | pem+che | . | . | . | . | . | . | . |
| **0.41 (0.17; 0.96)** | **3.09 (1.51; 6.32)** | **2.88 (1.33; 6.24)** | 1.01 (0.44; 2.32) | **2.95 (1.23; 7.03)** | 1.73 (0.90; 3.31) | 0.59 (0.25; 1.38) | 1.74 (0.76; 3.97) | 1.00 (0.44; 2.32) | **2.32 (1.02; 5.31)** | 0.53 (0.25; 1.11) | **2.96 (1.41; 6.23)** | 1.25 (0.57; 2.72) | **2.65 (1.16; 6.05)** | 0.61 (0.36; 1.03) | **2.43 (1.14; 5.17)** | pem+ipi | . | . | . | . | . | . |
| **0.25 (0.11; 0.58)** | 1.91 (0.97; 3.76) | 1.79 (0.86; 3.72) | 0.63 (0.28; 1.39) | 1.83 (0.79; 4.21) | 1.07 (0.59; 1.96) | **0.37 (0.16; 0.83)** | 1.08 (0.49; 2.38) | 0.62 (0.28; 1.39) | 1.44 (0.65; 3.17) | **0.33 (0.16; 0.66)** | 1.84 (0.91; 3.71) | 0.77 (0.37; 1.63) | 1.65 (0.75; 3.62) | **0.38 (0.18; 0.77)** | 1.51 (0.74; 3.08) | 0.62 (0.26; 1.51) | pen+che | . | . | . | . | . |
| **0.21 (0.09; 0.46)** | 1.58 (0.84; 2.97) | 1.47 (0.73; 2.95) | 0.52 (0.24; 1.10) | 1.51 (0.68; 3.36) | 0.88 (0.51; 1.54) | **0.30 (0.14; 0.66)** | 0.89 (0.42; 1.89) | 0.51 (0.24; 1.10) | 1.19 (0.56; 2.53) | **0.27 (0.14; 0.53)** | 1.51 (0.78; 2.94) | 0.64 (0.31; 1.29) | 1.36 (0.64; 2.88) | **0.31 (0.16; 0.61)** | 1.24 (0.63; 2.44) | 0.51 (0.22; 1.20) | 0.82 (0.36; 1.87) | ser+che | . | . | . | . |
| **0.20 (0.10; 0.41)** | 1.49 (0.86; 2.57) | 1.39 (0.75; 2.58) | **0.49 (0.25; 0.97)** | 1.42 (0.68; 2.97) | 0.83 (0.53; 1.31) | **0.29 (0.14; 0.58)** | 0.84 (0.42; 1.66) | **0.49 (0.24; 0.97)** | 1.12 (0.57; 2.22) | **0.25 (0.14; 0.46)** | 1.43 (0.80; 2.55) | 0.60 (0.32; 1.13) | 1.28 (0.65; 2.53) | **0.29 (0.16; 0.53)** | 1.17 (0.65; 2.13) | 0.48 (0.22; 1.07) | 0.78 (0.37; 1.66) | 0.95 (0.46; 1.94) | sin+che | . | . | . |
| **0.26 (0.12; 0.57)** | **1.95 (1.04; 3.69)** | 1.82 (0.91; 3.66) | 0.64 (0.30; 1.37) | 1.87 (0.84; 4.17) | 1.09 (0.63; 1.91) | **0.38 (0.17; 0.82)** | 1.10 (0.52; 2.35) | 0.64 (0.30; 1.37) | 1.47 (0.69; 3.13) | **0.33 (0.17; 0.65)** | 1.88 (0.96; 3.65) | 0.79 (0.39; 1.60) | 1.68 (0.79; 3.58) | **0.38 (0.20; 0.76)** | 1.54 (0.78; 3.03) | 0.63 (0.27; 1.49) | 1.02 (0.45; 2.33) | 1.24 (0.56; 2.73) | 1.31 (0.64; 2.69) | sug+che | . | . |
| **0.15 (0.07; 0.31)** | 1.12 (0.64; 1.95) | 1.04 (0.56; 1.95) | **0.37 (0.18; 0.73)** | 1.07 (0.51; 2.24) | 0.62 (0.39; 1.00) | **0.21 (0.10; 0.44)** | 0.63 (0.32; 1.26) | **0.36 (0.18; 0.73)** | 0.84 (0.42; 1.68) | **0.19 (0.11; 0.35)** | 1.07 (0.59; 1.93) | **0.45 (0.24; 0.85)** | 0.96 (0.48; 1.91) | 0.22 (0.12; 0.40) | 0.88 (0.48; 1.61) | **0.36 (0.16; 0.81)** | 0.58 (0.27; 1.25) | 0.71 (0.34; 1.47) | 0.75 (0.39; 1.44) | 0.57 (0.28; 1.18) | tis+che | . |
| **0.29 (0.12; 0.66)** | **2.17 (1.09; 4.35)** | 2.03 (0.96; 4.29) | 0.71 (0.32; 1.60) | 2.08 (0.89; 4.85) | 1.22 (0.65; 2.27) | **0.42 (0.18; 0.96)** | 1.22 (0.55; 2.74) | 0.71 (0.31; 1.60) | 1.64 (0.73; 3.66) | **0.37 (0.18; 0.77)** | 2.09 (1.01; 4.28) | 0.88 (0.41; 1.88) | 1.87 (0.84; 4.18) | **0.43 (0.21; 0.89)** | 1.71 (0.82; 3.56) | 0.70 (0.29; 1.74) | 1.14 (0.48; 2.71) | 1.38 (0.60; 3.18) | 1.46 (0.67; 3.15) | 1.11 (0.48; 2.57) | 1.95 (0.89; 4.24) | tor+che |

**Supplementary Table 7.** Pairwise comparisons from the Network Meta-analysis on OS for nsquamous NSCLC patients. Each cell of the efficacy contains the pooled HR and 95% confidence intervals for OS; significant results are in bold.

| ate | . | . | . | . | . | . |  | . | . | . | . | . | . | . | . | . | . | . | . | . |  |
| --- | --- | --- | --- | --- | --- | --- | --- | --- | --- | --- | --- | --- | --- | --- | --- | --- | --- | --- | --- | --- | --- |
| 0.79 (0.47; 1.33) | ate+bev+che | . |  | . | . | . |  | . | . | . | . | . | . | . | . | . | . | . | . | . |  |
| 0.75 (0.47; 1.19) | 0.95 (0.75; 1.22) | ate+che |  | . | . | . |  | . | . | . | . | . | . | . | . | . | . | . | . | . |  |
| 0.63 (0.39; 1.03) | **0.80 (0.67; 0.95)** | **0.84 (0.71; 1.00)** | bev+che | . | . | . |  | . | . | . |  | . | . | . | . | . | . | . | . | . |  |
| 0.85 (0.51; 1.43) | 1.08 (0.72; 1.61) | 1.13 (0.83; 1.55) | 1.35 (0.94; 1.93) | cam+che | . | . |  | . | . | . | . | . | . | . | . | . | . | . | . | . |  |
| 0.97 (0.57; 1.64) | 1.23 (0.81; 1.85) | 1.29 (0.92; 1.80) | **1.53 (1.05; 2.23)** | 1.14 (0.76; 1.71) | cem | . |  | . | . | . | . | . | . | . | . | . | . | . | . | . |  |
| 0.97 (0.57; 1.66) | 1.23 (0.81; 1.88) | 1.29 (0.91; 1.82) | **1.54 (1.04; 2.26)** | 1.14 (0.75; 1.73) | 1.00 (0.65; 1.55) | cem+che |  | . | . | . | . | . | . | . | . | . | . | . | . | . |  |
| **0.62 (0.40; 0.96)** | 0.79 (0.59; 1.05) | **0.83 (0.71; 0.96)** | 0.98 (0.79; 1.23) | **0.73 (0.55; 0.96)** | **0.64 (0.48; 0.87)** | **0.64 (0.47; 0.88)** | che |  |  |  |  |  |  |  |  |  |  |  |  |  |  |
| 0.76 (0.46; 1.24) | 0.96 (0.67; 1.38) | 1.01 (0.77; 1.32) | 1.20 (0.87; 1.65) | 0.89 (0.62; 1.27) | 0.78 (0.54; 1.14) | 0.78 (0.53; 1.15) | 1.22 (0.98; 1.52) | dur+che | . | . | . | . | . | . | . | . | . | . | . | . |  |
| 0.89 (0.54; 1.45) | 1.12 (0.78; 1.61) | 1.18 (0.91; 1.54) | **1.41 (1.03; 1.93)** | 1.04 (0.73; 1.49) | 0.92 (0.63; 1.33) | 0.91 (0.62; 1.34) | **1.43 (1.15; 1.78)** | 1.17 (0.86; 1.60) | dur+tre+che | . | . | . | . | . | . | . | . | . | . | . |  |
| **0.59 (0.37; 0.94)** | 0.75 (0.55; 1.04) | **0.79 (0.64; 0.98)** | 0.94 (0.72; 1.24) | **0.70 (0.51; 0.96)** | **0.61 (0.44; 0.86)** | **0.61 (0.43; 0.87)** | 0.96 (0.82; 1.12) | 0.79 (0.60; 1.03) | **0.67 (0.51; 0.88)** | niv | . | . | . | . | . | . | . | . | . | . |  |
| 0.85 (0.48; 1.51) | 1.08 (0.77; 1.53) | 1.14 (0.81; 1.60) | **1.35 (1.00; 1.82)** | 1.00 (0.63; 1.60) | 0.88 (0.55; 1.42) | 0.88 (0.54; 1.43) | 1.37 (0.95; 1.99) | 1.13 (0.73; 1.74) | 0.96 (0.62; 1.48) | 1.43 (0.96; 2.15) | niv+bev+che | . | . | . | . | . | . | . | . | . |  |
| 0.75 (0.47; 1.20) | 0.95 (0.68; 1.32) | 1.00 (0.80; 1.25) | 1.19 (0.90; 1.57) | 0.88 (0.64; 1.22) | 0.77 (0.55; 1.09) | 0.77 (0.54; 1.10) | **1.21 (1.02; 1.43)** | 0.99 (0.75; 1.31) | 0.84 (0.64; 1.11) | **1.26 (1.00; 1.58)** | 0.88 (0.58; 1.32) | niv+che | . | . | . | . | . | . | . | . |  |
| 0.81 (0.51; 1.28) | 1.02 (0.74; 1.41) | 1.07 (0.87; 1.33) | 1.28 (0.97; 1.68) | 0.95 (0.69; 1.30) | 0.83 (0.60; 1.17) | 0.83 (0.59; 1.18) | **1.30 (1.11; 1.52)** | 1.06 (0.81; 1.40) | 0.91 (0.69; 1.19) | **1.36 (1.09; 1.69)** | 0.95 (0.63; 1.42) | 1.08 (0.86; 1.35) | niv+ipi | . | . | . | . | . | . | . |  |
| 0.79 (0.49; 1.29) | 1.01 (0.71; 1.44) | 1.06 (0.82; 1.37) | 1.26 (0.93; 1.72) | 0.94 (0.66; 1.33) | 0.82 (0.57; 1.19) | 0.82 (0.56; 1.20) | **1.28 (1.04; 1.58)** | 1.05 (0.77; 1.43) | 0.90 (0.66; 1.22) | **1.34 (1.03; 1.74)** | 0.93 (0.61; 1.43) | 1.06 (0.81; 1.39) | 0.99 (0.76; 1.28) | niv+ipi+che | . | . | . | . | . | . |  |
| 1.07 (0.61; 1.88) | 1.36 (0.86; 2.14) | 1.42 (0.97; 2.09) | **1.70 (1.12; 2.58)** | 1.26 (0.80; 1.97) | 1.11 (0.70; 1.76) | 1.10 (0.69; 1.77) | **1.72 (1.21; 2.45)** | 1.41 (0.93; 2.15) | 1.21 (0.80; 1.83) | **1.80 (1.23; 2.65)** | 1.26 (0.75; 2.10) | 1.43 (0.97; 2.11) | 1.33 (0.90; 1.95) | 1.34 (0.89; 2.03) | pem | . |  | . | . | . |  |
| 1.03 (0.64; 1.66) | 1.31 (0.94; 1.84) | 1.38 (1.09; 1.74) | **1.64 (1.23; 2.19)** | 1.22 (0.87; 1.70) | 1.07 (0.75; 1.52) | 1.07 (0.74; 1.53) | **1.67 (1.39; 2.00)** | **1.37 (1.03; 1.82)** | 1.17 (0.88; 1.55) | **1.74 (1.37; 2.21)** | 1.21 (0.80; 1.84) | **1.38 (1.08; 1.77)** | **1.28 (1.01; 1.63)** | 1.30 (0.98; 1.72) | 0.97 (0.65; 1.44) | pem+che | . | . | . | . |  |
| 1.03 (0.55; 1.93) | 1.30 (0.76; 2.23) | 1.37 (0.85; 2.21) | 1.63 (0.98; 2.71) | 1.21 (0.71; 2.06) | 1.06 (0.62; 1.83) | 1.06 (0.61; 1.84) | **1.66 (1.05; 2.61)** | 1.36 (0.82; 2.25) | 1.16 (0.70; 1.92) | **1.73 (1.07; 2.80)** | 1.21 (0.67; 2.17) | 1.37 (0.85; 2.23) | 1.28 (0.79; 2.06) | 1.29 (0.78; 2.13) | 0.96 (0.72; 1.28) | 0.99 (0.61; 1.62) | pem+ipi | . | . | . |  |
| 0.95 (0.57; 1.59) | 1.21 (0.82; 1.79) | 1.27 (0.94; 1.72) | **1.51 (1.07; 2.14)** | 1.12 (0.76; 1.65) | 0.99 (0.66; 1.47) | 0.98 (0.65; 1.48) | **1.54 (1.18; 2.01)** | 1.26 (0.89; 1.78) | 1.08 (0.76; 1.52) | **1.61 (1.18; 2.18)** | 1.12 (0.71; 1.77) | 1.28 (0.93; 1.75) | 1.18 (0.87; 1.61) | 1.20 (0.86; 1.68) | 0.89 (0.57; 1.39) | 0.92 (0.67; 1.27) | 0.93 (0.55; 1.57) | sin+che | . | . |  |
| 0.86 (0.49; 1.50) | 1.09 (0.70; 1.71) | 1.15 (0.79; 1.66) | 1.37 (0.91; 2.06) | 1.01 (0.65; 1.58) | 0.89 (0.57; 1.40) | 0.89 (0.56; 1.41) | 1.39 (0.99; 1.95) | 1.14 (0.76; 1.71) | 0.97 (0.65; 1.46) | **1.45 (1.00; 2.11)** | 1.01 (0.61; 1.68) | 1.15 (0.79; 1.69) | 1.07 (0.73; 1.56) | 1.08 (0.73; 1.62) | 0.81 (0.49; 1.32) | 0.83 (0.57; 1.23) | 0.84 (0.47; 1.48) | 0.90 (0.59; 1.39) | sug+che | . |  |
| 0.91 (0.53; 1.55) | 1.16 (0.76; 1.76) | 1.22 (0.87; 1.70) | 1.45 (0.99; 2.11) | 1.07 (0.71; 1.62) | 0.94 (0.62; 1.45) | 0.94 (0.61; 1.46) | **1.47 (1.08; 1.99)** | 1.21 (0.83; 1.76) | 1.03 (0.71; 1.50) | **1.54 (1.09; 2.16)** | 1.07 (0.66; 1.73) | 1.22 (0.86; 1.73) | 1.13 (0.80; 1.59) | 1.15 (0.79; 1.66) | 0.85 (0.54; 1.36) | 0.88 (0.62; 1.26) | 0.89 (0.51; 1.53) | 0.96 (0.64; 1.43) | 1.06 (0.67; 1.67) | tis+che |  |
| 1.29 (0.71; 2.33) | **1.64 (1.00; 2.68)** | **1.72 (1.13; 2.63)** | **2.05 (1.30; 3.24)** | 1.52 (0.94; 2.47) | 1.34 (0.81; 2.20) | 1.33 (0.80; 2.21) | **2.08 (1.40; 3.10)** | **1.71 (1.08; 2.70)** | 1.46 (0.92; 2.30) | **2.18 (1.42; 3.34)** | 1.52 (0.88; 2.62) | **1.73 (1.12; 2.66)** | **1.60 (1.05; 2.46)** | **1.63 (1.04; 2.55)** | **1.60 (1.05; 2.44)** | 1.25 (0.81; 1.94) | **1.66 (1.00; 2.77)** | 1.35 (0.84; 2.19) | 1.50 (0.89; 2.54) | 1.42 (0.86; 2.34) | tor+che |

**Supplementary Table 8.** Pairwise comparisons from the Network Meta-analysis on PFS for nsquamous NSCLC patients. Each cell of the efficacy contains the pooled HR and 95% confidence intervals for PFS; significant results are in bold.

| ate+che | . | . | . |  | . | . | . | . | . | . | . | . | . | . | . | . |
| --- | --- | --- | --- | --- | --- | --- | --- | --- | --- | --- | --- | --- | --- | --- | --- | --- |
| 1.00 (0.73; 1.36) | cam+che | . | . |  | . | . | . | . | . | . | . | . | . | . | . | . |
| 1.06 (0.79; 1.42) | 1.06 (0.72; 1.56) | cem | . |  | . | . | . | . | . | . | . | . | . | . | . | . |
| 1.13 (0.82; 1.56) | 1.13 (0.75; 1.71) | 1.06 (0.71; 1.59) | cem+che |  | . | . | . | . | . | . | . | . | . | . | . | . |
| **0.60 (0.53; 0.68)** | **0.60 (0.45; 0.79)** | **0.56 (0.43; 0.73)** | **0.53 (0.39; 0.72)** | che |  |  |  |  |  |  |  |  |  |  |  |  |
| 0.78 (0.60; 1.01) | 0.78 (0.54; 1.12) | 0.73 (0.52; 1.04) | 0.69 (0.47; 1.00) | **1.30 (1.04; 1.63)** | dur+che | . | . | . | . | . | . | . | . | . | . | . |
| 0.91 (0.69; 1.19) | 0.91 (0.63; 1.32) | 0.85 (0.60; 1.22) | 0.80 (0.55; 1.18) | **1.52 (1.19; 1.93)** | 1.17 (0.84; 1.62) | dur+tre+che | . | . | . | . | . | . | . | . | . | . |
| **0.46 (0.36; 0.61)** | **0.47 (0.32; 0.67)** | **0.44 (0.31; 0.62)** | **0.41 (0.28; 0.60)** | **0.78 (0.61; 0.98)** | **0.60 (0.43; 0.83)** | **0.51 (0.37; 0.72)** | niv | . | . | . | . | . | . | . | . | . |
| 0.89 (0.71; 1.13) | 0.90 (0.63; 1.26) | 0.84 (0.60; 1.17) | 0.79 (0.55; 1.13) | **1.49 (1.22; 1.82)** | 1.15 (0.85; 1.55) | 0.99 (0.72; 1.35) | **1.93 (1.42; 2.62)** | niv+che | . | . | . | . | . | . | . | . |
| 0.83 (0.66; 1.05) | 0.83 (0.59; 1.18) | 0.78 (0.56; 1.09) | 0.74 (0.51; 1.06) | **1.39 (1.14; 1.70)** | 1.07 (0.79; 1.45) | 0.92 (0.67; 1.25) | **1.79 (1.32; 2.44)** | 0.93 (0.70; 1.23) | niv+ipi+che | . | . | . | . | . | . | . |
| 1.09 (0.76; 1.55) | 1.09 (0.71; 1.69) | 1.03 (0.67; 1.57) | 0.96 (0.62; 1.51) | **1.82 (1.30; 2.54)** | 1.40 (0.94; 2.10) | 1.20 (0.80; 1.81) | **2.35 (1.56; 3.53)** | 1.22 (0.83; 1.80) | 1.31 (0.89; 1.93) | pem | . |  | . | . | . | . |
| 1.20 (0.96; 1.49) | 1.20 (0.86; 1.67) | 1.13 (0.82; 1.55) | 1.06 (0.75; 1.50) | **2.00 (1.67; 2.39)** | **1.54 (1.15; 2.05)** | 1.32 (0.98; 1.78) | **2.58 (1.92; 3.46)** | **1.34 (1.03; 1.75)** | **1.44 (1.10; 1.88)** | 1.10 (0.75; 1.61) | pem+che | . | . | . | . | . |
| 0.97 (0.63; 1.50) | 0.97 (0.59; 1.60) | 0.92 (0.56; 1.49) | 0.86 (0.52; 1.43) | **1.62 (1.07; 2.45)** | 1.25 (0.78; 2.00) | 1.07 (0.66; 1.73) | **2.09 (1.30; 3.37)** | 1.09 (0.69; 1.72) | 1.17 (0.74; 1.85) | 0.89 (0.70; 1.14) | 0.81 (0.52; 1.27) | pem+ipi | . | . | . | . |
| 1.24 (0.91; 1.70) | 1.24 (0.83; 1.86) | 1.17 (0.79; 1.73) | 1.10 (0.73; 1.67) | **2.07 (1.56; 2.77)** | **1.60 (1.11; 2.30)** | 1.37 (0.94; 1.99) | **2.68 (1.85; 3.88)** | 1.39 (0.98; 1.97) | **1.49 (1.05; 2.12)** | 1.14 (0.73; 1.77) | 1.04 (0.74; 1.45) | 1.28 (0.77; 2.11) | sin+che | . | . | . |
| 1.01 (0.75; 1.38) | 1.02 (0.68; 1.51) | 0.96 (0.65; 1.41) | 0.90 (0.60; 1.35) | **1.69 (1.28; 2.25)** | 1.31 (0.91; 1.87) | 1.12 (0.77; 1.62) | **2.19 (1.52; 3.15)** | 1.14 (0.80; 1.60) | 1.22 (0.86; 1.72) | 0.93 (0.60; 1.44) | 0.85 (0.61; 1.18) | 1.04 (0.63; 1.72) | 0.82 (0.55; 1.22) | sug+che | . | . |
| 0.95 (0.69; 1.32) | 0.95 (0.63; 1.44) | 0.90 (0.60; 1.34) | 0.84 (0.55; 1.29) | **1.59 (1.17; 2.15)** | 1.22 (0.84; 1.78) | 1.05 (0.71; 1.54) | **2.05 (1.40; 3.00)** | 1.06 (0.74; 1.53) | 1.14 (0.80; 1.64) | 0.87 (0.56; 1.37) | 0.79 (0.56; 1.13) | 0.98 (0.59; 1.63) | 0.77 (0.50; 1.16) | 0.94 (0.62; 1.42) | tis+che | . |
| 1.01 (0.67; 1.53) | 1.02 (0.63; 1.64) | 0.96 (0.60; 1.53) | 0.90 (0.55; 1.47) | **1.69 (1.15; 2.50)** | 1.31 (0.83; 2.05) | 1.12 (0.71; 1.77) | **2.19 (1.39; 3.44)** | 1.14 (0.73; 1.76) | 1.22 (0.79; 1.89) | 0.93 (0.56; 1.56) | 0.85 (0.55; 1.30) | 1.04 (0.59; 1.84) | 0.82 (0.50; 1.32) | 1.00 (0.62; 1.62) | 1.07 (0.65; 1.75) | tor+che |

**Supplementary Table 9.** Pairwise comparisons from the Network Meta-analysis on OS for squamous NSCLC patients. Each cell of the efficacy contains the pooled HR and 95% confidence intervals for OS; significant results are in bold.

| ate | . | . | . | . |  | . | . | . | . | . | . | . | . | . | . | . | . | . | . | . |
| --- | --- | --- | --- | --- | --- | --- | --- | --- | --- | --- | --- | --- | --- | --- | --- | --- | --- | --- | --- | --- |
| 0.61 (0.24; 1.52) | ate+che | . | . | . |  | . | . | . | . | . | . | . | . | . | . | . | . | . | . | . |
| 1.02 (0.40; 2.62) | **1.67 (1.16; 2.42)** | cam+che | . | . |  | . | . | . | . | . | . | . | . | . | . | . | . | . | . | . |
| 1.11 (0.43; 2.85) | **1.82 (1.26; 2.63)** | 1.09 (0.70; 1.69) | cem | . |  | . | . | . | . | . | . | . | . | . | . | . | . | . | . | . |
| 0.92 (0.35; 2.41) | **1.51 (1.00; 2.28)** | 0.90 (0.56; 1.46) | 0.83 (0.51; 1.34) | cem+che |  | . | . | . | . | . | . | . | . | . | . | . | . | . | . | . |
| 0.56 (0.23; 1.37) | 0.92 (0.76; 1.12) | **0.55 (0.40; 0.75)** | **0.51 (0.37; 0.69)** | **0.61 (0.42; 0.88)** | che |  |  |  |  |  |  |  |  |  |  |  |  |  |  |  |
| 0.67 (0.26; 1.69) | 1.10 (0.78; 1.53) | **0.65 (0.43; 0.99)** | **0.60 (0.40; 0.91)** | 0.73 (0.46; 1.14) | 1.19 (0.91; 1.56) | dur+che | . | . | . | . | . | . | . | . | . | . | . | . | . | . |
| 0.64 (0.25; 1.62) | 1.05 (0.75; 1.45) | **0.63 (0.41; 0.94)** | **0.58 (0.38; 0.87)** | 0.69 (0.44; 1.09) | 1.14 (0.87; 1.48) | 0.95 (0.65; 1.40) | dur+tre+che | . | . | . | . | . | . | . | . | . | . | . | . | . |
| 0.71 (0.28; 1.79) | 1.17 (0.87; 1.58) | 0.70 (0.47; 1.03) | **0.64 (0.44; 0.95)** | 0.78 (0.50; 1.20) | **1.27 (1.01; 1.60)** | 1.07 (0.75; 1.53) | 1.12 (0.79; 1.60) | niv | . | . | . | . | . | . | . | . | . | . | . | . |
| 0.74 (0.29; 1.87) | 1.21 (0.87; 1.68) | 0.72 (0.48; 1.09) | **0.66 (0.44; 1.00)** | 0.80 (0.51; 1.26) | **1.31 (1.01; 1.71)** | 1.10 (0.76; 1.61) | 1.16 (0.79; 1.68) | 1.03 (0.73; 1.46) | niv+che | . | . | . | . | . | . | . | . | . | . | . |
| 0.89 (0.35; 2.24) | **1.46 (1.07; 1.99)** | 0.87 (0.59; 1.30) | 0.80 (0.54; 1.19) | 0.97 (0.63; 1.50) | **1.59 (1.25; 2.02)** | 1.33 (0.93; 1.91) | 1.40 (0.98; 2.00) | 1.25 (0.89; 1.74) | 1.21 (0.85; 1.72) | niv+ipi | . | . | . | . | . | . | . | . | . | . |
| 0.88 (0.34; 2.23) | 1.44 (1.02; 2.02) | 0.86 (0.56; 1.31) | 0.79 (0.52; 1.20) | 0.95 (0.60; 1.51) | 1.56 (1.18; 2.07) | 1.31 (0.89; 1.94) | 1.37 (0.93; 2.02) | 1.23 (0.85; 1.76) | 1.19 (0.81; 1.75) | 0.98 (0.68; 1.42) | niv+ipi+che | . | . | . | . | . | . | . | . | . |
| 0.74 (0.30; 1.84) | 1.21 (0.93; 1.58) | 0.73 (0.50; 1.04) | **0.67 (0.46; 0.96)** | 0.81 (0.54; 1.21) | **1.32 (1.10; 1.58)** | 1.11 (0.80; 1.54) | 1.16 (0.84; 1.60) | 1.04 (0.77; 1.39) | 1.00 (0.73; 1.38) | 0.83 (0.62; 1.12) | 0.84 (0.61; 1.18) | pem | . |  | . | . | . | . | . | . |
| 0.79 (0.32; 1.96) | 1.30 (0.99; 1.69) | 0.77 (0.54; 1.11) | 0.71 (0.50; 1.02) | 0.86 (0.57; 1.29) | **1.41 (1.17; 1.69)** | 1.18 (0.85; 1.64) | 1.24 (0.90; 1.71) | 1.11 (0.82; 1.48) | 1.07 (0.78; 1.48) | 0.89 (0.66; 1.20) | 0.90 (0.65; 1.26) | 1.07 (0.82; 1.38) | pem+che | . | . | . | . | . | . | . |
| 0.64 (0.23; 1.74) | 1.05 (0.63; 1.73) | 0.63 (0.36; 1.09) | 0.58 (0.33; 1.01) | 0.69 (0.39; 1.25) | 1.14 (0.72; 1.81) | 0.96 (0.56; 1.63) | 1.00 (0.59; 1.71) | 0.89 (0.53; 1.50) | 0.87 (0.51; 1.47) | 0.72 (0.43; 1.21) | 0.73 (0.42; 1.25) | 0.86 (0.56; 1.32) | 0.81 (0.49; 1.33) | pem+ipi | . | . | . | . | . | . |
| 1.02 (0.40; 2.62) | **1.67 (1.16; 2.42)** | 1.00 (0.64; 1.56) | 0.92 (0.59; 1.43) | 1.11 (0.69; 1.79) | **1.82 (1.33; 2.49)** | **1.53 (1.01; 2.31)** | **1.60 (1.06; 2.42)** | 1.43 (0.97; 2.11) | 1.38 (0.92; 2.08) | 1.15 (0.77; 1.70) | 1.16 (0.76; 1.77) | 1.38 (0.96; 1.98) | 1.29 (0.90; 1.86) | 1.60 (0.91; 2.80) | pen+che | . | . | . | . | . |
| 0.77 (0.30; 1.93) | 1.26 (0.93; 1.71) | 0.75 (0.51; 1.12) | 0.69 (0.47; 1.03) | 0.84 (0.54; 1.29) | **1.37 (1.08; 1.73)** | 1.15 (0.80; 1.65) | 1.21 (0.84; 1.72) | 1.08 (0.77; 1.50) | 1.04 (0.73; 1.48) | 0.86 (0.62; 1.21) | 0.88 (0.61; 1.26) | 1.04 (0.77; 1.40) | 0.97 (0.72; 1.31) | 1.20 (0.72; 2.02) | 0.75 (0.51; 1.12) | ser+che | . | . | . | . |
| 0.99 (0.36; 2.71) | 1.62 (0.97; 2.71) | 0.97 (0.55; 1.71) | 0.89 (0.51; 1.57) | 1.08 (0.59; 1.95) | **1.76 (1.10; 2.83)** | 1.48 (0.86; 2.55) | 1.55 (0.90; 2.67) | 1.38 (0.82; 2.34) | 1.34 (0.78; 2.31) | 1.11 (0.65; 1.89) | 1.13 (0.65; 1.96) | 1.34 (0.81; 2.22) | 1.25 (0.75; 2.08) | 1.55 (0.80; 3.00) | 0.97 (0.55; 1.71) | 1.29 (0.76; 2.18) | sin+che | . | . | . |
| 1.00 (0.38; 2.64) | **1.64 (1.07; 2.53)** | 0.98 (0.60; 1.61) | 0.90 (0.55; 1.48) | 1.09 (0.64; 1.85) | **1.79 (1.22; 2.62)** | 1.50 (0.94; 2.40) | 1.57 (0.98; 2.51) | 1.40 (0.90; 2.20) | 1.36 (0.85; 2.17) | 1.12 (0.72; 1.77) | 1.14 (0.71; 1.84) | 1.35 (0.88; 2.07) | 1.27 (0.83; 1.94) | 1.57 (0.86; 2.86) | 0.98 (0.60; 1.61) | 1.30 (0.83; 2.05) | 1.01 (0.55; 1.86) | sug+che | . | . |
| 0.96 (0.37; 2.50) | **1.59 (1.07; 2.34)** | 0.95 (0.60; 1.50) | 0.87 (0.55; 1.38) | 1.05 (0.64; 1.73) | **1.72 (1.23; 2.41)** | 1.45 (0.94; 2.23) | 1.52 (0.99; 2.33) | 1.35 (0.90; 2.03) | 1.31 (0.86; 2.01) | 1.09 (0.72; 1.64) | 1.10 (0.71; 1.71) | 1.31 (0.89; 1.91) | 1.22 (0.83; 1.79) | 1.51 (0.85; 2.68) | 0.95 (0.60; 1.50) | 1.26 (0.83; 1.90) | 0.98 (0.55; 1.75) | 0.96 (0.58; 1.61) | tis+che | . |
| 0.57 (0.21; 1.50) | 0.93 (0.60; 1.44) | **0.56 (0.34; 0.92)** | **0.51 (0.31; 0.85)** | 0.62 (0.36; 1.06) | 1.01 (0.68; 1.50) | 0.85 (0.53; 1.37) | 0.89 (0.55; 1.43) | 0.79 (0.50; 1.25) | 0.77 (0.48; 1.24) | 0.64 (0.40; 1.01) | 0.65 (0.40; 1.05) | 0.77 (0.49; 1.18) | 0.72 (0.46; 1.11) | 0.89 (0.48; 1.63) | **0.56 (0.34; 0.92)** | 0.74 (0.46; 1.17) | 0.57 (0.31; 1.06) | **0.57 (0.33; 0.98)** | **0.59 (0.35; 0.99)** | tor+che |

**Supplementary Table 10.** Pairwise comparisons from the Network Meta-analysis on PFS for squamous NSCLC patients. Each cell of the efficacy contains the pooled HR and 95% confidence intervals for PFS; significant results are in bold.

| ate+che | . | . | . |  | . | . | . | . | . | . | . | . | . | . | . | . | . | . |
| --- | --- | --- | --- | --- | --- | --- | --- | --- | --- | --- | --- | --- | --- | --- | --- | --- | --- | --- |
| **2.00 (1.49; 2.69)** | cam+che | . | . |  | . | . | . | . | . | . | . | . | . | . | . | . | . | . |
| **1.69 (1.19; 2.41)** | 0.85 (0.57; 1.25) | cem | . |  | . | . | . | . | . | . | . | . | . | . | . | . | . | . |
| 1.32 (0.92; 1.90) | **0.66 (0.44; 0.99)** | 0.78 (0.50; 1.22) | cem+che |  | . | . | . | . | . | . | . | . | . | . | . | . | . | . |
| **0.74 (0.62; 0.88)** | **0.37 (0.29; 0.47)** | **0.44 (0.32; 0.59)** | **0.56 (0.41; 0.77)** | che |  |  |  |  |  |  |  |  |  |  |  |  |  |  |
| 1.09 (0.79; 1.50) | **0.54 (0.38; 0.78)** | **0.64 (0.43; 0.97)** | 0.82 (0.54; 1.26) | **1.47 (1.12; 1.93)** | dur+che | . | . | . | . | . | . | . | . | . | . | . | . | . |
| 0.96 (0.69; 1.33) | **0.48 (0.33; 0.69)** | **0.57 (0.38; 0.86)** | 0.73 (0.48; 1.11) | 1.30 (0.98; 1.71) | 0.88 (0.60; 1.30) | dur+tre+che | . | . | . | . | . | . | . | . | . | . | . | . |
| 0.89 (0.56; 1.41) | **0.45 (0.27; 0.73)** | **0.53 (0.31; 0.89)** | 0.67 (0.40; 1.15) | 1.20 (0.79; 1.84) | 0.82 (0.49; 1.36) | 0.93 (0.56; 1.54) | niv | . | . | . | . | . | . | . | . | . | . | . |
| **1.45 (1.01; 2.08)** | 0.73 (0.49; 1.08) | 0.86 (0.55; 1.33) | 1.10 (0.70; 1.73) | **1.96 (1.43; 2.70)** | 1.33 (0.88; 2.03) | 1.51 (0.99; 2.30) | 1.63 (0.96; 2.77) | niv+che | . | . | . | . | . | . | . | . | . | . |
| 1.23 (0.87; 1.75) | **0.62 (0.42; 0.91)** | 0.73 (0.47; 1.12) | 0.93 (0.60; 1.45) | **1.67 (1.23; 2.26)** | 1.13 (0.75; 1.71) | 1.28 (0.85; 1.94) | 1.38 (0.82; 2.33) | 0.85 (0.55; 1.32) | niv+ipi+che | . | . | . | . | . | . | . | . | . |
| **2.11 (1.01; 4.41)** | 1.06 (0.50; 2.25) | 1.25 (0.57; 2.72) | 1.60 (0.73; 3.50) | **2.86 (1.40; 5.84)** | 1.94 (0.90; 4.18) | **2.20 (1.02; 4.74)** | **2.37 (1.03; 5.44)** | 1.46 (0.67; 3.19) | 1.71 (0.79; 3.73) | pem | . |  | . | . | . | . | . | . |
| 1.19 (0.93; 1.52) | **0.60 (0.44; 0.80)** | **0.70 (0.49; 1.00)** | 0.90 (0.63; 1.30) | **1.61 (1.35; 1.92)** | 1.10 (0.79; 1.52) | 1.24 (0.89; 1.73) | 1.34 (0.85; 2.12) | 0.82 (0.57; 1.18) | 0.97 (0.68; 1.38) | 0.56 (0.27; 1.18) | pem+che | . | . | . | . | . | . | . |
| 2.16 (0.95; 4.91) | 1.08 (0.47; 2.50) | 1.27 (0.54; 3.01) | 1.63 (0.69; 3.88) | **2.92 (1.30; 6.51)** | 1.98 (0.85; 4.64) | 2.24 (0.96; 5.25) | 2.42 (0.98; 6.00) | 1.49 (0.63; 3.53) | 1.75 (0.74; 4.13) | 1.02 (0.71; 1.47) | 1.81 (0.79; 4.12) | pem+ipi | . | . | . | . | . | . |
| **1.72 (1.26; 2.36)** | 0.86 (0.60; 1.23) | 1.02 (0.68; 1.52) | 1.30 (0.86; 1.97) | **2.33 (1.79; 3.03)** | **1.58 (1.08; 2.31)** | **1.79 (1.22; 2.63)** | **1.93 (1.17; 3.18)** | 1.19 (0.78; 1.79) | 1.40 (0.93; 2.09) | 0.81 (0.38; 1.74) | **1.44 (1.05; 1.98)** | 0.80 (0.34; 1.86) | pen+che | . | . | . | . | . |
| **1.40 (1.05; 1.86)** | **0.70 (0.50; 0.98)** | 0.82 (0.56; 1.21) | 1.06 (0.71; 1.57) | **1.89 (1.49; 2.38)** | 1.28 (0.89; 1.84) | **1.45 (1.01; 2.09)** | 1.57 (0.97; 2.54) | 0.96 (0.65; 1.43) | 1.13 (0.77; 1.66) | 0.66 (0.31; 1.40) | 1.17 (0.87; 1.57) | 0.65 (0.28; 1.49) | 0.81 (0.57; 1.15) | ser+che | . | . | . | . |
| **1.38 (1.03; 1.85)** | **0.69 (0.49; 0.97)** | 0.82 (0.55; 1.20) | 1.04 (0.70; 1.56) | **1.87 (1.47; 2.37)** | 1.27 (0.88; 1.83) | **1.44 (1.00; 2.07)** | 1.55 (0.95; 2.52) | 0.95 (0.64; 1.42) | 1.12 (0.76; 1.65) | 0.65 (0.31; 1.39) | 1.16 (0.86; 1.56) | 0.64 (0.28; 1.48) | 0.80 (0.56; 1.15) | 0.99 (0.71; 1.38) | sin+che | . | . | . |
| **2.18 (1.48; 3.20)** | 1.09 (0.71; 1.66) | 1.29 (0.81; 2.04) | **1.65 (1.03; 2.64)** | **2.94 (2.08; 4.16)** | **2.00 (1.29; 3.11)** | **2.26 (1.45; 3.53)** | **2.44 (1.41; 4.22)** | 1.50 (0.94; 2.40) | **1.76 (1.11; 2.80)** | 1.03 (0.47; 2.28) | **1.82 (1.24; 2.69)** | 1.01 (0.42; 2.42) | 1.26 (0.82; 1.96) | **1.56 (1.03; 2.37)** | **1.58 (1.03; 2.40)** | sug+che | . | . |
| **1.68 (1.26; 2.23)** | 0.84 (0.60; 1.17) | 0.99 (0.68; 1.46) | 1.27 (0.86; 1.89) | **2.27 (1.81; 2.85)** | **1.54 (1.08; 2.21)** | **1.75 (1.22; 2.50)** | **1.88 (1.17; 3.05)** | 1.16 (0.78; 1.71) | 1.36 (0.93; 1.99) | 0.79 (0.38; 1.68) | **1.41 (1.06; 1.88)** | 0.78 (0.34; 1.80) | 0.98 (0.69; 1.38) | 1.20 (0.87; 1.67) | 1.22 (0.87; 1.69) | 0.77 (0.51; 1.17) | tis+che | . |
| 1.35 (0.88; 2.06) | 0.67 (0.43; 1.06) | 0.79 (0.48; 1.31) | 1.02 (0.61; 1.69) | **1.82 (1.23; 2.69)** | 1.24 (0.77; 1.99) | 1.40 (0.87; 2.26) | 1.51 (0.85; 2.69) | 0.93 (0.56; 1.54) | 1.09 (0.66; 1.79) | 0.64 (0.28; 1.44) | 1.13 (0.73; 1.73) | 0.62 (0.26; 1.52) | 0.78 (0.49; 1.25) | 0.96 (0.61; 1.52) | 0.97 (0.62; 1.54) | 0.62 (0.37; 1.04) | 0.80 (0.51; 1.26) | tor+che |

**Supplementary Table 11.** Pairwise comparisons from the Network Meta-analysis on OS for PD-L1 expression≥50%. Each cell of the efficacy contains the pooled HR and 95% confidence intervals for OS; significant results are in bold.

| ate | . | . | . | . | . | . |  | . | . | . | . | . | . | . | . | . | . | . | . | . | . |
| --- | --- | --- | --- | --- | --- | --- | --- | --- | --- | --- | --- | --- | --- | --- | --- | --- | --- | --- | --- | --- | --- |
| 1.27 (0.58; 2.75) | ate+bev+che | . |  | . | . | . |  | . | . | . | . | . | . | . | . | . | . | . | . | . | . |
| 1.17 (0.72; 1.89) | 0.92 (0.50; 1.69) | ate+che |  | . | . | . |  | . | . | . | . | . | . | . | . | . | . | . | . | . | . |
| 0.89 (0.46; 1.70) | 0.70 (0.46; 1.07) | 0.76 (0.49; 1.17) | bev+che | . | . | . |  | . | . | . | . | . |  | . | . | . | . | . | . | . | . |
| 1.58 (0.64; 3.92) | 1.25 (0.42; 3.71) | 1.36 (0.55; 3.34) | 1.79 (0.66; 4.86) | cam+che | . | . |  | . | . | . | . | . | . | . | . | . | . | . | . | . | . |
| 1.33 (0.84; 2.12) | 1.05 (0.49; 2.25) | 1.14 (0.73; 1.79) | 1.51 (0.81; 2.81) | 0.84 (0.35; 2.05) | cem | . |  | . | . | . | . | . | . | . | . | . | . | . | . | . | . |
| 1.36 (0.78; 2.37) | 1.07 (0.47; 2.43) | 1.16 (0.67; 2.01) | 1.53 (0.76; 3.08) | 0.86 (0.33; 2.20) | 1.02 (0.60; 1.73) | cem+che |  | . | . | . | . | . | . | . | . | . | . | . | . | . | . |
| 0.76 (0.53; 1.08) | 0.60 (0.30; 1.20) | **0.65 (0.47; 0.91)** | 0.86 (0.50; 1.48) | 0.48 (0.21; 1.11) | **0.57 (0.42; 0.77)** | **0.56 (0.36; 0.87)** | che |  |  |  |  |  |  |  |  |  |  |  |  |  |  |
| 1.00 (0.62; 1.61) | 0.79 (0.37; 1.70) | 0.86 (0.54; 1.36) | 1.13 (0.60; 2.13) | 0.63 (0.26; 1.55) | 0.75 (0.48; 1.16) | 0.74 (0.43; 1.26) | 1.32 (0.96; 1.81) | dur | . | . | . | . | . | . | . | . | . | . | . | . | . |
| 1.21 (0.74; 1.96) | 0.95 (0.44; 2.06) | 1.03 (0.65; 1.66) | 1.36 (0.72; 2.59) | 0.76 (0.31; 1.88) | 0.90 (0.58; 1.42) | 0.89 (0.51; 1.54) | **1.59 (1.14; 2.22)** | 1.21 (0.76; 1.92) | dur+che | . | . | . | . | . | . | . | . | . | . | . | . |
| 0.99 (0.61; 1.59) | 0.78 (0.36; 1.68) | 0.85 (0.53; 1.35) | 1.11 (0.59; 2.10) | 0.62 (0.25; 1.53) | 0.74 (0.48; 1.15) | 0.73 (0.42; 1.25) | 1.30 (0.94; 1.80) | 0.99 (0.63; 1.55) | 0.82 (0.51; 1.30) | dur+tre | . | . | . | . | . | . | . | . | . | . | . |
| 1.17 (0.73; 1.88) | 0.92 (0.43; 1.98) | 1.00 (0.63; 1.59) | 1.32 (0.70; 2.49) | 0.74 (0.30; 1.81) | 0.88 (0.56; 1.36) | 0.86 (0.50; 1.48) | **1.54 (1.12; 2.12)** | 1.17 (0.74; 1.84) | 0.97 (0.61; 1.54) | 1.18 (0.75; 1.87) | dur+tre+che | . | . | . | . | . | . | . | . | . | . |
| 0.90 (0.61; 1.33) | 0.71 (0.35; 1.46) | 0.78 (0.54; 1.12) | 1.02 (0.58; 1.81) | 0.57 (0.24; 1.34) | **0.68 (0.48; 0.96)** | 0.67 (0.42; 1.06) | **1.19 (1.01; 1.40)** | 0.90 (0.63; 1.29) | 0.75 (0.52; 1.09) | 0.92 (0.64; 1.32) | 0.77 (0.54; 1.11) | niv | . | . |  | . | . | . | . | . | . |
| 1.07 (0.47; 2.40) | 0.84 (0.44; 1.61) | 0.92 (0.48; 1.75) | 1.20 (0.74; 1.95) | 0.67 (0.22; 2.05) | 0.80 (0.36; 1.76) | 0.79 (0.34; 1.84) | 1.40 (0.68; 2.91) | 1.07 (0.48; 2.37) | 0.88 (0.40; 1.97) | 1.08 (0.49; 2.40) | 0.91 (0.41; 2.02) | 1.18 (0.56; 2.49) | niv+bev+che | . | . | . | . | . | . | . | . |
| 1.13 (0.66; 1.95) | 0.90 (0.40; 2.01) | 0.97 (0.57; 1.66) | 1.28 (0.64; 2.54) | 0.72 (0.28; 1.82) | 0.85 (0.51; 1.42) | 0.84 (0.46; 1.53) | 1.49 (0.99; 2.26) | 1.13 (0.67; 1.91) | 0.94 (0.55; 1.60) | 1.15 (0.68; 1.95) | 0.97 (0.57; 1.64) | 1.25 (0.80; 1.96) | 1.06 (0.46; 2.46) | niv+che | . | . | . | . | . | . | . |
| 1.15 (0.77; 1.71) | 0.91 (0.44; 1.86) | 0.99 (0.67; 1.44) | 1.30 (0.73; 2.31) | 0.73 (0.31; 1.71) | 0.86 (0.60; 1.23) | 0.85 (0.53; 1.36) | **1.51 (1.25; 1.82)** | 1.15 (0.79; 1.66) | 0.95 (0.65; 1.40) | 1.16 (0.80; 1.69) | 0.98 (0.68; 1.42) | **1.27 (1.06; 1.53)** | 1.08 (0.51; 2.28) | 1.01 (0.64; 1.60) | niv+ipi | . | . | . | . | . | . |
| 1.13 (0.68; 1.89) | 0.90 (0.41; 1.97) | 0.97 (0.59; 1.60) | 1.28 (0.66; 2.48) | 0.72 (0.29; 1.79) | 0.85 (0.53; 1.38) | 0.84 (0.47; 1.48) | **1.49 (1.03; 2.17)** | 1.13 (0.69; 1.85) | 0.94 (0.57; 1.55) | 1.15 (0.70; 1.88) | 0.97 (0.59; 1.59) | 1.25 (0.83; 1.88) | 1.06 (0.47; 2.41) | 1.00 (0.57; 1.75) | 0.99 (0.65; 1.50) | niv+ipi+che | . | . | . | . | . |
| 1.15 (0.79; 1.68) | 0.91 (0.45; 1.85) | 0.99 (0.69; 1.42) | 1.30 (0.74; 2.29) | 0.73 (0.31; 1.70) | 0.86 (0.62; 1.21) | 0.85 (0.54; 1.34) | **1.51 (1.31; 1.75)** | 1.15 (0.81; 1.63) | 0.95 (0.66; 1.37) | 1.17 (0.82; 1.66) | 0.98 (0.69; 1.40) | **1.27 (1.02; 1.58)** | 1.08 (0.51; 2.27) | 1.01 (0.65; 1.57) | 1.00 (0.79; 1.27) | 1.01 (0.68; 1.51) | pem | . |  | . | . |
| 1.12 (0.73; 1.72) | 0.88 (0.42; 1.84) | 0.96 (0.63; 1.45) | 1.26 (0.69; 2.30) | 0.71 (0.29; 1.69) | 0.84 (0.57; 1.24) | 0.82 (0.50; 1.36) | **1.47 (1.15; 1.88)** | 1.12 (0.75; 1.67) | 0.93 (0.61; 1.40) | 1.13 (0.75; 1.70) | 0.96 (0.64; 1.43) | 1.24 (0.92; 1.66) | 1.05 (0.48; 2.26) | 0.99 (0.61; 1.60) | 0.97 (0.71; 1.33) | 0.99 (0.63; 1.54) | 0.97 (0.73; 1.29) | pem+che | . | . | . |
| 1.07 (0.68; 1.67) | 0.84 (0.40; 1.78) | 0.91 (0.59; 1.41) | 1.20 (0.65; 2.22) | 0.67 (0.28; 1.63) | 0.80 (0.53; 1.21) | 0.78 (0.47; 1.32) | **1.40 (1.06; 1.85)** | 1.07 (0.70; 1.63) | 0.88 (0.57; 1.37) | 1.08 (0.70; 1.66) | 0.91 (0.60; 1.39) | 1.18 (0.85; 1.63) | 1.00 (0.46; 2.18) | 0.94 (0.57; 1.55) | 0.93 (0.66; 1.30) | 0.94 (0.59; 1.50) | 0.93 (0.73; 1.18) | 0.95 (0.66; 1.38) | pem+ipi | . | . |
| 1.33 (0.73; 2.43) | 1.05 (0.45; 2.46) | 1.14 (0.63; 2.06) | 1.51 (0.72; 3.13) | 0.84 (0.32; 2.22) | 1.00 (0.56; 1.78) | 0.98 (0.51; 1.89) | **1.75 (1.08; 2.86)** | 1.33 (0.74; 2.39) | 1.11 (0.61; 2.00) | 1.35 (0.75; 2.43) | 1.14 (0.64; 2.04) | 1.47 (0.88; 2.47) | 1.25 (0.52; 3.01) | 1.18 (0.62; 2.23) | 1.16 (0.69; 1.96) | 1.18 (0.64; 2.17) | 1.16 (0.70; 1.93) | 1.19 (0.69; 2.06) | 1.25 (0.71; 2.20) | sug+che | . |
| 0.93 (0.39; 2.20) | 0.73 (0.26; 2.10) | 0.80 (0.34; 1.88) | 1.05 (0.40; 2.74) | 0.59 (0.18; 1.85) | 0.70 (0.30; 1.62) | 0.68 (0.28; 1.69) | 1.22 (0.55; 2.69) | 0.93 (0.39; 2.18) | 0.77 (0.33; 1.82) | 0.94 (0.40; 2.21) | 0.79 (0.34; 1.86) | 1.02 (0.46; 2.30) | 0.87 (0.30; 2.55) | 0.82 (0.33; 2.00) | 0.81 (0.36; 1.82) | 0.82 (0.34; 1.96) | 0.81 (0.36; 1.80) | 0.83 (0.36; 1.90) | 0.87 (0.38; 2.02) | 0.70 (0.27; 1.76) | tor+che |

**Supplementary Table 12.** Pairwise comparisons from the Network Meta-analysis on PFS for PD-L1 expression≥50%. Each cell of the efficacy contains the pooled HR and 95% confidence intervals for PFS; significant results are in bold.

| ate | . | . | . | . |  | . | . | . | . | . | . | . | . | . | . | . |
| --- | --- | --- | --- | --- | --- | --- | --- | --- | --- | --- | --- | --- | --- | --- | --- | --- |
| 1.24 (0.81; 1.90) | ate+che | . | . | . |  | . | . | . | . | . | . | . | . | . | . | . |
| **1.83 (1.01; 3.33)** | 1.48 (0.83; 2.65) | cam+che | . | . |  | . | . | . | . | . | . | . | . | . | . | . |
| 1.09 (0.74; 1.62) | 0.88 (0.61; 1.27) | 0.60 (0.34; 1.04) | cem | . |  | . | . | . | . | . | . | . | . | . | . | . |
| 1.23 (0.73; 2.06) | 0.99 (0.60; 1.63) | 0.67 (0.35; 1.28) | 1.13 (0.71; 1.79) | cem+che |  | . | . | . | . | . | . | . | . | . | . | . |
| **0.59 (0.43; 0.81)** | **0.48 (0.36; 0.64)** | **0.32 (0.19; 0.53)** | **0.54 (0.43; 0.68)** | **0.48 (0.32; 0.72)** | che |  |  |  |  |  |  |  |  |  |  |  |
| 0.74 (0.52; 1.05) | **0.59 (0.43; 0.83)** | **0.40 (0.24; 0.68)** | **0.67 (0.51; 0.89)** | **0.60 (0.39; 0.93)** | **1.25 (1.06; 1.47)** | niv |  | . | . | . | . | . | . | . | . | . |
| 0.95 (0.66; 1.37) | 0.77 (0.54; 1.08) | **0.52 (0.30; 0.89)** | 0.87 (0.65; 1.17) | 0.77 (0.49; 1.21) | **1.61 (1.34; 1.94)** | **1.29 (1.07; 1.55)** | niv+ipi | . | . | . | . | . | . | . | . | . |
| 1.00 (0.62; 1.61) | 0.81 (0.51; 1.28) | 0.55 (0.29; 1.01) | 0.92 (0.60; 1.40) | 0.81 (0.47; 1.40) | **1.69 (1.18; 2.43)** | 1.36 (0.92; 2.01) | 1.05 (0.70; 1.58) | niv+ipi+che | . | . | . | . | . | . | . | . |
| 0.81 (0.58; 1.15) | **0.66 (0.48; 0.91)** | **0.44 (0.26; 0.75)** | **0.75 (0.57; 0.98)** | 0.66 (0.43; 1.02) | **1.38 (1.20; 1.59)** | 1.11 (0.89; 1.38) | 0.86 (0.68; 1.09) | 0.81 (0.55; 1.20) | pem | . |  | . | . | . | . | . |
| 1.46 (0.98; 2.18) | 1.18 (0.81; 1.72) | 0.80 (0.45; 1.40) | 1.34 (0.95; 1.87) | 1.19 (0.74; 1.91) | **2.48 (1.93; 3.17)** | **1.98 (1.47; 2.67)** | **1.54 (1.13; 2.10)** | 1.46 (0.94; 2.26) | **1.79 (1.34; 2.39)** | pem+che | . | . | . | . | . | . |
| 0.77 (0.51; 1.15) | **0.62 (0.42; 0.91)** | **0.42 (0.24; 0.74)** | **0.70 (0.50; 0.99)** | 0.63 (0.39; 1.01) | **1.30 (1.01; 1.68)** | 1.04 (0.77; 1.41) | 0.81 (0.59; 1.11) | 0.77 (0.50; 1.19) | 0.94 (0.77; 1.16) | **0.53 (0.37; 0.75)** | pem+ipi | . | . | . | . | . |
| 1.34 (0.78; 2.31) | 1.08 (0.64; 1.84) | 0.73 (0.37; 1.43) | 1.23 (0.74; 2.02) | 1.09 (0.60; 1.99) | **2.27 (1.46; 3.54)** | **1.82 (1.14; 2.92)** | 1.41 (0.87; 2.28) | 1.34 (0.76; 2.37) | **1.65 (1.03; 2.62)** | 0.92 (0.55; 1.53) | **1.74 (1.05; 2.90)** | ser+che | . | . | . | . |
| 1.54 (0.99; 2.39) | 1.24 (0.82; 1.89) | 0.84 (0.47; 1.51) | 1.41 (0.96; 2.07) | 1.25 (0.75; 2.08) | **2.61 (1.92; 3.55)** | **2.09 (1.48; 2.96)** | **1.62 (1.13; 2.32)** | 1.54 (0.96; 2.47) | **1.89 (1.35; 2.65)** | 1.05 (0.71; 1.57) | **2.00 (1.35; 2.98)** | 1.15 (0.67; 1.97) | sin+che | . | . | . |
| 1.44 (0.85; 2.43) | 1.16 (0.70; 1.93) | 0.78 (0.41; 1.51) | 1.32 (0.82; 2.12) | 1.17 (0.66; 2.09) | **2.44 (1.61; 3.70)** | **1.95 (1.25; 3.05)** | 1.52 (0.96; 2.39) | 1.44 (0.83; 2.49) | **1.77 (1.14; 2.74)** | 0.99 (0.61; 1.60) | **1.87 (1.15; 3.04)** | 1.07 (0.58; 1.97) | 0.93 (0.56; 1.57) | sug+che | . | . |
| 1.45 (0.91; 2.31) | 1.17 (0.75; 1.83) | 0.79 (0.43; 1.45) | 1.33 (0.88; 2.01) | 1.18 (0.69; 2.01) | **2.46 (1.74; 3.47)** | **1.97 (1.35; 2.88)** | **1.53 (1.03; 2.26)** | 1.45 (0.88; 2.38) | **1.78 (1.22; 2.58)** | 0.99 (0.65; 1.52) | **1.89 (1.23; 2.89)** | 1.08 (0.62; 1.90) | 0.94 (0.59; 1.49) | 1.01 (0.59; 1.73) | tis+che | . |
| 1.31 (0.71; 2.43) | 1.06 (0.58; 1.94) | 0.71 (0.34; 1.48) | 1.20 (0.67; 2.14) | 1.07 (0.55; 2.08) | **2.22 (1.31; 3.78)** | **1.78 (1.02; 3.10)** | 1.38 (0.79; 2.42) | 1.31 (0.69; 2.49) | 1.61 (0.93; 2.79) | 0.90 (0.50; 1.61) | 1.71 (0.95; 3.07) | 0.98 (0.49; 1.95) | 0.85 (0.46; 1.57) | 0.91 (0.46; 1.79) | 0.90 (0.48; 1.70) | tor+che |

**Supplementary Table 13.** Pairwise comparisons from the Network Meta-analysis on OS for PD-L1 1-49%. Each cell of the efficacy contains the pooled HR and 95% confidence intervals for OS; significant results are in bold.

| ate | . | . | . |  | . | . | . | . | . | . |
| --- | --- | --- | --- | --- | --- | --- | --- | --- | --- | --- |
| 0.87 (0.61; 1.22) | ate+che | . | . |  | . | . | . | . | . | . |
| 1.67 (0.82; 3.40) | 1.93 (0.97; 3.84) | cam+che | . |  | . | . | . | . | . | . |
| **1.74 (1.03; 2.95)** | **2.01 (1.22; 3.30)** | 1.04 (0.47; 2.30) | cem+che |  | . | . | . | . | . | . |
| 0.87 (0.66; 1.14) | 1.00 (0.82; 1.24) | **0.52 (0.27; 1.00)** | **0.50 (0.32; 0.79)** | che |  |  |  |  |  |  |
| 0.93 (0.65; 1.32) | 1.07 (0.79; 1.45) | 0.55 (0.28; 1.11) | **0.53 (0.32; 0.88)** | 1.06 (0.85; 1.33) | niv+ipi | . | . | . | . | . |
| 1.24 (0.83; 1.86) | **1.44 (1.00; 2.06)** | 0.74 (0.36; 1.52) | 0.71 (0.42; 1.23) | **1.43 (1.06; 1.92)** | 1.34 (0.92; 1.95) | niv+ipi+che | . | . | . | . |
| 0.99 (0.72; 1.36) | 1.14 (0.88; 1.49) | 0.59 (0.30; 1.16) | **0.57 (0.35; 0.92)** | 1.14 (0.97; 1.34) | 1.07 (0.81; 1.41) | 0.80 (0.57; 1.12) | pem | . | . | . |
| 1.39 (0.97; 1.98) | **1.60 (1.18; 2.17)** | 0.83 (0.41; 1.66) | 0.80 (0.48; 1.32) | **1.59 (1.27; 2.00)** | **1.50 (1.09; 2.06)** | 1.11 (0.77; 1.62) | **1.40 (1.06; 1.85)** | pem+che | . | . |
| 1.21 (0.69; 2.11) | 1.40 (0.82; 2.36) | 0.72 (0.32; 1.63) | 0.69 (0.36; 1.35) | 1.39 (0.86; 2.26) | 1.31 (0.76; 2.23) | 0.97 (0.55; 1.72) | 1.22 (0.73; 2.04) | 0.87 (0.51; 1.49) | sug+che | . |
| 1.21 (0.74; 1.96) | 1.40 (0.89; 2.19) | 0.72 (0.34; 1.56) | 0.69 (0.38; 1.27) | 1.39 (0.93; 2.07) | 1.31 (0.82; 2.07) | 0.97 (0.59; 1.60) | 1.22 (0.79; 1.88) | 0.87 (0.55; 1.38) | 1.00 (0.53; 1.88) | tor+che |

**Supplementary Table 14.** Pairwise comparisons from the Network Meta-analysis on PFS for PD-L1 1-49%. Each cell of the efficacy contains the pooled HR and 95% confidence intervals for PFS; significant results are in bold.

| ate | . | . | . |  | . | . | . | . | . | . | . |
| --- | --- | --- | --- | --- | --- | --- | --- | --- | --- | --- | --- |
| 0.92 (0.68; 1.25) | ate+che | . | . |  | . | . | . | . | . | . | . |
| 1.39 (0.93; 2.08) | **1.52 (1.05; 2.18)** | cam+che | . |  | . | . | . | . | . | . | . |
| 1.33 (0.87; 2.04) | 1.45 (0.98; 2.15) | 0.96 (0.60; 1.53) | cem+che |  | . | . | . | . | . | . | . |
| **0.64 (0.50; 0.82)** | **0.70 (0.58; 0.84)** | **0.46 (0.33; 0.63)** | **0.48 (0.34; 0.68)** | che |  |  |  |  |  |  |  |
| 0.93 (0.63; 1.37) | 1.01 (0.71; 1.44) | 0.67 (0.43; 1.03) | 0.70 (0.44; 1.10) | **1.45 (1.07; 1.97)** | niv+ipi+che | . | . | . | . | . | . |
| 1.09 (0.78; 1.52) | 1.19 (0.89; 1.58) | 0.78 (0.53; 1.15) | 0.82 (0.54; 1.23) | **1.70 (1.37; 2.13)** | 1.18 (0.81; 1.71) | pem+che | . | . | . | . | . |
| 0.90 (0.64; 1.26) | 0.98 (0.73; 1.32) | **0.65 (0.44; 0.95)** | 0.68 (0.45; 1.02) | **1.41 (1.12; 1.77)** | 0.97 (0.66; 1.42) | 0.83 (0.60; 1.14) | ser+che | . | . | . | . |
| 1.11 (0.72; 1.69) | 1.20 (0.81; 1.77) | 0.79 (0.50; 1.26) | 0.83 (0.51; 1.35) | **1.73 (1.23; 2.43)** | 1.19 (0.75; 1.89) | 1.01 (0.67; 1.52) | 1.23 (0.81; 1.85) | sin+che | . | . | . |
| 1.21 (0.75; 1.94) | 1.31 (0.84; 2.05) | 0.87 (0.52; 1.45) | 0.91 (0.53; 1.55) | **1.89 (1.26; 2.83)** | 1.30 (0.78; 2.17) | 1.11 (0.70; 1.76) | 1.34 (0.84; 2.14) | 1.09 (0.64; 1.86) | sug+che | . | . |
| 1.22 (0.75; 1.98) | 1.32 (0.84; 2.09) | 0.87 (0.52; 1.47) | 0.91 (0.53; 1.57) | **1.90 (1.25; 2.89)** | 1.31 (0.78; 2.20) | 1.12 (0.70; 1.79) | 1.35 (0.84; 2.18) | 1.10 (0.64; 1.89) | 1.01 (0.56; 1.81) | tis+che | . |
| 1.14 (0.75; 1.73) | 1.24 (0.85; 1.82) | 0.82 (0.52; 1.30) | 0.86 (0.53; 1.39) | **1.79 (1.28; 2.49)** | 1.23 (0.78; 1.94) | 1.05 (0.70; 1.56) | 1.27 (0.85; 1.90) | 1.03 (0.64; 1.67) | 0.95 (0.56; 1.60) | 0.94 (0.55; 1.60) | tor+che |

**Supplementary Table 15.** Pairwise comparisons from the Network Meta-analysis on OS for PD-L1＜1%. Each cell of the efficacy contains the pooled HR and 95% confidence intervals for OS; significant results are in bold.

| ate+bev+che | . |  | . | . | . | . | . | . | . | . | . | . | . | . | . | . | . |
| --- | --- | --- | --- | --- | --- | --- | --- | --- | --- | --- | --- | --- | --- | --- | --- | --- | --- |
| 0.94 (0.67; 1.31) | ate+che |  | . | . |  | . | . | . | . | . | . | . | . | . | . | . | . |
| 0.90 (0.71; 1.14) | 0.96 (0.76; 1.22) | bev+che | . | . |  | . | . | . | . |  | . | . | . | . | . | . | . |
| 1.21 (0.69; 2.12) | 1.29 (0.83; 2.02) | 1.35 (0.81; 2.24) | cam+che | . |  | . | . | . | . | . | . | . | . | . | . | . | . |
| 0.80 (0.46; 1.40) | 0.85 (0.54; 1.33) | 0.89 (0.54; 1.47) | 0.66 (0.37; 1.19) | cem+che |  | . | . | . | . | . | . | . | . | . | . | . | . |
| 0.75 (0.52; 1.09) | **0.80 (0.68; 0.95)** | 0.84 (0.62; 1.12) | **0.62 (0.41; 0.94)** | 0.94 (0.62; 1.42) | che |  |  |  |  |  |  |  |  |  |  |  |  |
| 0.64 (0.39; 1.04) | **0.68 (0.47; 0.97)** | 0.71 (0.46; 1.09) | **0.53 (0.31; 0.89)** | 0.80 (0.47; 1.34) | 0.85 (0.62; 1.16) | dur | . | . | . | . | . | . | . | . | . | . | . |
| 0.76 (0.48; 1.20) | 0.81 (0.59; 1.11) | 0.84 (0.57; 1.25) | 0.63 (0.38; 1.03) | 0.95 (0.58; 1.56) | 1.01 (0.77; 1.32) | 1.19 (0.79; 1.81) | dur+che | . | . | . | . | . | . | . | . | . | . |
| 1.03 (0.61; 1.73) | 1.10 (0.74; 1.63) | 1.14 (0.72; 1.81) | 0.85 (0.49; 1.47) | 1.29 (0.75; 2.22) | 1.37 (0.96; 1.96) | **1.62 (1.00; 2.60)** | 1.36 (0.87; 2.12) | dur+tre | . | . | . | . | . | . | . | . | . |
| 0.98 (0.61; 1.55) | 1.04 (0.75; 1.44) | 1.08 (0.73; 1.62) | 0.81 (0.49; 1.32) | 1.22 (0.74; 2.00) | 1.30 (0.99; 1.71) | **1.53 (1.01; 2.33)** | 1.29 (0.88; 1.88) | 0.95 (0.61; 1.48) | dur+tre+che | . | . | . | . | . | . | . | . |
| 1.07 (0.69; 1.66) | 1.14 (0.74; 1.77) | 1.19 (0.82; 1.72) | 0.88 (0.47; 1.65) | 1.34 (0.72; 2.51) | 1.43 (0.89; 2.28) | 1.68 (0.95; 2.96) | 1.41 (0.82; 2.42) | 1.04 (0.58; 1.88) | 1.10 (0.64; 1.89) | niv+bev+che | . | . | . | . | . | . | . |
| 0.95 (0.62; 1.43) | 1.01 (0.79; 1.29) | 1.05 (0.75; 1.47) | 0.78 (0.50; 1.22) | 1.18 (0.75; 1.85) | **1.26 (1.06; 1.50)** | **1.48 (1.03; 2.13)** | 1.25 (0.90; 1.71) | 0.92 (0.62; 1.36) | 0.97 (0.70; 1.34) | 0.88 (0.53; 1.46) | niv+che | . | . | . | . | . | . |
| 1.17 (0.76; 1.83) | 1.25 (0.94; 1.67) | 1.30 (0.90; 1.89) | 0.97 (0.60; 1.56) | 1.47 (0.91; 2.36) | **1.56 (1.24; 1.97)** | **1.84 (1.25; 2.73)** | **1.55 (1.09; 2.20)** | 1.14 (0.75; 1.74) | 1.20 (0.84; 1.72) | 1.10 (0.65; 1.85) | 1.24 (0.93; 1.66) | niv+ipi | . | . | . | . | . |
| 1.12 (0.71; 1.78) | 1.20 (0.87; 1.65) | 1.25 (0.84; 1.86) | 0.93 (0.56; 1.52) | 1.40 (0.85; 2.30) | **1.49 (1.14; 1.96)** | **1.76 (1.16; 2.67)** | **1.48 (1.01; 2.17)** | 1.09 (0.70; 1.71) | 1.15 (0.78; 1.69) | 1.05 (0.61; 1.80) | 1.19 (0.86; 1.64) | 0.96 (0.67; 1.37) | niv+ipi+che | . | . | . | . |
| 1.09 (0.71; 1.70) | 1.17 (0.88; 1.55) | 1.22 (0.84; 1.76) | 0.90 (0.56; 1.45) | 1.37 (0.85; 2.19) | **1.46 (1.16; 1.83)** | **1.72 (1.16; 2.54)** | **1.44 (1.01; 2.05)** | 1.06 (0.70; 1.62) | 1.12 (0.79; 1.60) | 1.02 (0.61; 1.72) | 1.16 (0.87; 1.54) | 0.93 (0.67; 1.29) | 0.98 (0.68; 1.39) | pem+che | . | . | . |
| 1.00 (0.56; 1.81) | 1.07 (0.66; 1.74) | 1.11 (0.65; 1.91) | 0.83 (0.45; 1.53) | 1.25 (0.68; 2.32) | 1.33 (0.85; 2.10) | 1.57 (0.90; 2.74) | 1.32 (0.78; 2.24) | 0.97 (0.55; 1.73) | 1.03 (0.60; 1.74) | 0.94 (0.49; 1.80) | 1.06 (0.65; 1.72) | 0.85 (0.51; 1.42) | 0.89 (0.53; 1.52) | 0.92 (0.55; 1.52) | sin+che | . | . |
| 1.14 (0.67; 1.95) | 1.21 (0.80; 1.85) | 1.27 (0.78; 2.05) | 0.94 (0.53; 1.65) | 1.42 (0.81; 2.51) | **1.52 (1.03; 2.22)** | **1.79 (1.09; 2.94)** | 1.50 (0.94; 2.40) | 1.11 (0.66; 1.87) | 1.17 (0.73; 1.87) | 1.06 (0.58; 1.95) | 1.20 (0.79; 1.84) | 0.97 (0.62; 1.52) | 1.02 (0.63; 1.63) | 1.04 (0.67; 1.62) | 1.14 (0.63; 2.06) | sug+che | . |
| 0.95 (0.51; 1.77) | 1.01 (0.60; 1.71) | 1.06 (0.60; 1.87) | 0.78 (0.41; 1.49) | 1.19 (0.63; 2.26) | 1.27 (0.77; 2.07) | 1.49 (0.83; 2.68) | 1.25 (0.72; 2.19) | 0.92 (0.50; 1.70) | 0.97 (0.56; 1.71) | 0.89 (0.45; 1.75) | 1.01 (0.60; 1.70) | 0.81 (0.47; 1.39) | 0.85 (0.48; 1.49) | 0.87 (0.51; 1.49) | 0.95 (0.49; 1.85) | 0.84 (0.45; 1.56) | tor+che |

**Supplementary Table 16.** Pairwise comparisons from the Network Meta-analysis on PFS for PD-L1＜1%. Each cell of the efficacy contains the pooled HR and 95% confidence intervals for PFS; significant results are in bold.

| ate+che | . | . |  | . | . | . | . | . | . | . | . | . |
| --- | --- | --- | --- | --- | --- | --- | --- | --- | --- | --- | --- | --- |
| 1.25 (0.91; 1.71) | cam+che | . |  | . | . | . | . | . | . | . | . | . |
| 0.95 (0.63; 1.44) | 0.76 (0.47; 1.23) | cem+che |  | . | . | . | . | . | . | . | . | . |
| **0.69 (0.60; 0.81)** | **0.56 (0.42; 0.74)** | 0.73 (0.50; 1.07) | che |  |  |  |  |  |  |  |  |  |
| 0.97 (0.73; 1.28) | 0.77 (0.54; 1.11) | 1.01 (0.65; 1.59) | **1.39 (1.10; 1.75)** | niv+che | . | . | . | . | . | . | . | . |
| 0.94 (0.71; 1.25) | 0.75 (0.52; 1.09) | 0.99 (0.63; 1.55) | **1.35 (1.06; 1.72)** | 0.97 (0.70; 1.36) | niv+ipi | . | . | . | . | . | . | . |
| 1.02 (0.74; 1.40) | 0.82 (0.55; 1.22) | 1.07 (0.67; 1.73) | **1.47 (1.11; 1.94)** | 1.06 (0.74; 1.52) | 1.09 (0.75; 1.57) | niv+ipi+che | . | . | . | . | . | . |
| 1.01 (0.78; 1.32) | 0.81 (0.57; 1.16) | 1.06 (0.68; 1.66) | **1.46 (1.17; 1.81)** | 1.05 (0.76; 1.45) | 1.08 (0.78; 1.49) | 0.99 (0.70; 1.41) | pem+che | . | . | . | . | . |
| **1.51 (1.00; 2.29)** | 1.21 (0.75; 1.95) | 1.59 (0.92; 2.74) | **2.17 (1.48; 3.20)** | **1.57 (1.00; 2.46)** | **1.61 (1.02; 2.53)** | 1.48 (0.92; 2.38) | 1.49 (0.96; 2.32) | ser+che | . | . | . | . |
| 1.18 (0.83; 1.66) | 0.94 (0.62; 1.43) | 1.23 (0.75; 2.02) | **1.69 (1.24; 2.30)** | 1.22 (0.83; 1.79) | 1.25 (0.85; 1.85) | 1.15 (0.76; 1.74) | 1.16 (0.79; 1.69) | 0.78 (0.47; 1.28) | sin+che | . | . | . |
| 1.22 (0.85; 1.74) | 0.98 (0.64; 1.50) | 1.28 (0.78; 2.12) | **1.75 (1.27; 2.42)** | 1.26 (0.85; 1.88) | 1.30 (0.87; 1.94) | 1.19 (0.78; 1.83) | 1.20 (0.82; 1.77) | 0.81 (0.49; 1.33) | 1.04 (0.66; 1.62) | sug+che | . | . |
| 0.99 (0.71; 1.39) | 0.80 (0.53; 1.20) | 1.04 (0.64; 1.70) | **1.43 (1.06; 1.93)** | 1.03 (0.70; 1.51) | 1.06 (0.72; 1.55) | 0.97 (0.65; 1.46) | 0.98 (0.68; 1.42) | 0.66 (0.40; 1.07) | 0.85 (0.55; 1.30) | 0.82 (0.53; 1.26) | tis+che | . |
| 1.48 (0.96; 2.27) | 1.19 (0.73; 1.93) | 1.55 (0.89; 2.70) | **2.13 (1.43; 3.17)** | 1.53 (0.97; 2.43) | 1.57 (0.99; 2.51) | 1.45 (0.89; 2.35) | 1.46 (0.93; 2.30) | 0.98 (0.56; 1.70) | 1.26 (0.76; 2.08) | 1.21 (0.73; 2.02) | 1.49 (0.90; 2.45) | tor+che |
